# Supplementary material for: Synthesis of the Carbohydrate Moiety of Glycoproteins from the Parasite Echinococcus granulosus and Their Antigenicity against Human Sera
Source: Molecules. 2021 Sep 17;26(18):5652. doi: 10.3390/molecules26185652 (PMC8468584; doi:10.3390/molecules26185652)
Supplement: Supplementary file 1 [file molecules-26-05652-s001.zip › molecules-1338029-supplementary.pdf]

Synthesis of the Carbohydrate Moiety of Glycoproteins from the Parasite *Echinococcus granulosus* and their Antigenicity against Human Sera

Noriyasu Hada [1,2,*], Tokio Morita[2], Hisashi Ueda [2], Kazuki, Masuda [2], Hiromi Nakane [2], Mami Ogane [2], Kimiaki Yamano[3], Frank Schweizer [4] and Fumiyuki Kiuchi[2]

1 Faculty of Pharmaceutical Sciences, Tokyo University of Science, 2641 Yamazaki, Noda-shi, Chiba 278-8510, Japan
2 Faculty of Pharmacy, Keio University, 1-5-30 Shibakoen, Minato-ku, Tokyo 105-8512, Japan
3 Hokkaido Institute of Public Health, Kita-19, Nishi-12, Kita-ku, Sapporo 060-0819, Japan
4 Department of Chemistry and Biochemistry, Faculty of Science, University of Manitoba, Winnipeg, MB R3T 2N2, Canada
* Correspondence: e-mail: hada@rs.tus.ac.jp; Tel.: +81-47-121-3612

Supplementary Information

Sample ID: s_20110524_01
File: s_20110524_01/data/Proton_Minsw_01.fid

Pulse Sequence: s2pul

Solvent: cdcl3
Ambient temperature
Operator: walkup
File: Proton_Minsw_01
INOVA-500 "INOVA500"

Relax. delay 1.500 sec
Pulse 45.0 degrees
Acq. time 3.500 sec
Width 5256.2 Hz
16 repetitions
OBSERVE H1, 500.1967504 MHz
DATA PROCESSING
Line broadening 0.3 Hz
FT size 65536
Total time 1 min, 20 sec

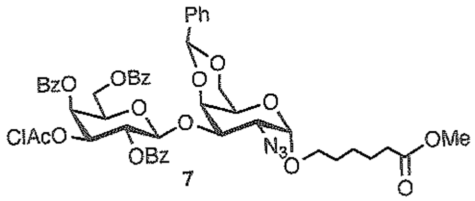

7

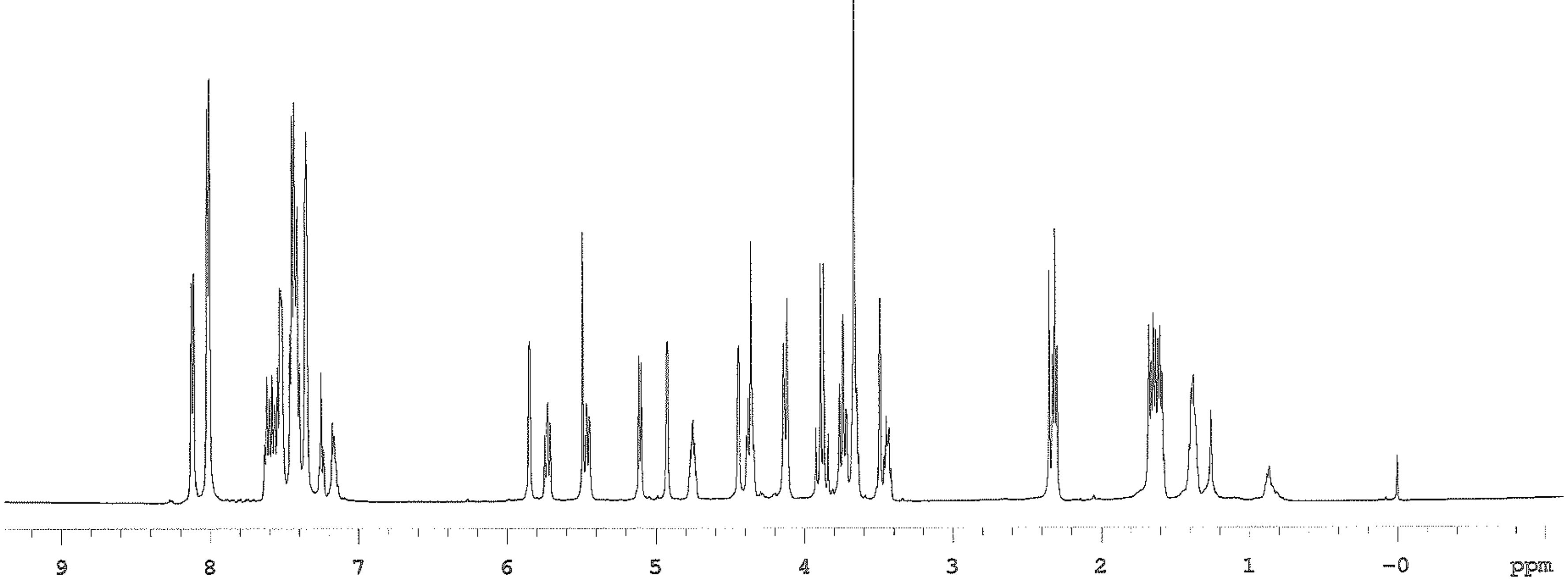

Sample ID: s_20110524_01
File: s_20110524_01/data/Carbon_02.fid

Pulse Sequence: s2pul

Solvent: cdcl3
Ambient temperature
Operator: walkup
File: Carbon_02
INOVA-500 "INOVA500"

Relax. delay 0.700 sec
Pulse 45.0 degrees
Acq. time 1.300 sec
Width 30188.7 Hz
512 repetitions
OBSERVE C13, 125.7745800 MHz
DECOUPLE H1, 500.1992491 MHz
Power 41 dB
continuously on
WALTZ-16 modulated
DATA PROCESSING
Line broadening 1.0 Hz
FT size 131072
Total time 17 min, 8 sec

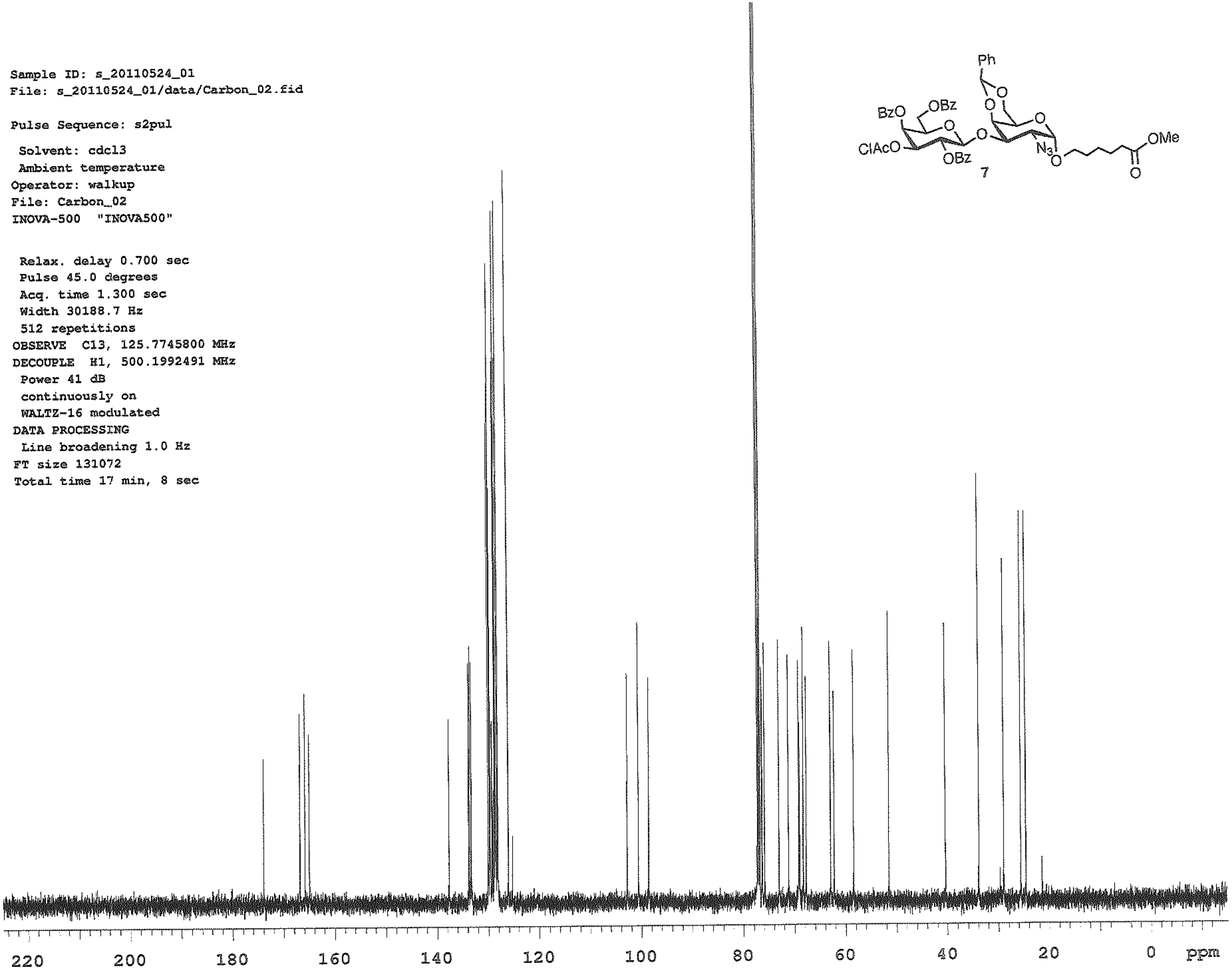

Sample ID: s_20110531_01
File: s_20110531_01/data/Proton_Minsw_01.fid

Pulse Sequence: s2pul

Solvent: cdcl3
Ambient temperature
Operator: walkup
File: Proton_Minsw_01
INOVA-500 "INOVA500"

Relax. delay 1.500 sec
Pulse 45.0 degrees
Acq. time 3.500 sec
Width 5256.2 Hz
16 repetitions
OBSERVE H1, 500.1967481 MHz
DATA PROCESSING
Line broadening 0.3 Hz
FT size 65536
Total time 1 min, 20 sec

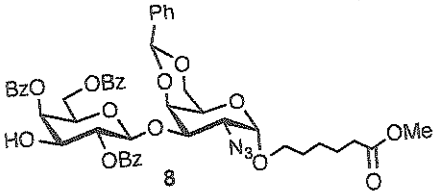

8

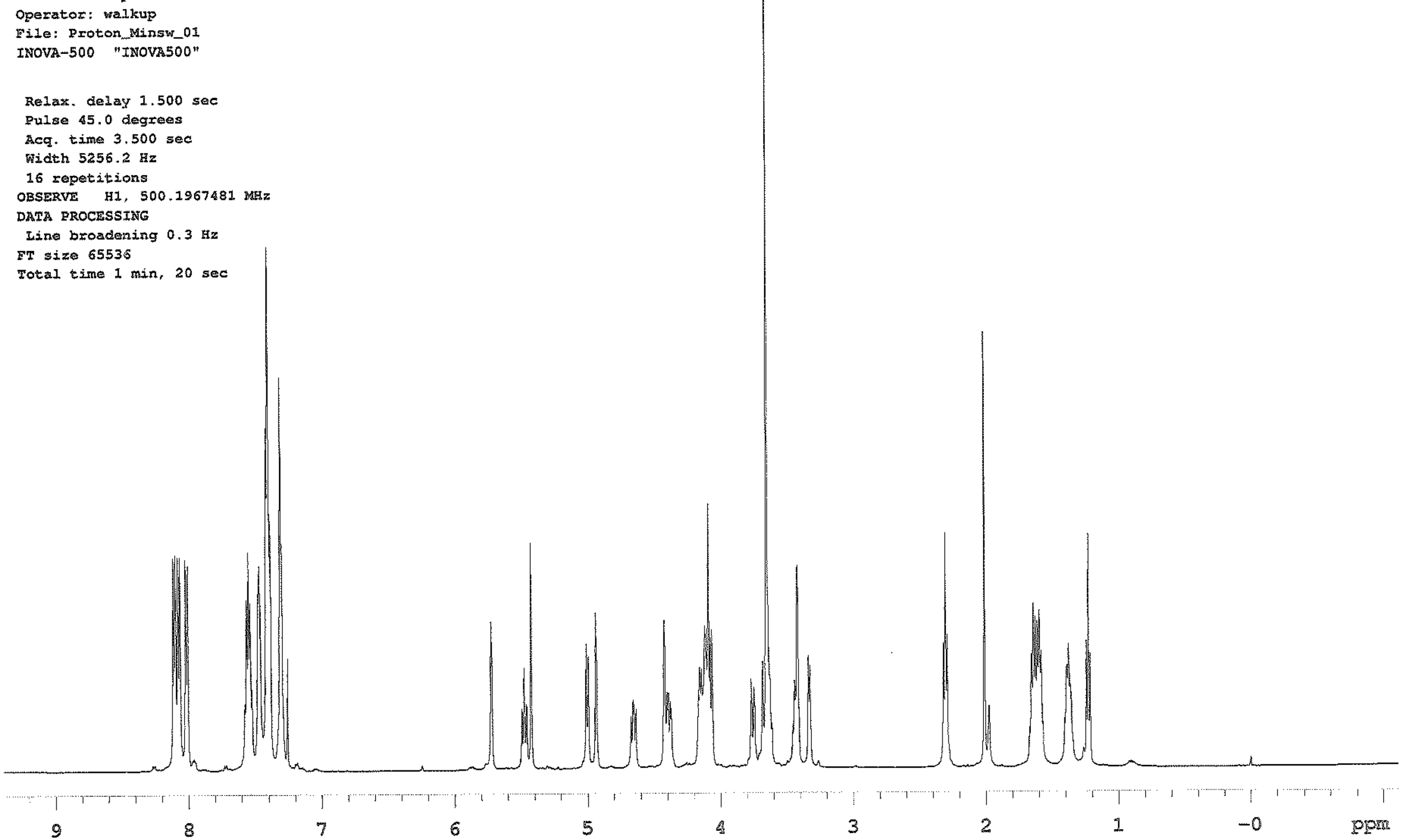

Sample ID: s_20110606_01
File: s_20110606_01/data/Carbon_01.fid

Pulse Sequence: s2pul

Solvent: cdcl3
Ambient temperature
Operator: walkup
File: Carbon_01
INOVA-500 "INOVA500"

Relax. delay 0.700 sec
Pulse 45.0 degrees
Acq. time 1.300 sec
Width 30188.7 Hz
1000 repetitions
OBSERVE C13, 125.7745762 MHz
DECOUPLE H1, 500.1992491 MHz
Power 41 dB
continuously on
WALTZ-16 modulated
DATA PROCESSING
Line broadening 1.0 Hz
FT size 131072
Total time 33 min, 29 sec

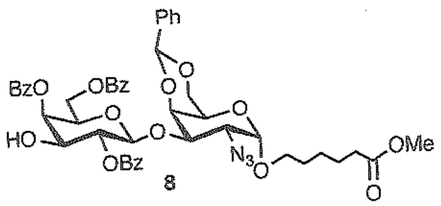

8

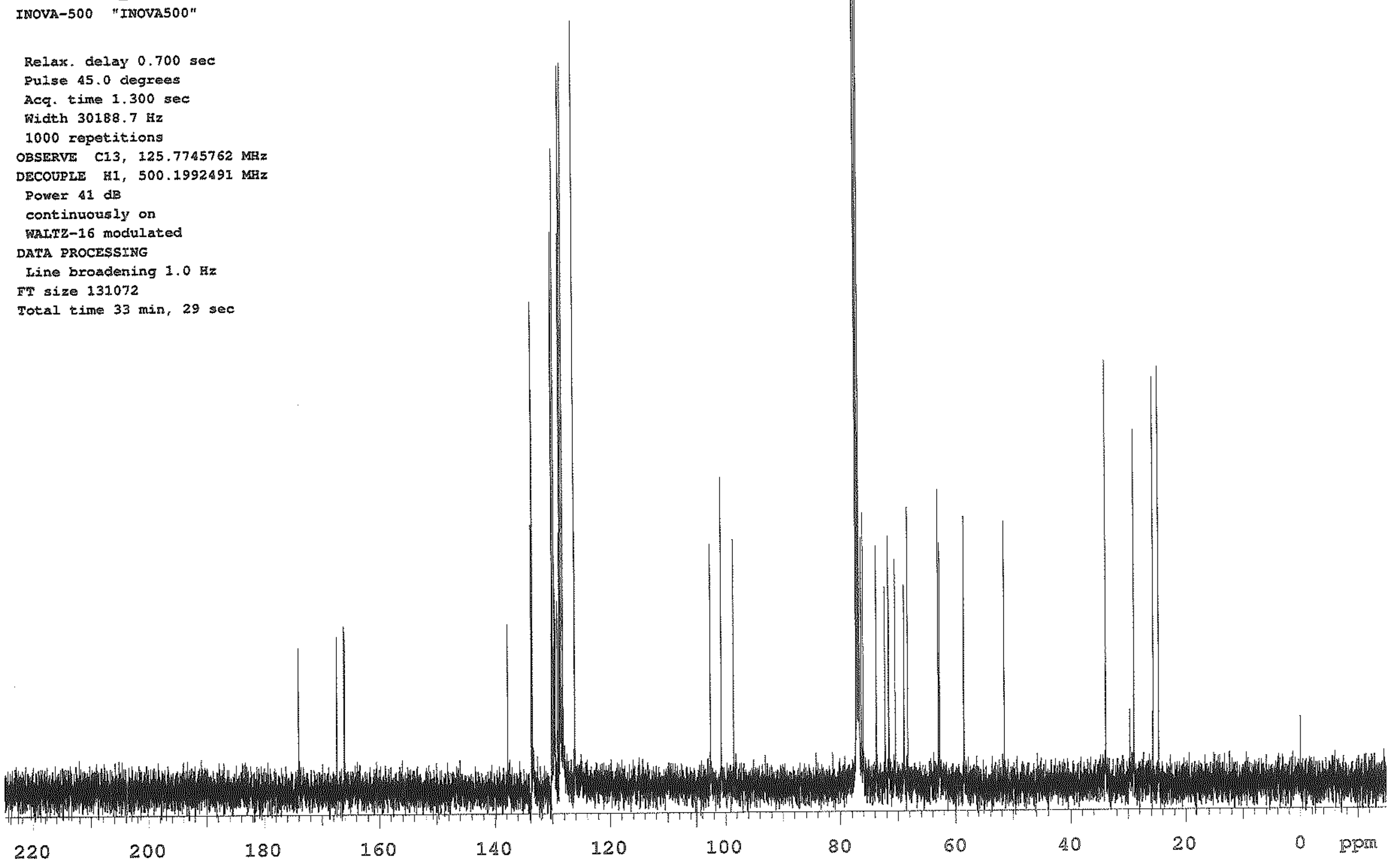

10

Sample ID: s_20111017_01
File: s_20111017_01/data/Proton_Minsw_01.fid

Pulse Sequence: s2pul

Solvent: cdcl3
Ambient temperature
Operator: walkup
File: Proton_Minsw_01
VNMRS-400 "400MR"

Relax. delay 1.500 sec
Pulse 45.0 degrees
Acq. time 3.500 sec
Width 4166.7 Hz
16 repetitions
OBSERVE H1, 399.8639847 MHz
DATA PROCESSING
Line broadening 0.2 Hz
FT size 32768
Total time 1 min, 20 sec

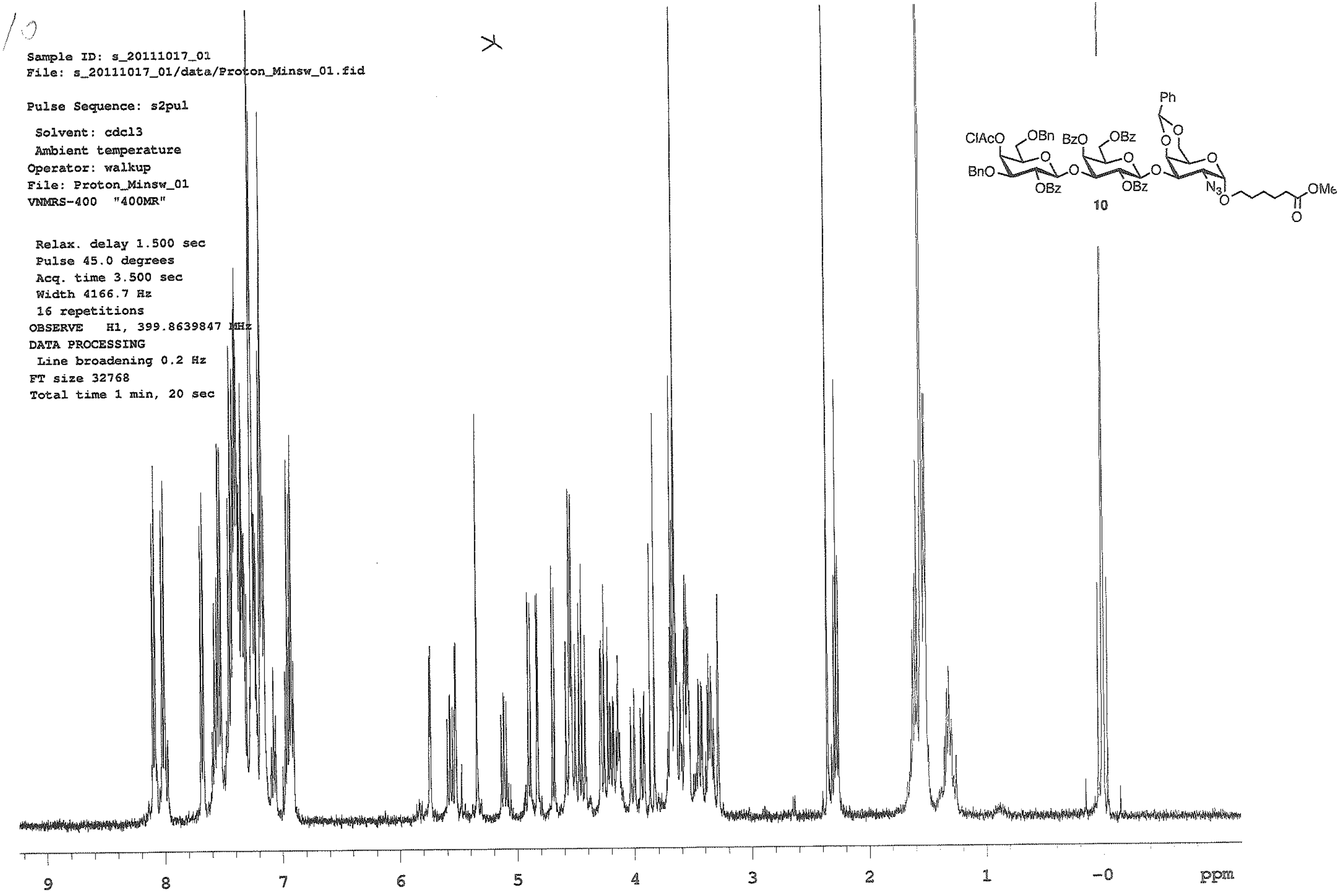

Sample ID: s_20110707_01
File: s_20110707_01/data/Carbon_01.fid

Pulse Sequence: s2pul

Solvent: cdcl3
Ambient temperature
Operator: walkup
File: Carbon_01
INOVA-500 "INOVA500"

Relax. delay 0.700 sec
Pulse 45.0 degrees
Acq. time 1.300 sec
Width 30188.7 Hz
10000 repetitions
OBSERVE C13, 125.7745772 MHz
DECOUPLE H1, 500.1992491 MHz
Power 41 dB
continuously on
WALTZ-16 modulated
DATA PROCESSING
Line broadening 1.0 Hz
FT size 131072
Total time 5 hr, 34 min, 48 sec

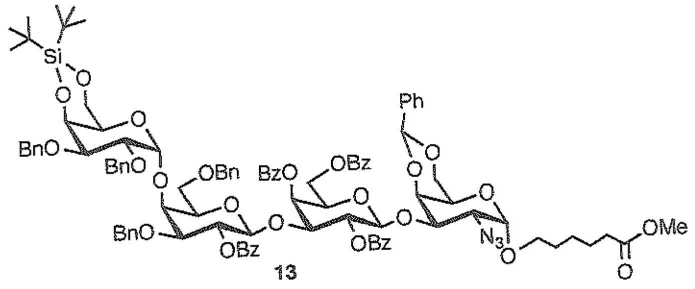

13

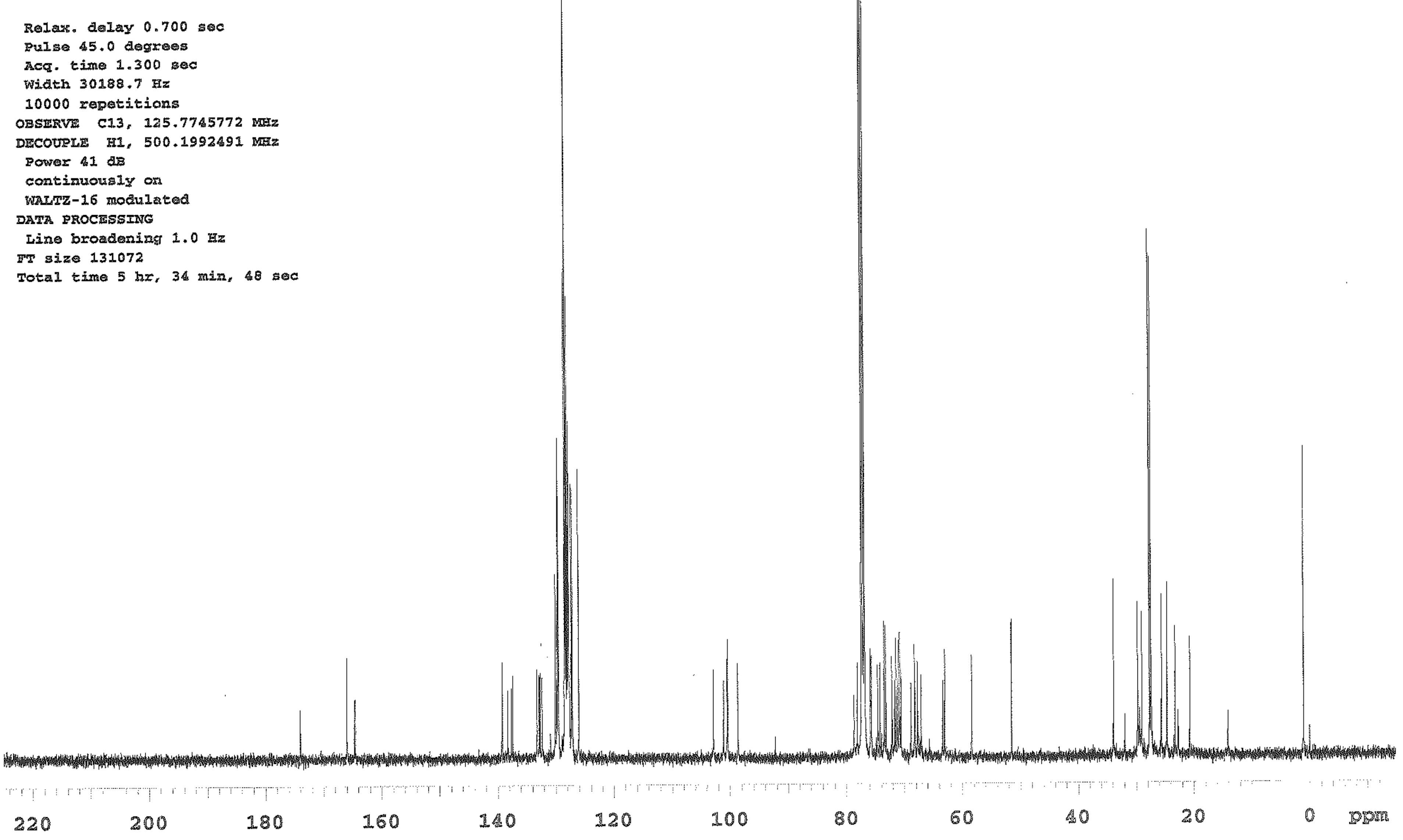

14

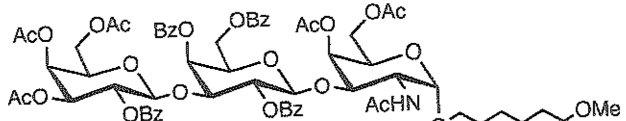

14

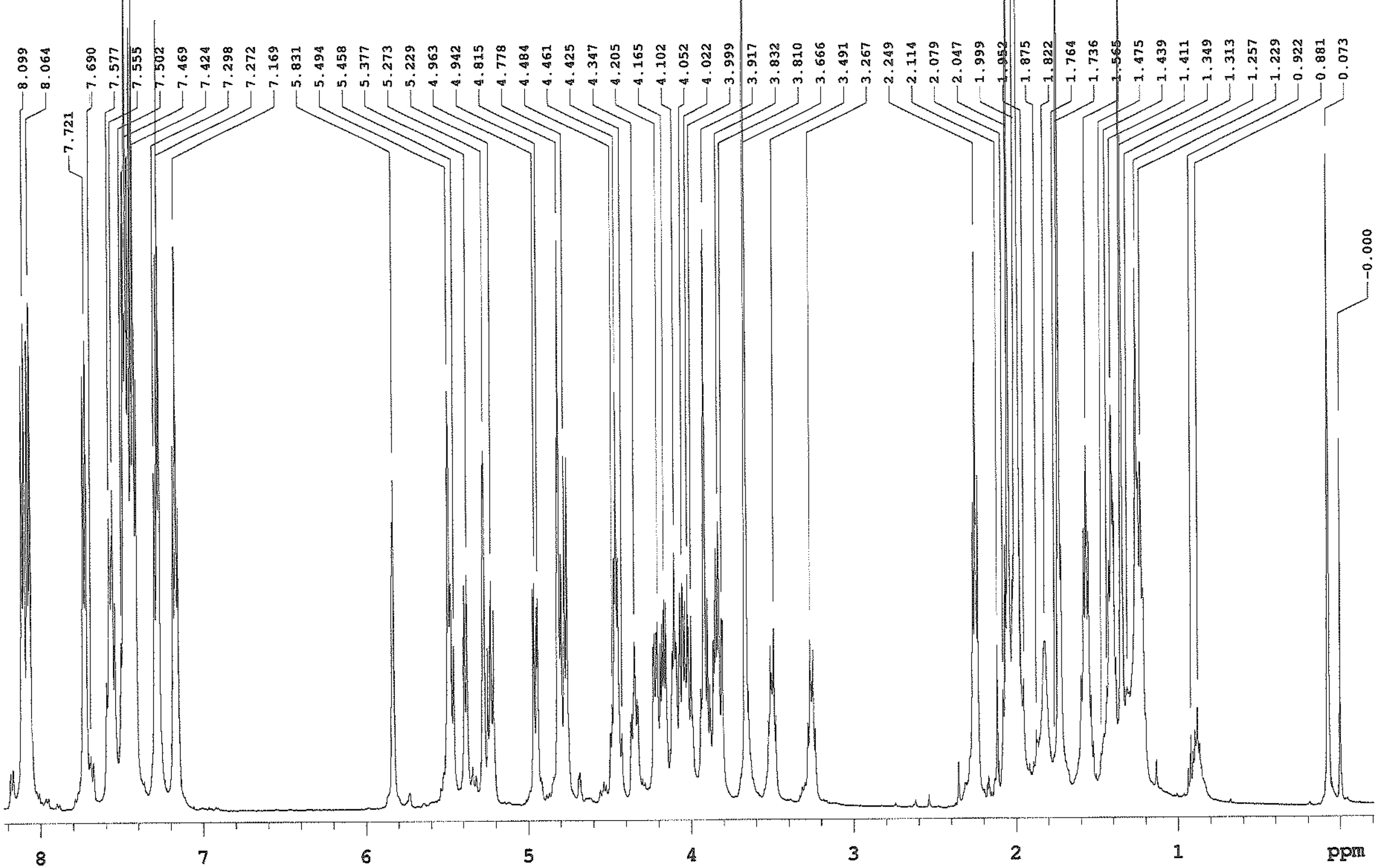
8.099
8.064
7.721
7.690
7.577
7.555
7.502
7.469
7.424
7.298
7.272
7.169
5.831
5.494
5.458
5.377
5.273
5.229
4.963
4.942
4.815
4.778
4.484
4.461
4.425
4.347
4.205
4.165
4.102
4.052
4.022
3.999
3.917
3.832
3.810
3.666
3.491
3.267
2.249
2.114
2.079
2.047
1.999
1.952
1.875
1.822
1.764
1.736
1.565
1.475
1.439
1.411
1.349
1.313
1.257
1.229
0.922
0.881
0.073
-0.000
8
7
6
5
4
3
2
1
ppm

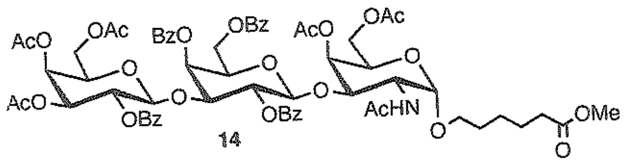
AcO
OAc
BzO
OBz
AcO
OAc
AcO
OBz
OBz
AcHN
OMe

14

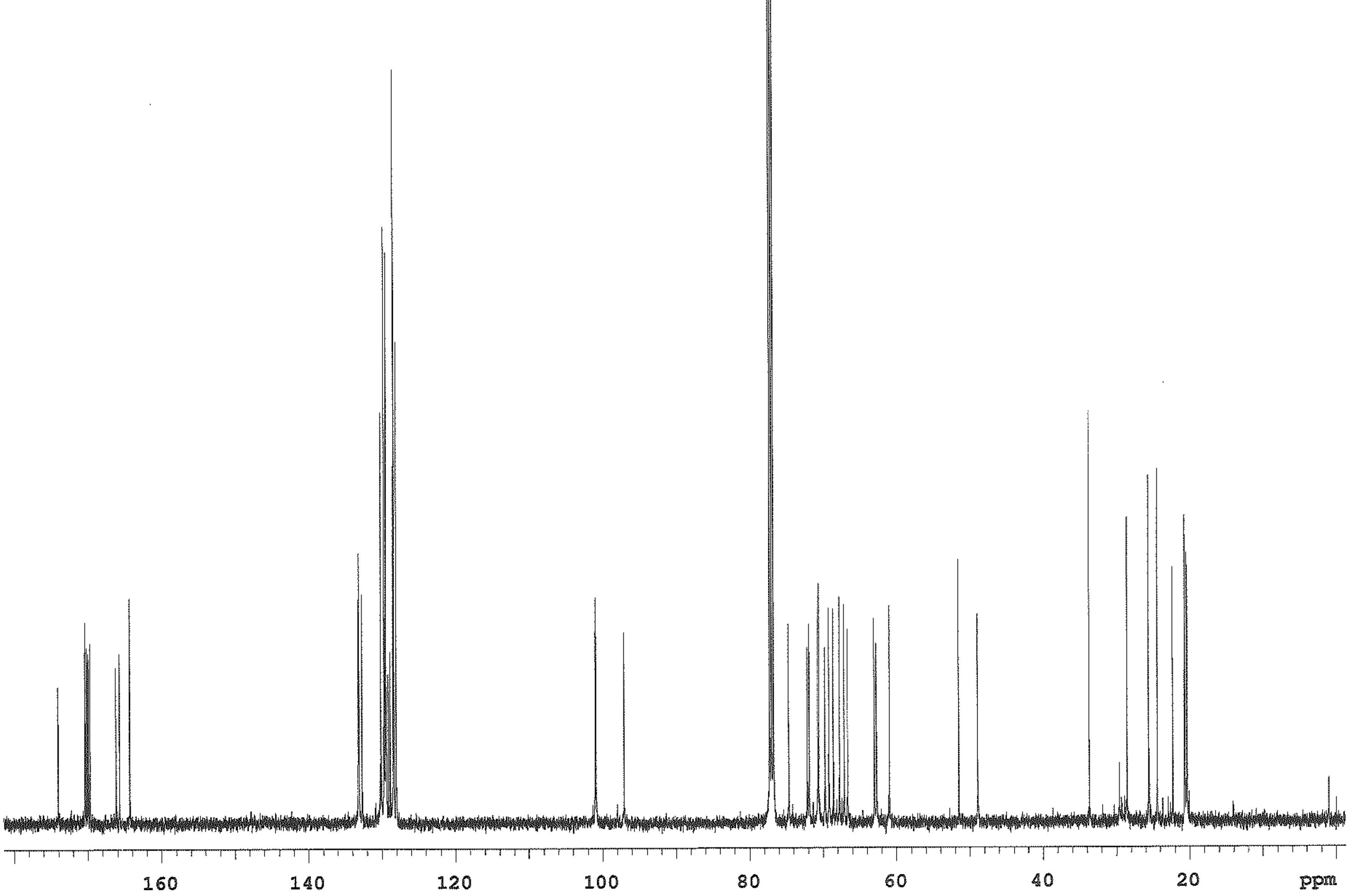
160
140
120
100
80
60
40
20
ppm

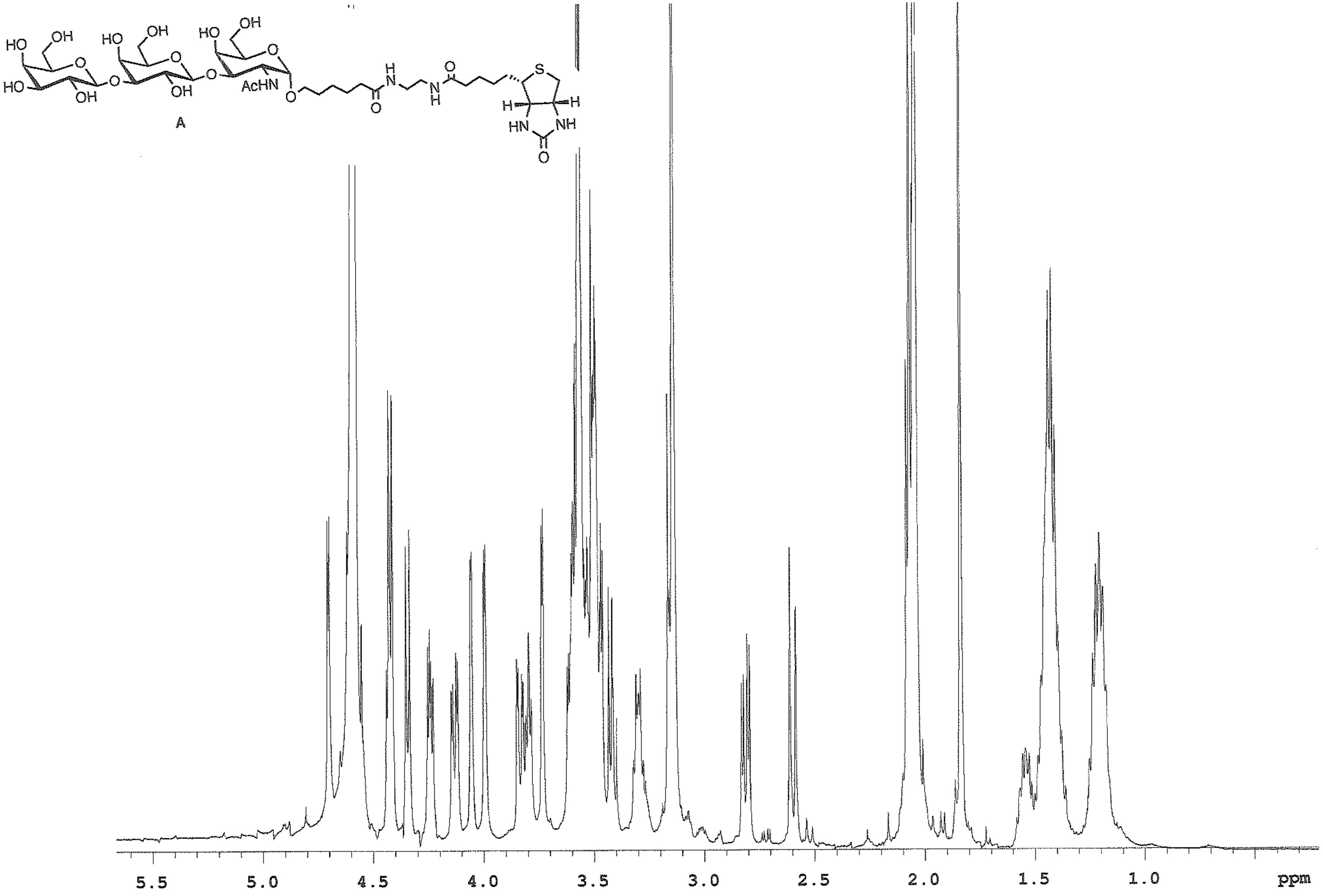

A

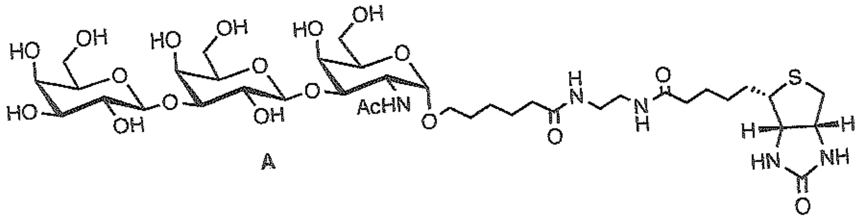
HO
OH
AcHN
O
H
N
S
HN
NH

A

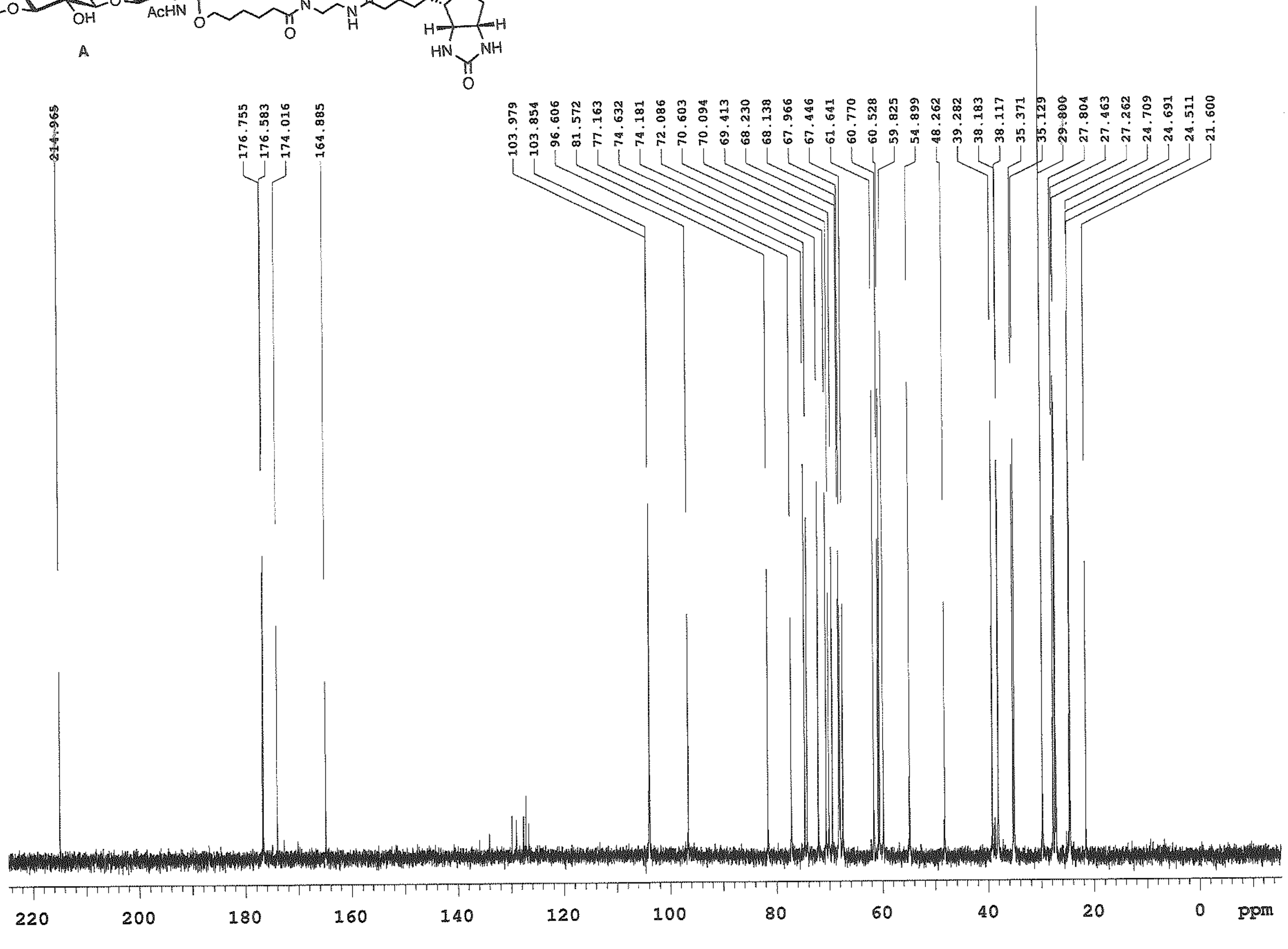
214.965
176.755
176.583
174.016
164.885
103.979
103.854
96.606
81.572
77.163
74.632
74.181
72.086
70.603
70.094
69.413
68.230
68.138
67.966
67.446
61.641
60.770
60.528
59.825
54.899
48.262
39.282
38.183
38.117
35.371
35.129
29.800
27.804
27.463
27.262
24.709
24.691
24.511
21.600
220
200
180
160
140
120
100
80
60
40
20
0
ppm

Sample ID: s_20111031_01
File: s_20111031_01/data/Proton_

Pulse Sequence: s2pul

Solvent: cdcl3
Ambient temperature
Operator: walkup
File: Proton_Minsw_01
INOVA-500 "INOVA500"

Relax. delay 1.500 sec
Pulse 45.0 degrees
Acq. time 3.500 sec
Width 5183.4 Hz
16 repetitions
OBSERVE H1, 500.1967467 MHz
DATA PROCESSING
Line broadening 0.3 Hz
FT size 65536
Total time 1 min, 20 sec

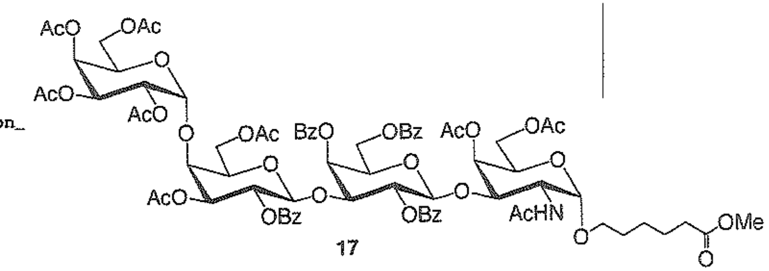

17

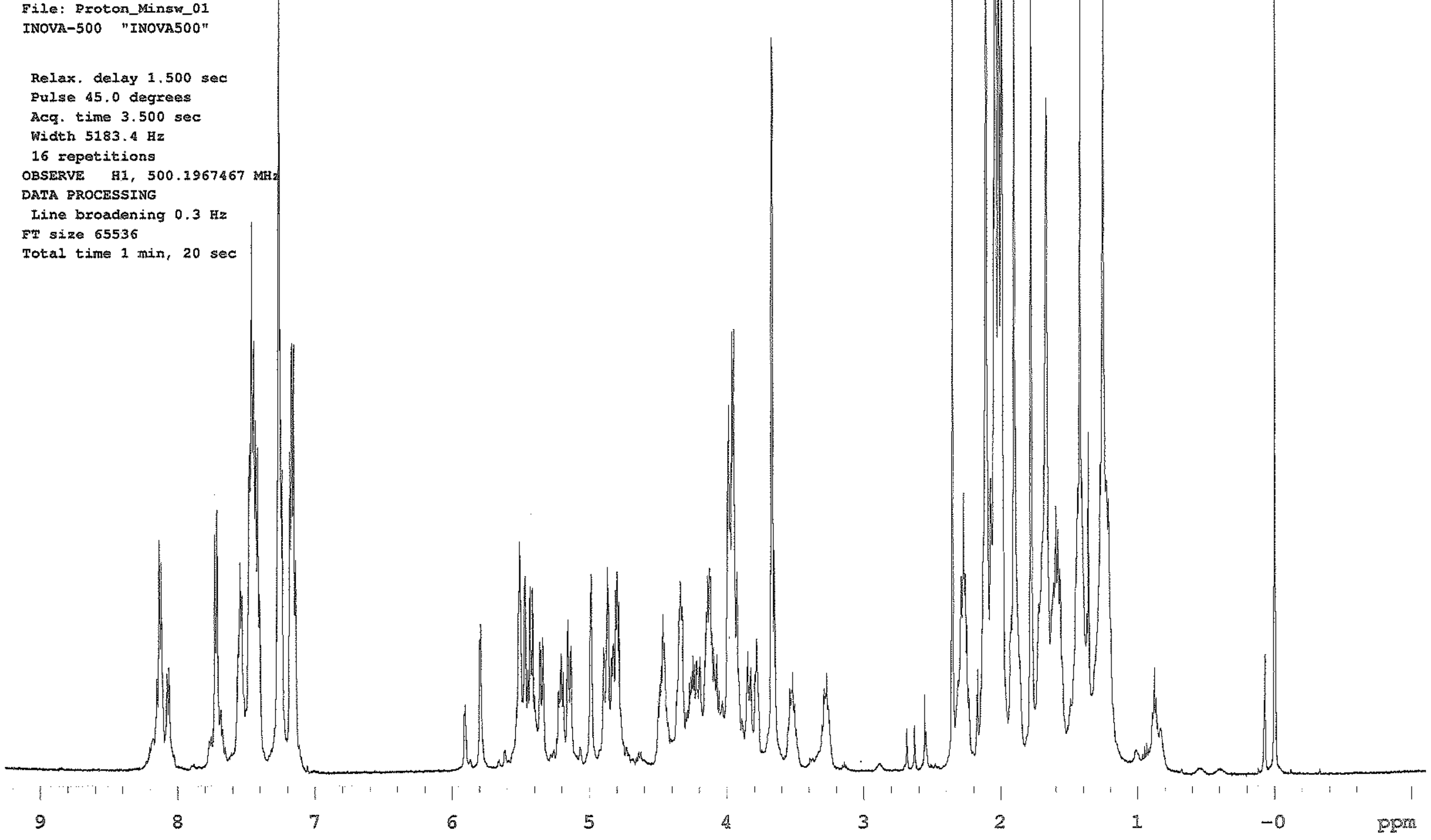

Sample ID: s_20111031_0
File: s_20111031_01/data

Pulse Sequence: s2pul

Solvent: cdcl3
Ambient temperature
Operator: walkup
File: Carbon_01
INOVA-500 "INOVA500"

Relax. delay 0.700 sec
Pulse 45.0 degrees
Acq. time 1.300 sec
Width 30188.7 Hz
6000 repetitions
OBSERVE C13, 125.7745727 MHz
DECOUPLE H1, 500.1992491 MHz
Power 41 dB
continuously on
WALTZ-16 modulated
DATA PROCESSING
Line broadening 1.0 Hz
FT size 131072
Total time 3 hr, 20 min, 53 sec

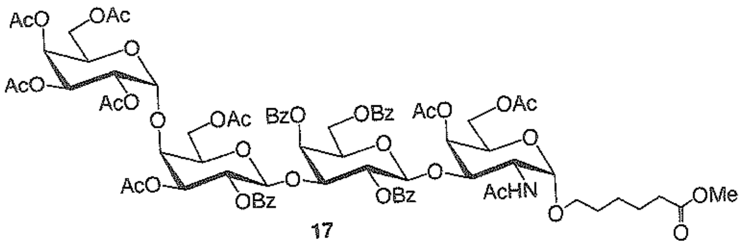

17

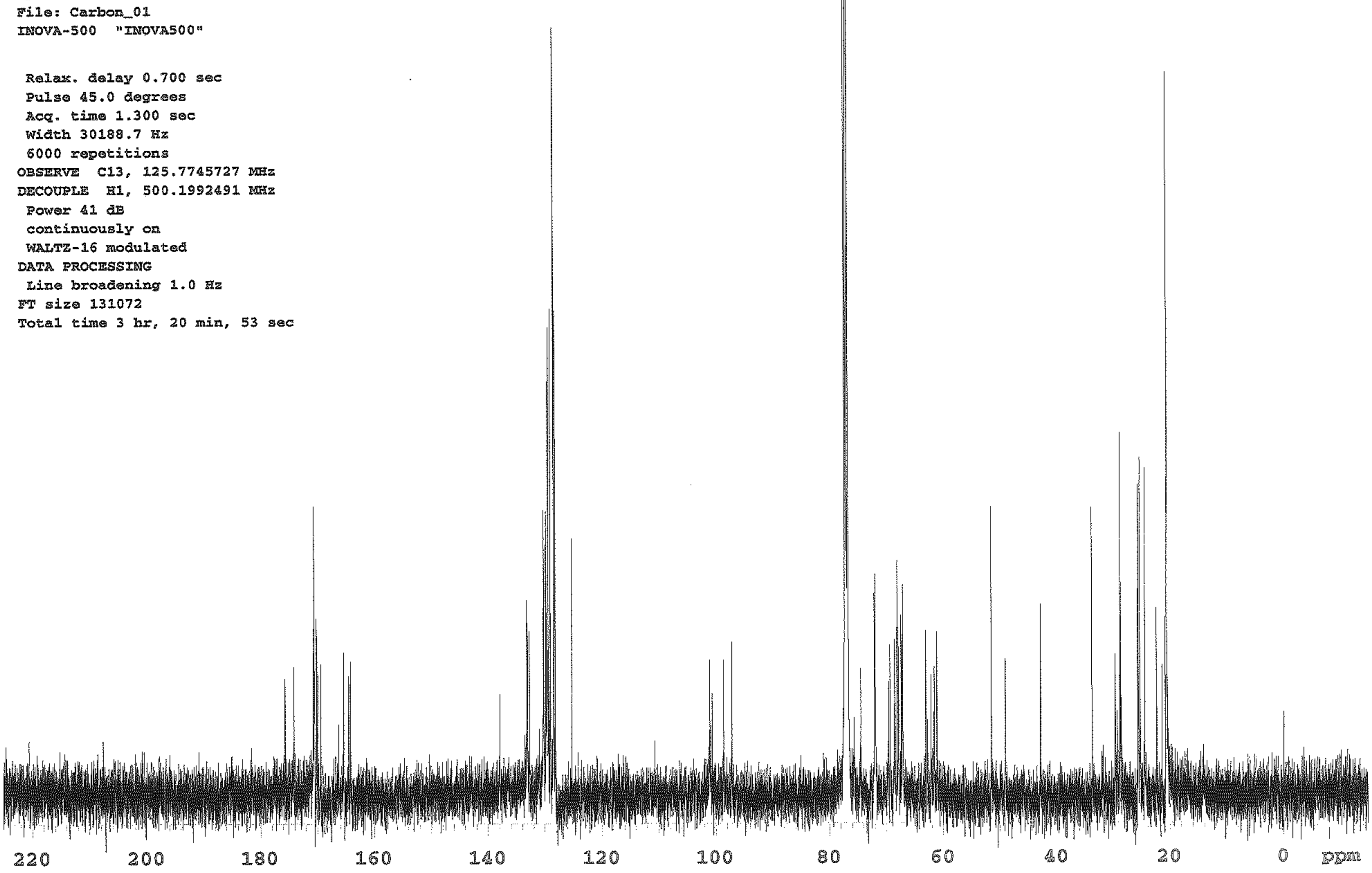

Sample ID: s_2[illegible]
File: s_20111[illegible]

Pulse Sequen[illegible]

Solvent: cd3o[illegible]
Temp. 40.0 C / 313.1 [illegible]
Operator: walkup
File: Proton_01
INOVA-500 "INOVA500"

Relax. delay 1.500 sec
Pulse 45.0 degrees
Acq. time 3.500 sec
Width 8003.2 Hz
32 repetitions
OBSERVE H1, 500.1987172 MHz
DATA PROCESSING
Line broadening 0.3 Hz
FT size 65536
Total time 2 min, 40 sec

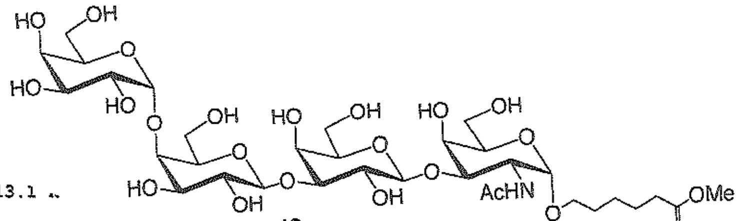

18

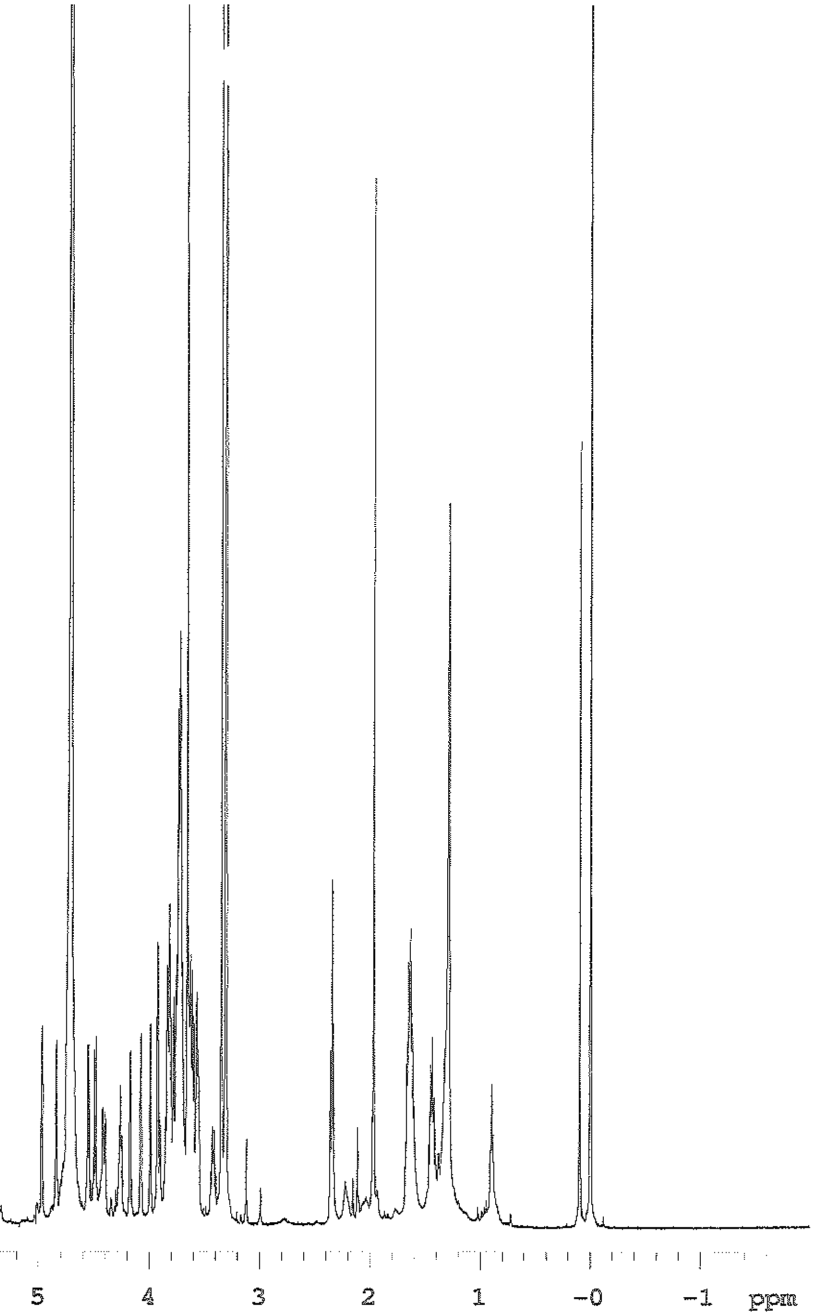

Sample ID: s_20111102_01
File: s_20111102_01/data/Carbon_01.fid

Pulse Sequence: s2pul

Solvent: cd3od
Ambient temperature
Operator: walkup
File: Carbon_01
INOVA-500 "INOVA500"

Relax. delay 0.700 sec
Pulse 45.0 degrees
Acq. time 1.300 sec
Width 30188.7 Hz
24000 repetitions
OBSERVE C13, 125.7748889 MHz
DECOUPLE H1, 500.2012199 MHz
Power 41 dB
continuously on
WALTZ-16 modulated
DATA PROCESSING
Line broadening 1.0 Hz
FT size 131072
Total time 13 hr, 23 min, 32 sec

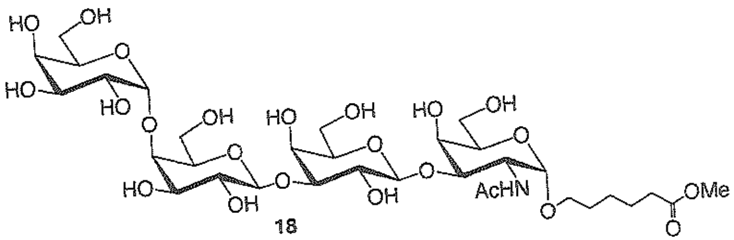

18

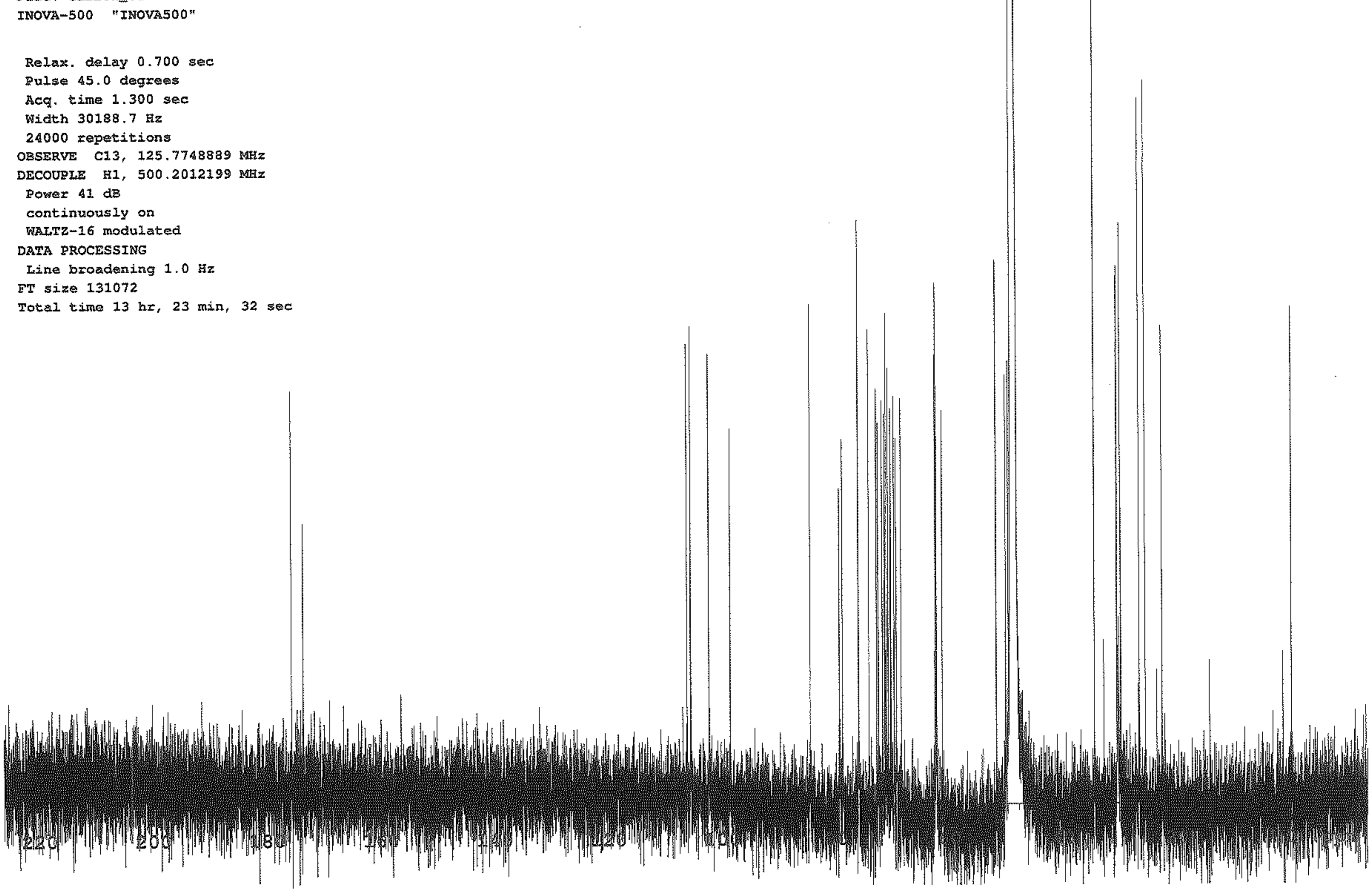

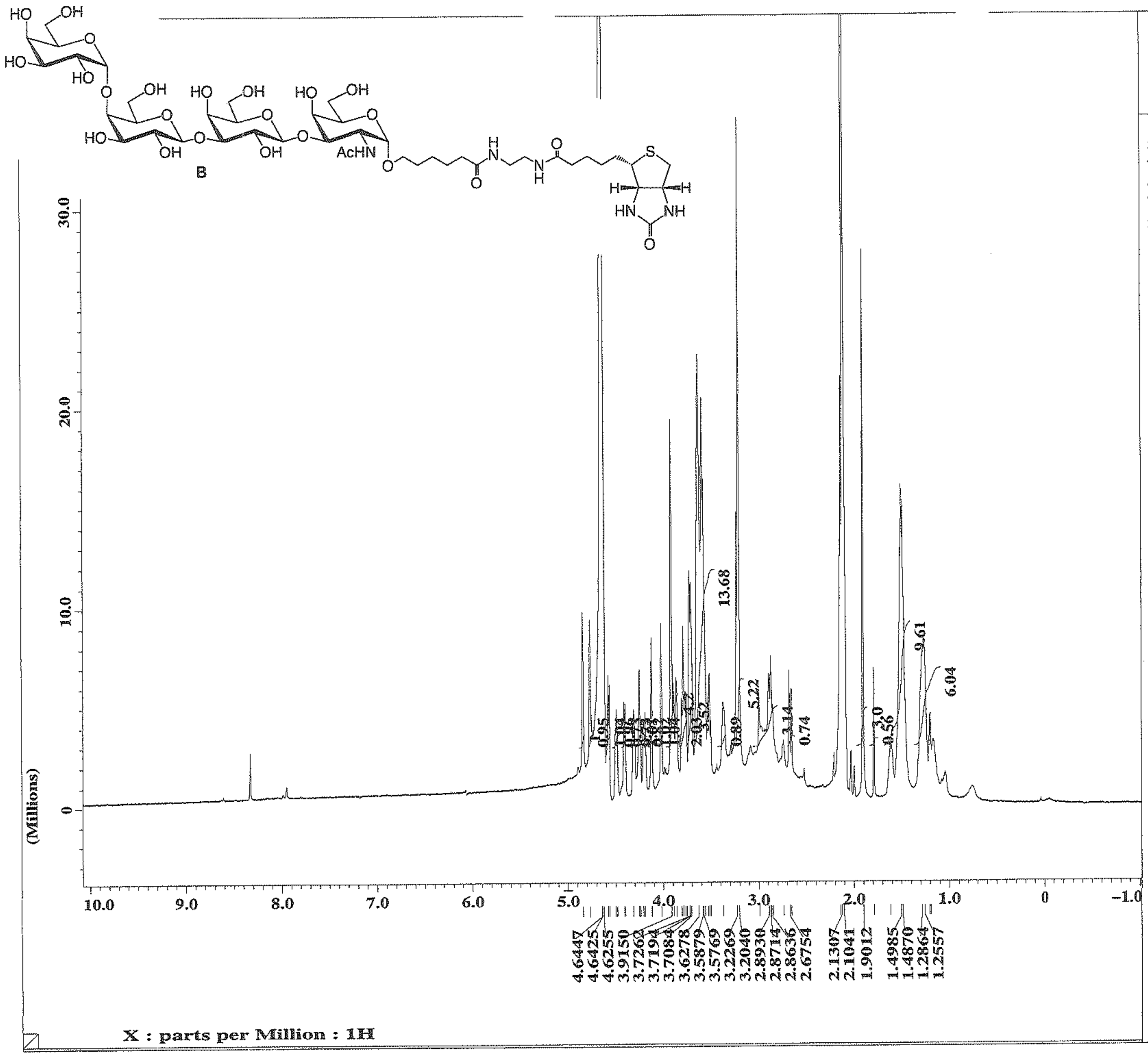

JEOL

---- ACQUISITION PARAMETERS ----

File Name	= TNNO111120-H.10
Author	= JEOL LTD.
Sample ID	= ohgane
Content	= Single Pulse Experim
Creation Date	= 20-NOV-2011 07:31:58
Revision Date	= 21-NOV-2011 12:04:28
Spec Site	= ECP600
Spec Type	= DELTA_NMR
Data Format	= 1D COMPLEX
Dimensions	= X
Dim Title	= 1H
Dim Size	= 32768
Dim Units	= [ppm]
Actual_start_time	= 20-NOV-2011 07:21:59
Delay_of_start	= 1[s]
Digital_filter	= FALSE
End_time	= 20-NOV-2011 07:32:00
Experiment	= single_pulse.exp
Field_strength	= 14.09636928[T]
Filter_mode	= BUTTERWORTH
Filter_width	= 4.50129228[kHz]
Irr_code	= 210
Irr_noise	= WALTZ
Irr_pwidth	= 51[us]
Iterations	= 0
Local_time	= 20-NOV-2011 07:31:57
Obs_noise	= WAUGH
Obs_pwidth	= 1[us]
Probe_id	= 2692
Recvr_gain	= 19
Relaxation_delay	= 1[s]
Scans	= 128
Solvent	= D2O
Spin_get	= 14[Hz]
Spin_lock_90	= 34[us]
Spin_lock_attn	= 10.4[dB]
Temp_get	= 26.1[dC]
X90	= 12[us]
X_acq_duration	= 3.637248[s]
X_angle	= 45[deg]
X_domain	= 1H
X_freq	= 600.17530548[MHz]
X_offset	= 5[ppm]
X_points	= 32768
X_prescans	= 0
X_pulse	= 6[us]
X_resolution	= 0.27494153[Hz]
X_sweep	= 9.00900901[kHz]
Tri90	= 10[us]
Tri_noise	= WALTZ
Tri_pwidth	= 1[us]
Qua90	= 10[us]
Qua_noise	= WAUGH
Qua_pwidth	= 1[us]

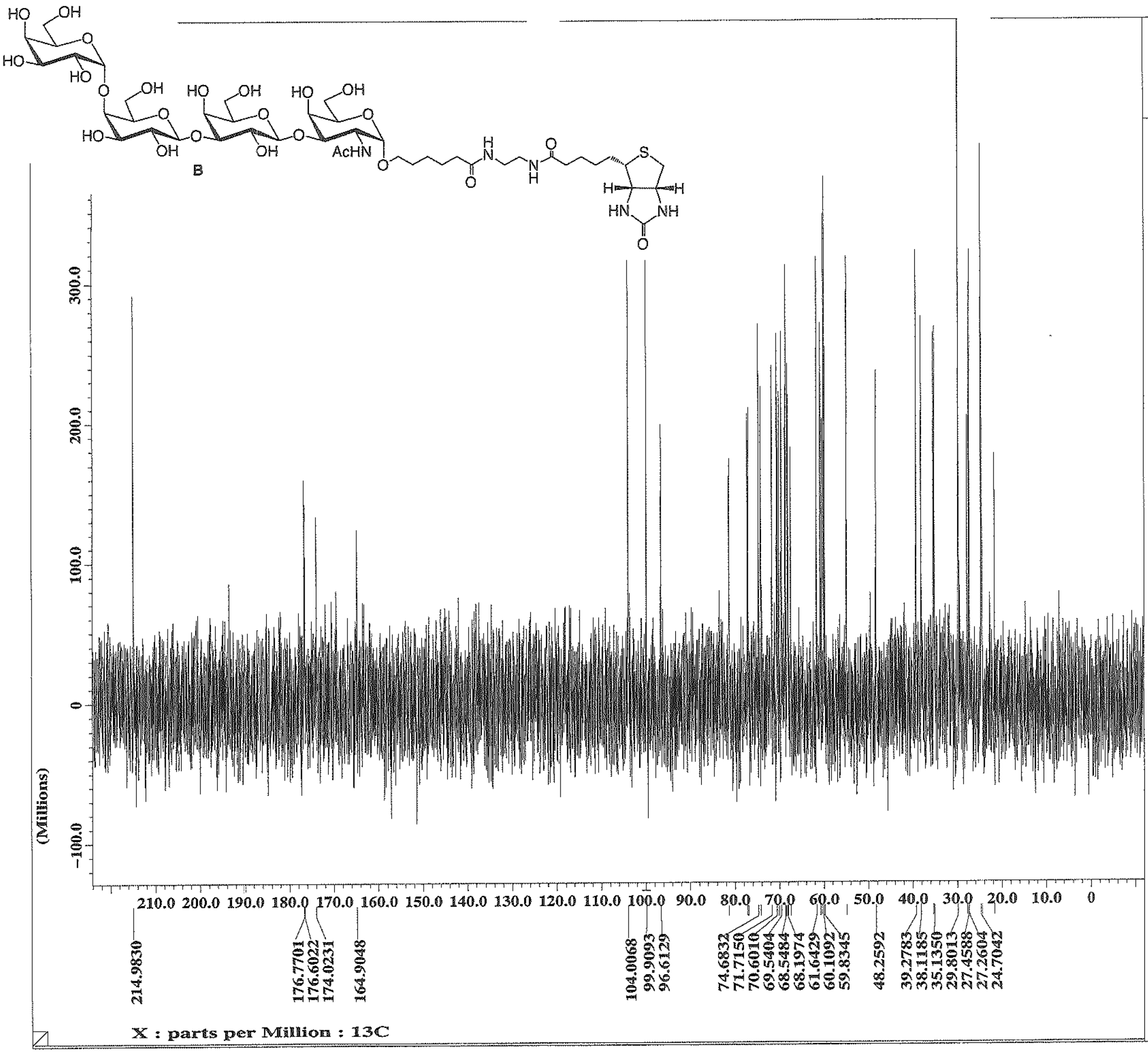

JEOL

---- ACQUISITION PARAMETERS ----
File Name = TNNO111120-C.3
Author = JEOL LTD.
Sample ID = ohgane
Content = Single Pulse with Br
Creation Date = 20-NOV-2011 20:32:44

Revision Date = 21-NOV-2011 12:07:25
Spec Site = ECP600

Spec Type = DELTA_NMR
Data Format = 1D COMPLEX
Dimensions = X
Dim Title = 13C
Dim Size = 32768
Dim Units = [ppm]
Actual_start_time = 20-NOV-2011 07:34:00
Delay_of_start = 1[s]
Digital_filter = FALSE
End_time = 20-NOV-2011 20:32:49
Experiment = single_pulse_dec
Field_strength = 14.09636928[T]
Filter_mode = BUTTERWORTH
Filter_width = 18.8641788[kHz]
Irr_code = 210
Irr_domain = 1H
Irr_freq = 600.17530548[MHz]
Irr_noise = WALTZ
Irr_offset = 5.0[ppm]
Irr_pwidth = 50[us]
Iterations = 0
Local_time = 20-NOV-2011 20:32:44
Obs_noise = WAUGH
Obs_pwidth = 1[us]
Probe_id = 2692
Recvr_gain = 30
Relaxation_delay = 1[s]
Scans = 25000
Solvent = D2O
Spin_get = 14[Hz]
Spin_lock_90 = 34[us]
Spin_lock_attn = 10.4[dB]
Temp_get = 26.5[dC]
X90 = 12.3[us]
X_acq_duration = 0.868352[s]
X_angle = 30[deg]
X_domain = 13C
X_freq = 150.92852325[MHz]
X_offset = 100[ppm]
X_points = 32768
X_prescans = 4
X_pulse = 4.1[us]
X_resolution = 1.15164187[Hz]
X_sweep = 37.73584906[kHz]
Tri90 = 10[us]
Tri_noise = WALTZ
Tri_pwidth = 1[us]
Qua90 = 10[us]
Qua_noise = WAUGH
Qua_pwidth = 1[us]

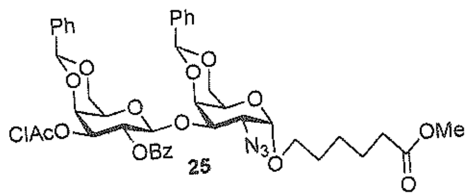

25

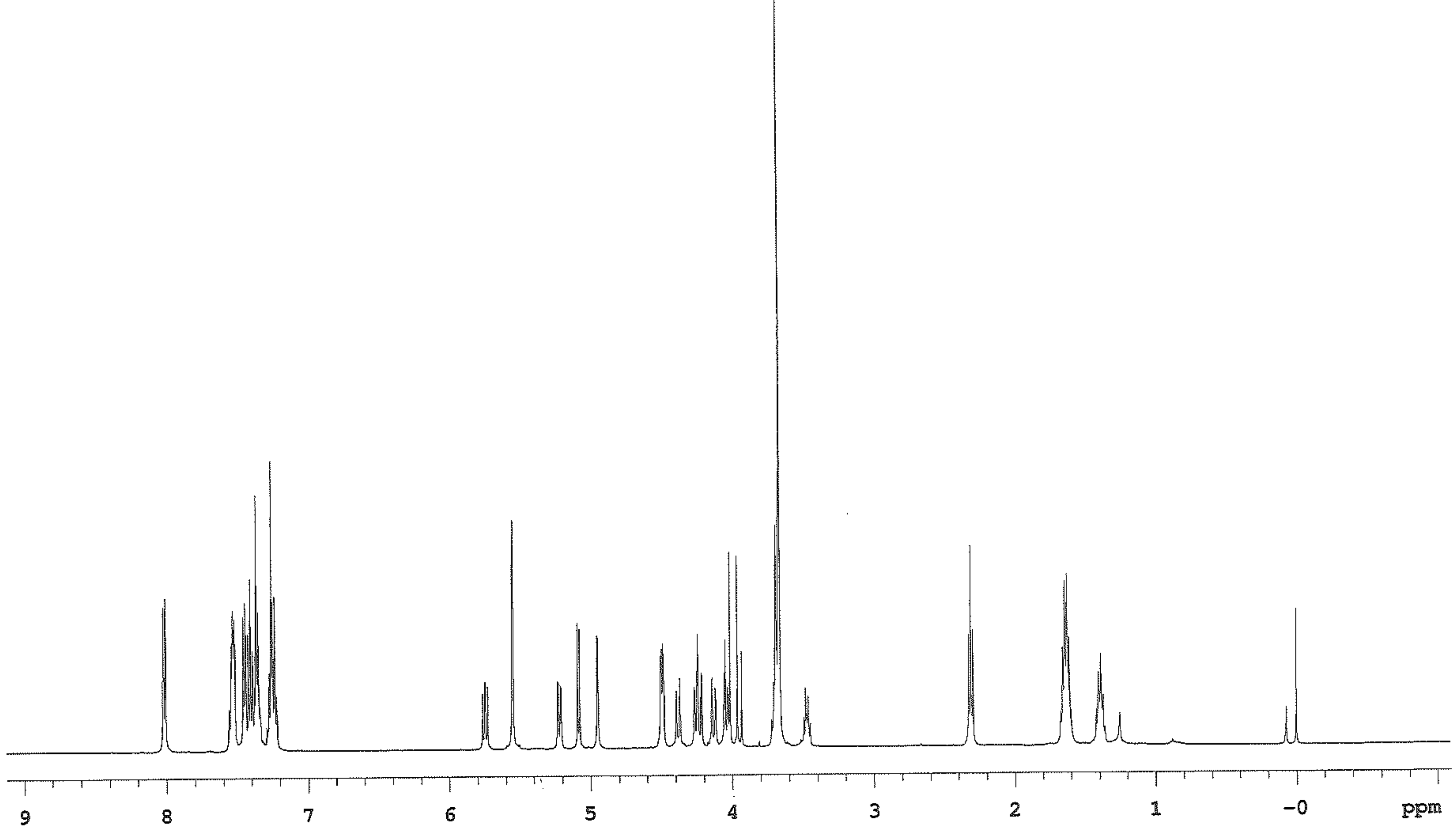

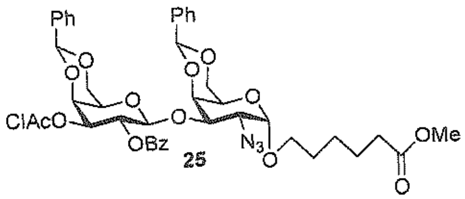

Ph
Ph
ClAcO
OBz
N3
OMe

25

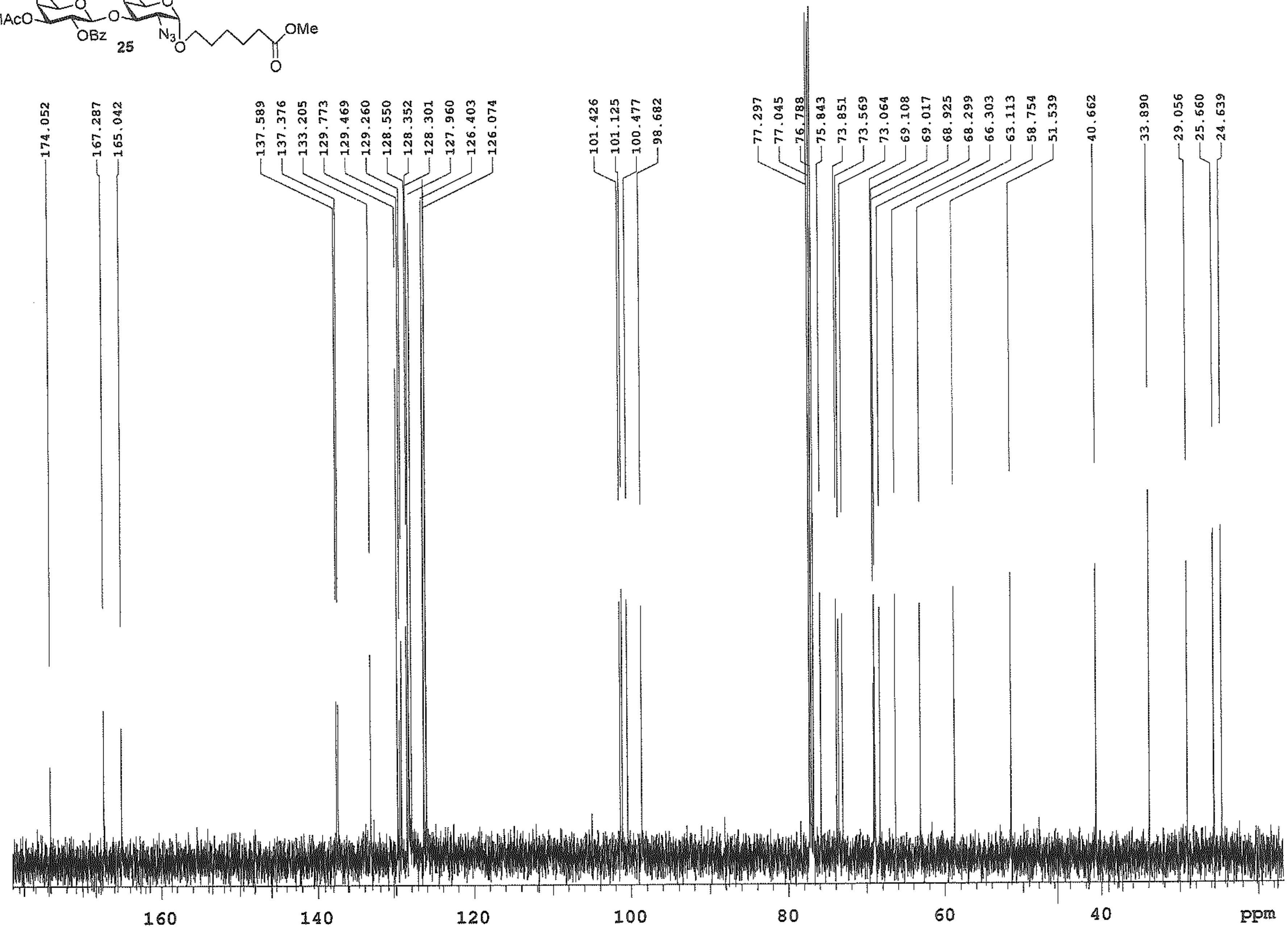

174.052
167.287
165.042
137.589
137.376
133.205
129.773
129.469
129.260
128.550
128.352
128.301
127.960
126.403
126.074
101.426
101.125
100.477
98.682
77.297
77.045
76.788
75.843
73.851
73.569
73.064
69.108
69.017
68.925
68.299
66.303
63.113
58.754
51.539
40.662
33.890
29.056
25.660
24.639
160
140
120
100
80
60
40
ppm

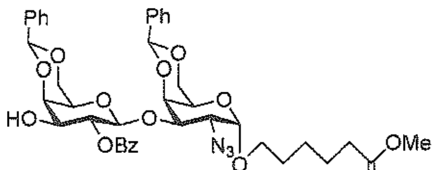

26

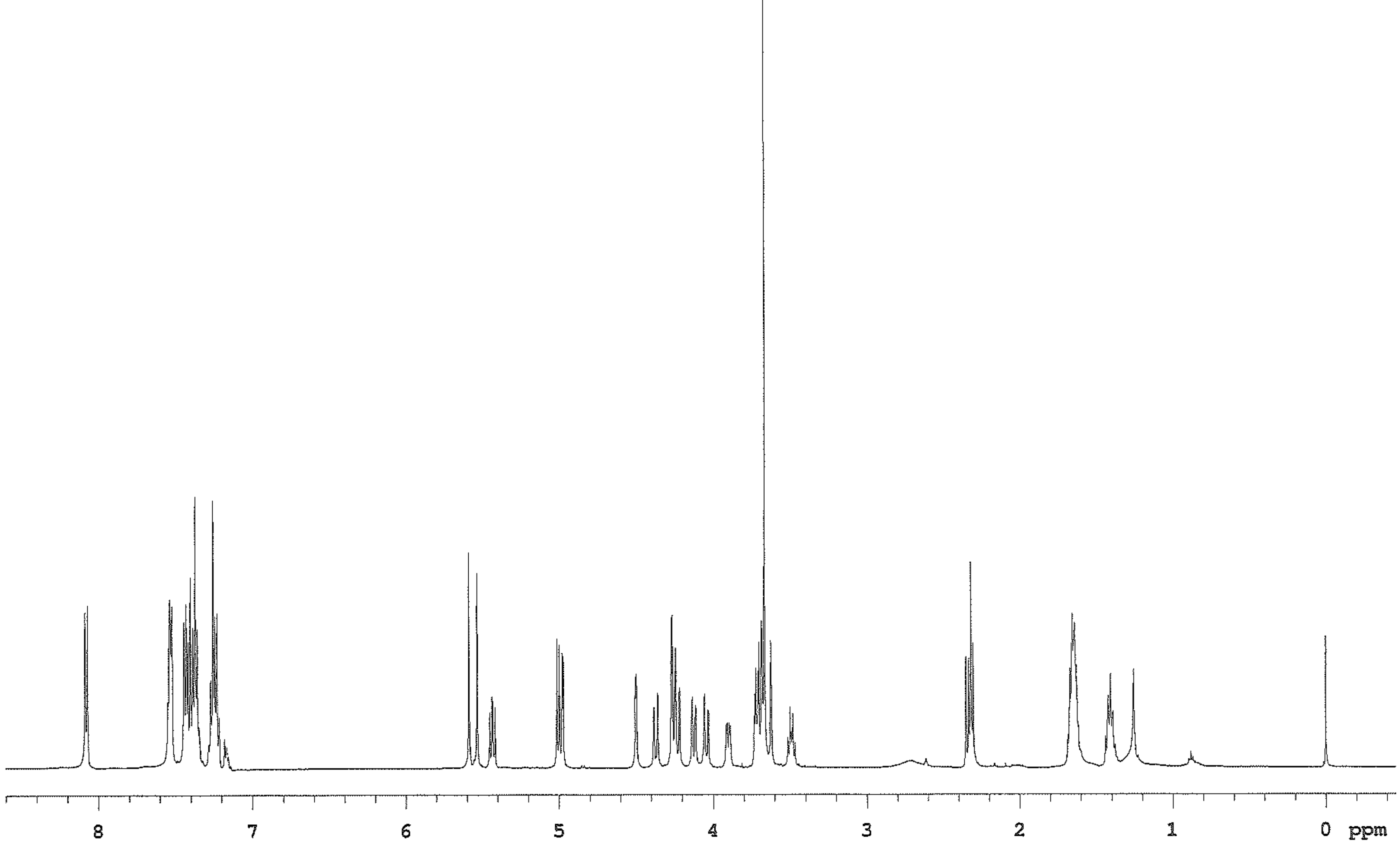

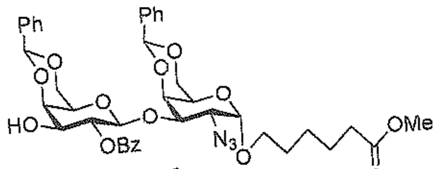

26

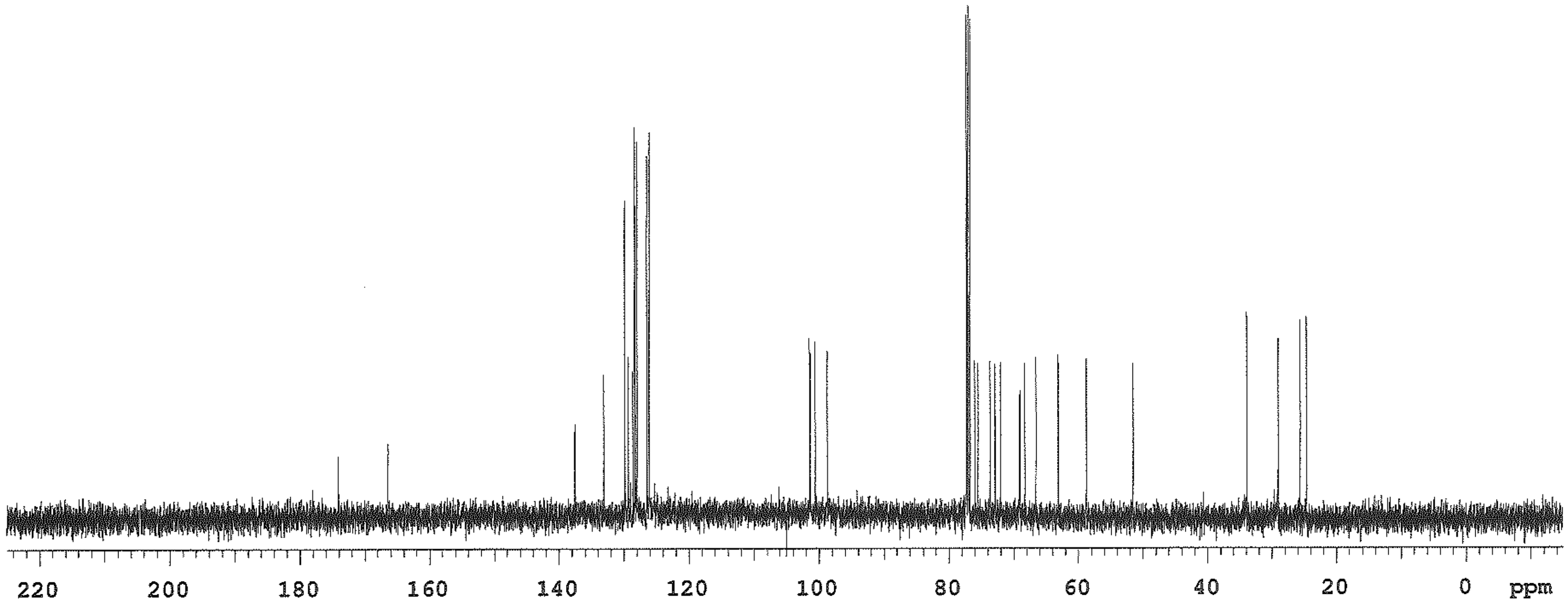

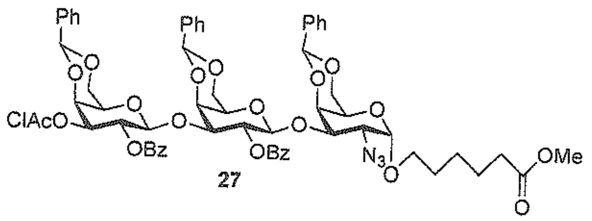

27

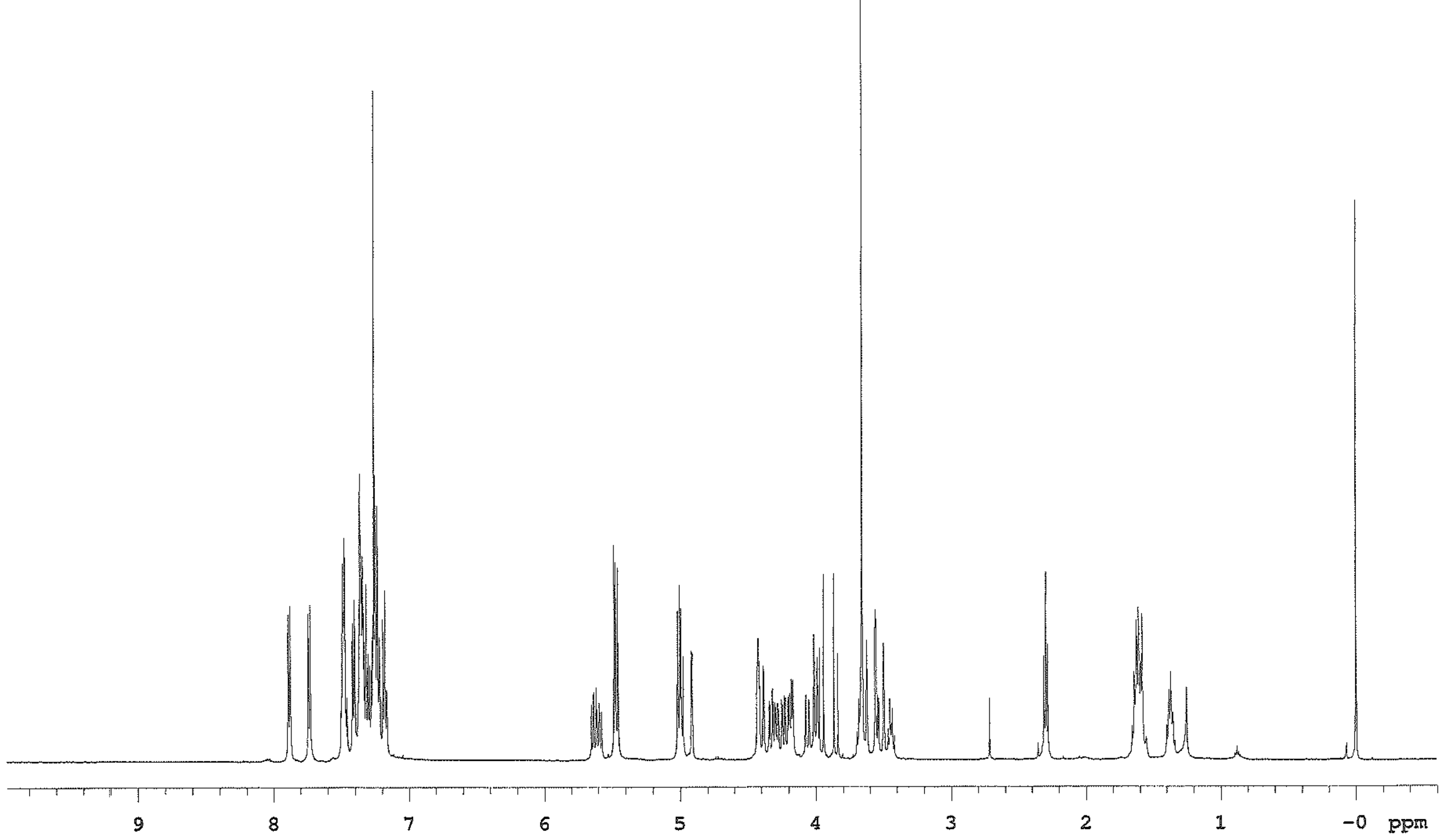

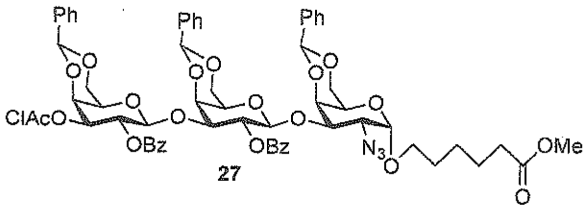
Ph
Ph
Ph
ClAcO
OBz
OBz
N3
OMe
O

27

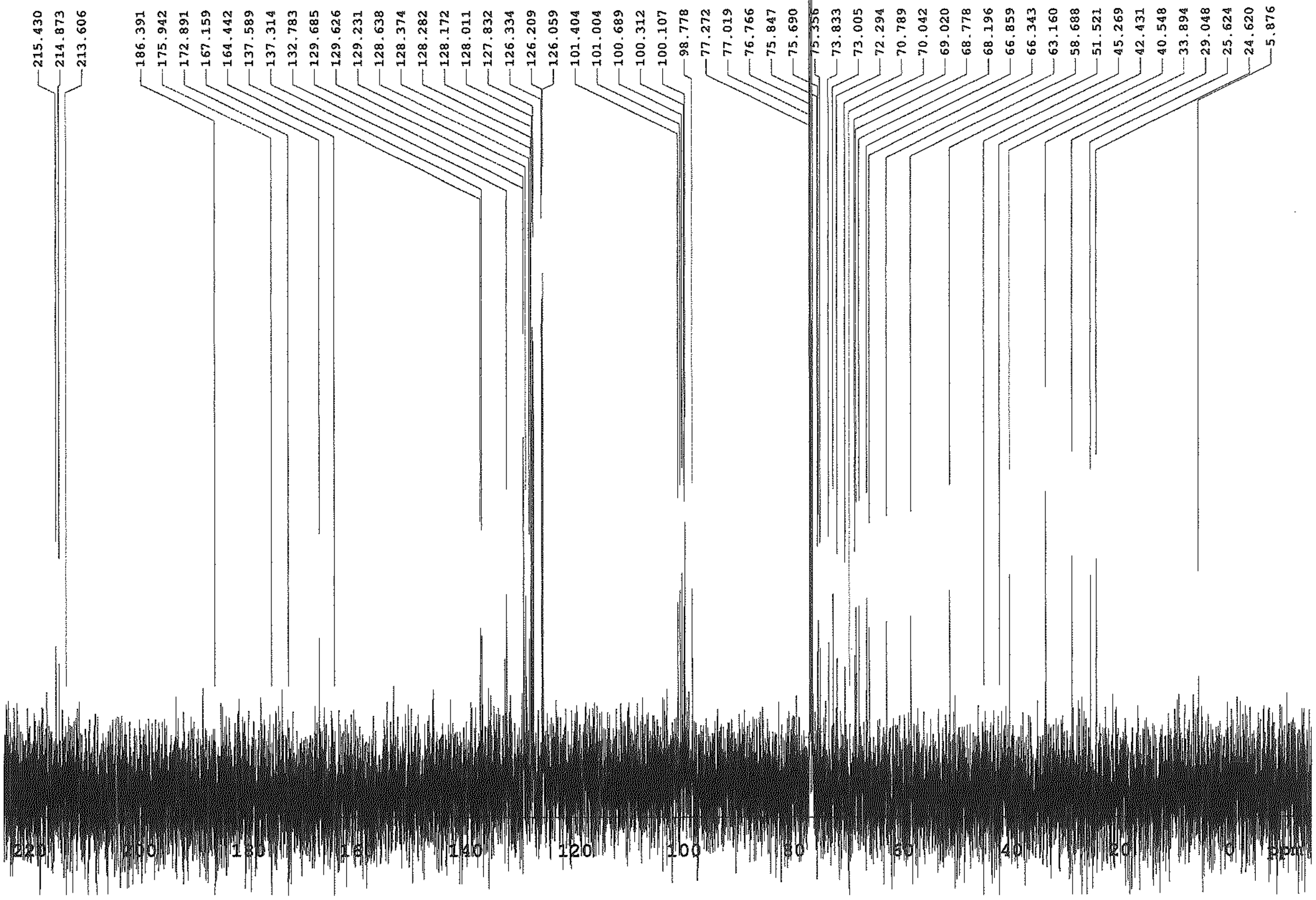
215.430
214.873
213.606
186.391
175.942
172.891
167.159
164.442
137.589
137.314
132.783
129.685
129.626
129.231
128.638
128.374
128.282
128.172
128.011
127.832
126.334
126.209
126.059
101.404
101.004
100.689
100.312
100.107
98.778
77.272
77.019
76.766
75.847
75.690
75.356
73.833
73.005
72.294
70.789
70.042
69.020
68.778
68.196
66.859
66.343
63.160
58.688
51.521
45.269
42.431
40.548
33.894
29.048
25.624
24.620
5.876
120
100
80
60
40
20
0
ppm

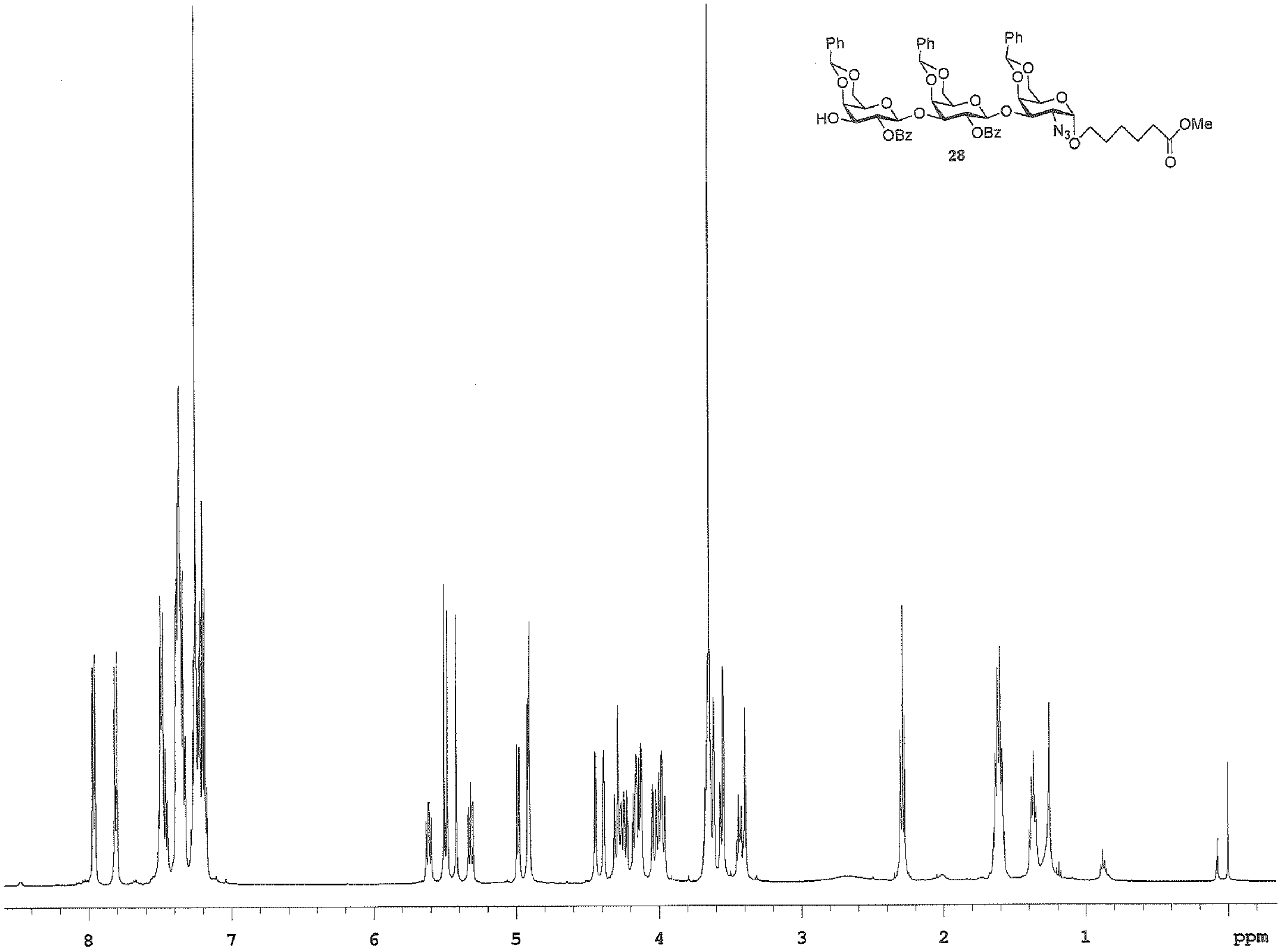
28

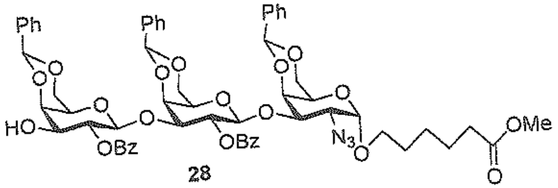
Ph
Ph
Ph
HO
OBz
OBz
N3
OMe
O

28

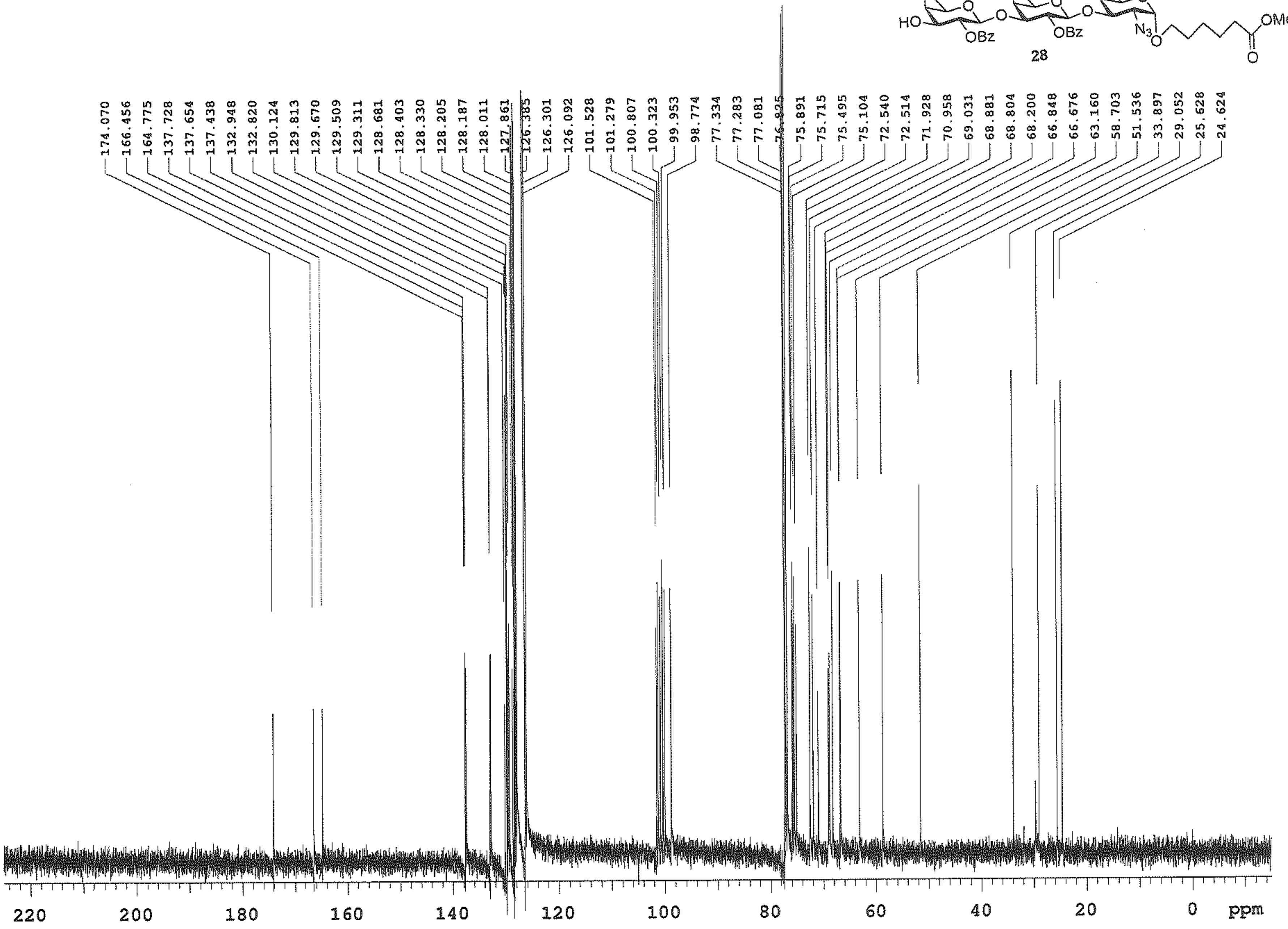
174.070
166.456
164.775
137.728
137.654
137.438
132.948
132.820
130.124
129.813
129.670
129.509
129.311
128.681
128.403
128.330
128.205
128.187
128.011
127.861
126.385
126.301
126.092
101.528
101.279
100.807
100.323
99.953
98.774
77.334
77.283
77.081
76.825
75.891
75.715
75.495
75.104
72.540
72.514
71.928
70.958
69.031
68.881
68.804
68.200
66.848
66.676
63.160
58.703
51.536
33.897
29.052
25.628
24.624
220
200
180
160
140
120
100
80
60
40
20
0
ppm

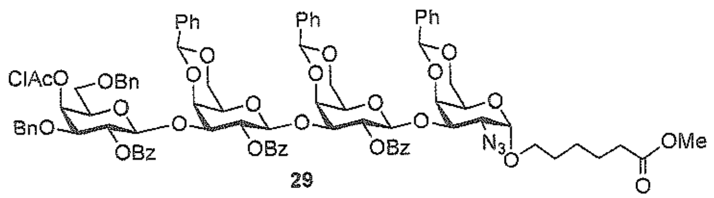

29

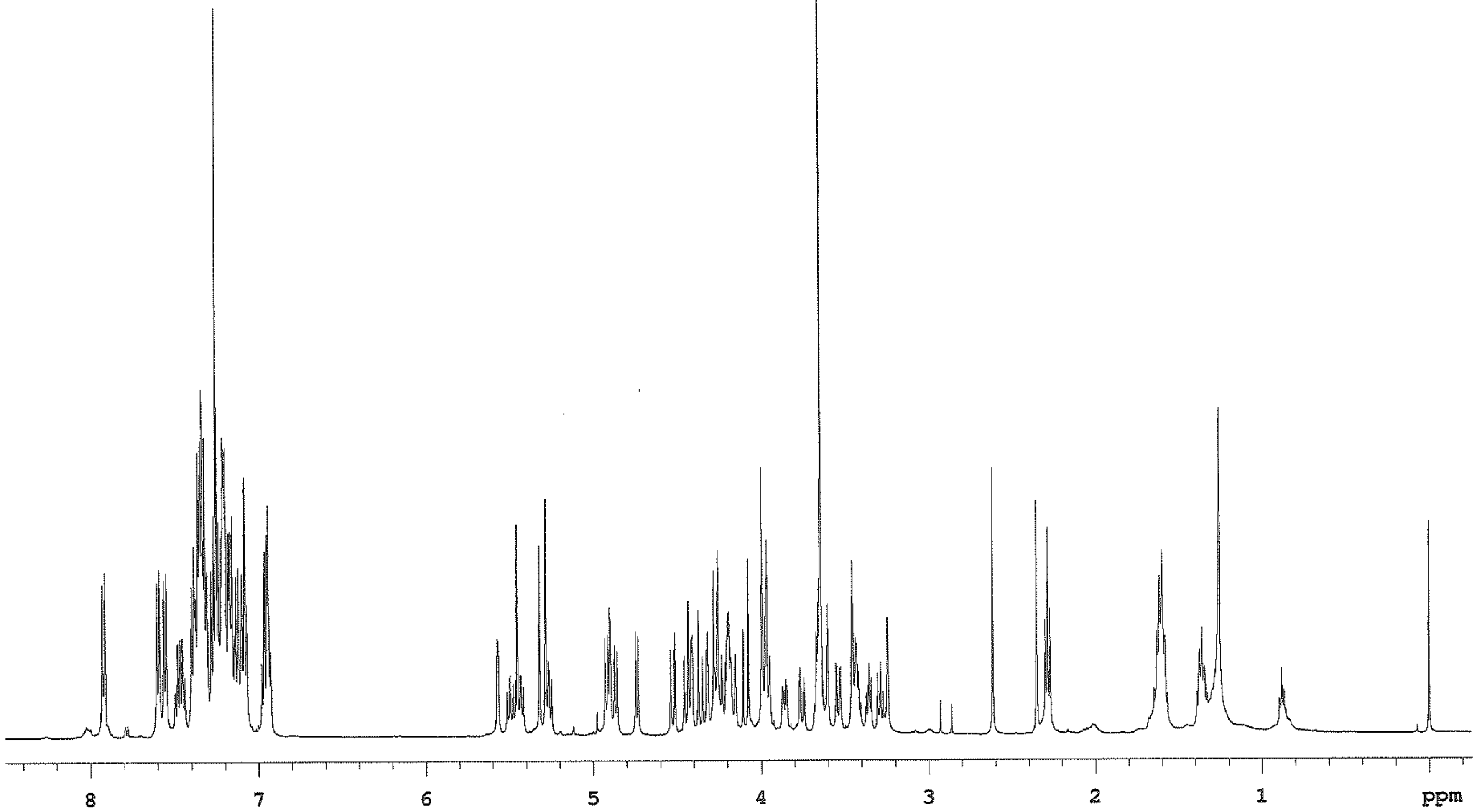

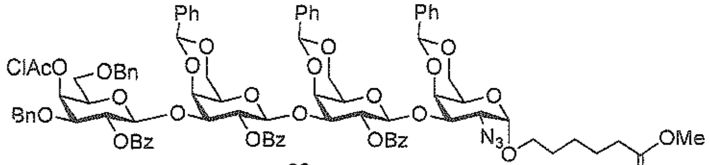
ClAcO
OBn
BnO
OBz
Ph
O
N3
OMe

29

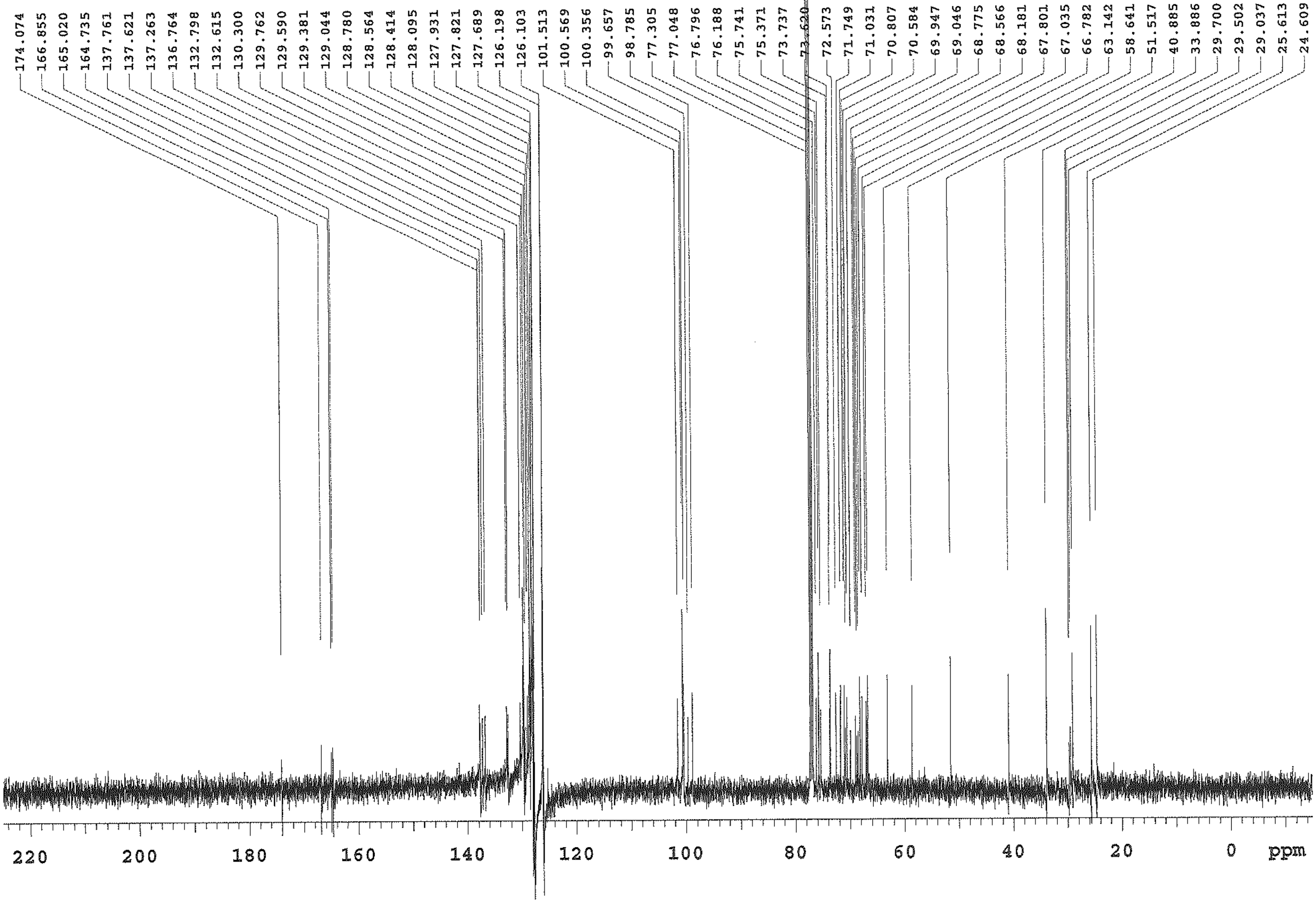
174.074
166.855
165.020
164.735
137.761
137.621
137.263
136.764
132.798
132.615
130.300
129.762
129.590
129.381
129.044
128.780
128.564
128.414
128.095
127.931
127.821
127.689
126.198
126.103
101.513
100.569
100.356
99.657
98.785
77.305
77.048
76.796
76.188
75.741
75.371
73.737
73.620
72.573
71.749
71.031
70.807
70.584
69.947
69.046
68.775
68.566
68.181
67.801
67.035
66.782
63.142
58.641
51.517
40.885
33.886
29.700
29.502
29.037
25.613
24.609
220
200
180
160
140
120
100
80
60
40
20
0
ppm

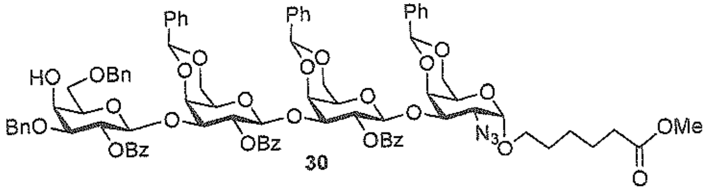

30

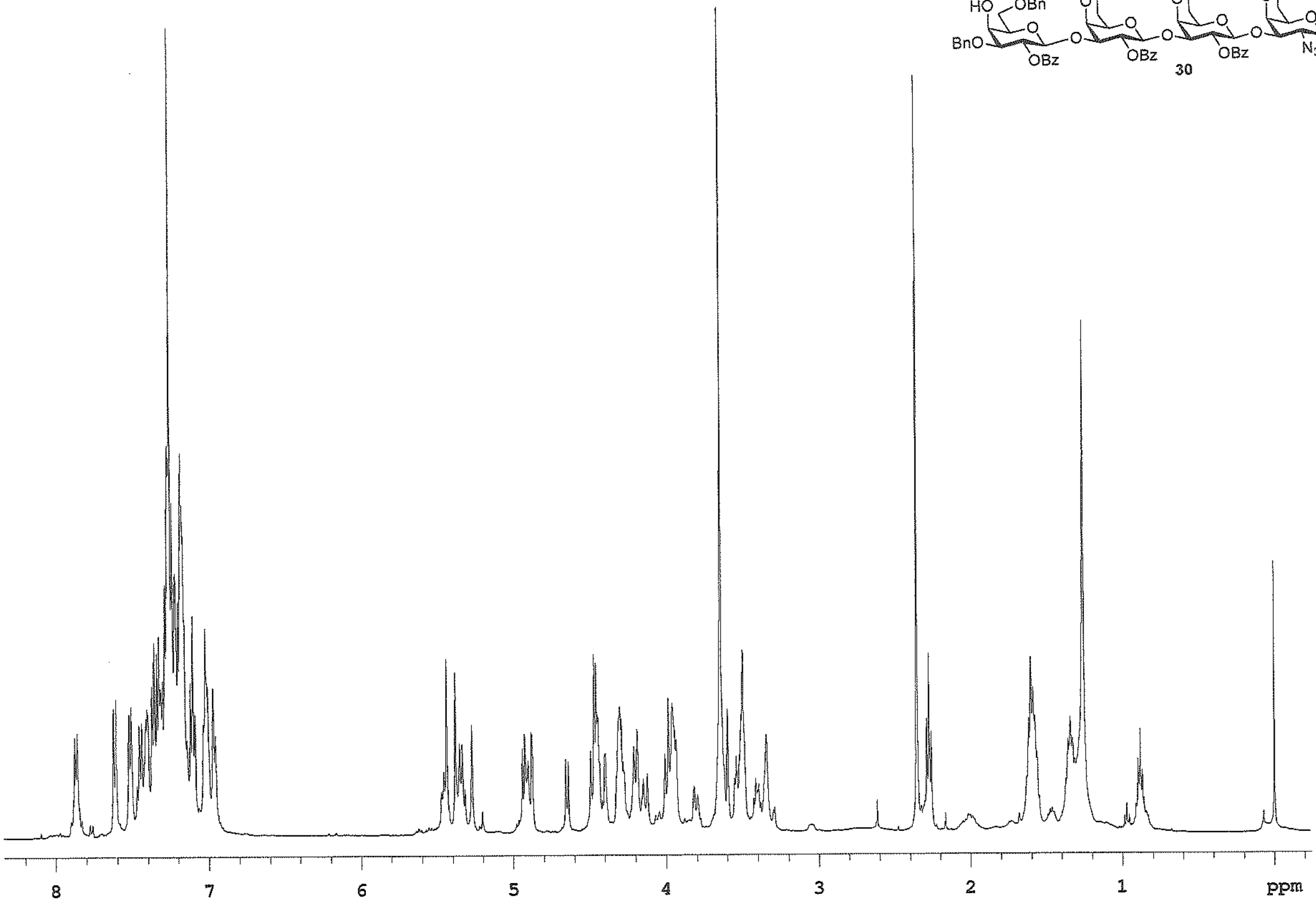

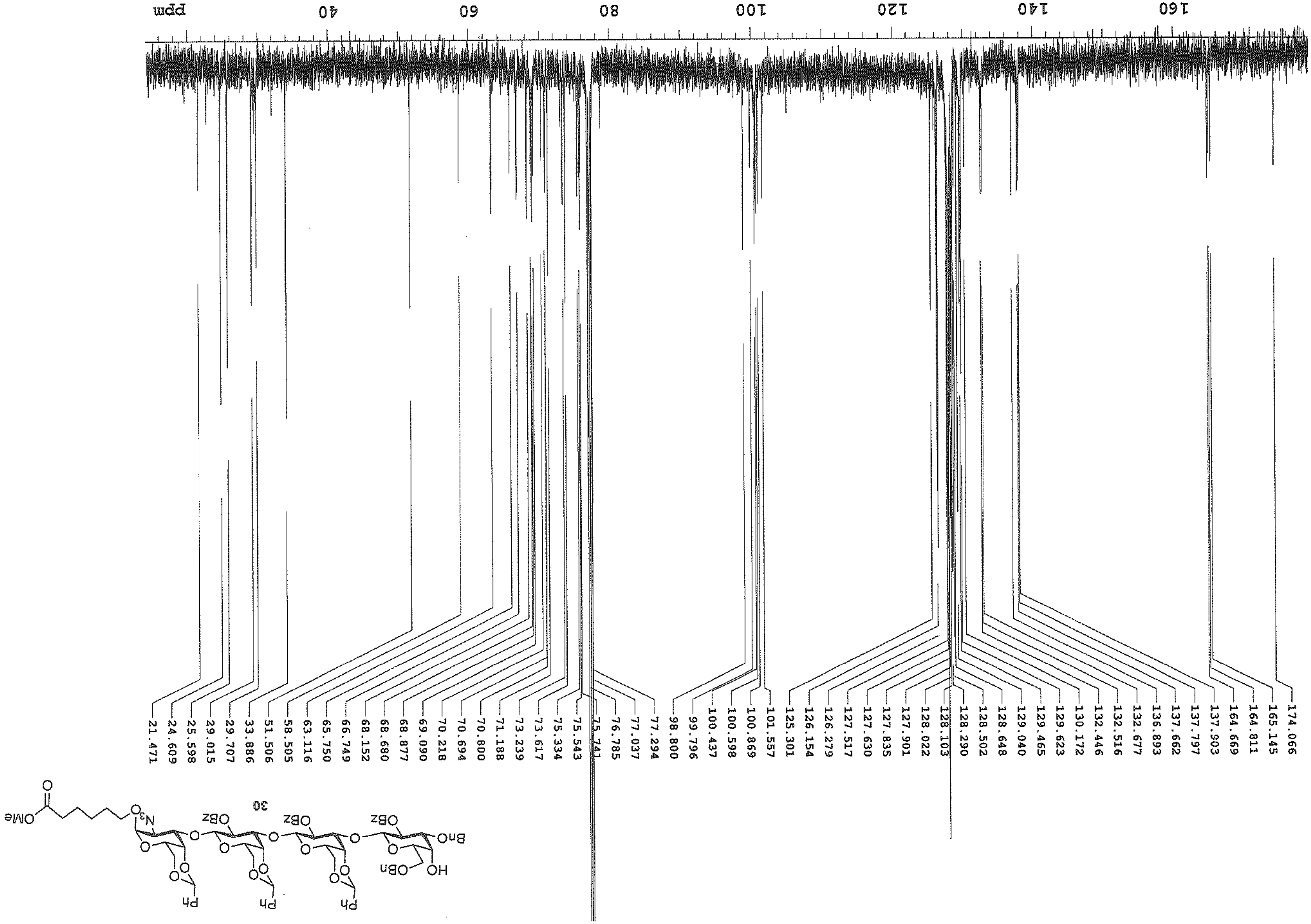

ppm
40
60
80
100
120
140
160
21.471
24.609
25.598
29.015
29.707
33.886
51.506
58.505
63.116
65.750
66.749
68.152
68.680
68.877
69.090
70.218
70.694
70.800
71.188
73.239
73.617
75.334
75.543
75.741
76.785
77.037
77.294
98.800
99.796
100.437
100.598
100.869
101.557
125.301
126.154
126.279
127.517
127.630
127.835
127.901
128.022
128.103
128.290
128.502
128.648
129.040
129.465
129.623
130.172
132.446
132.516
132.677
136.893
137.662
137.797
137.903
164.669
164.811
165.145
174.066
30
OMe
N3
OBz
BnO
HO
OBn
Ph

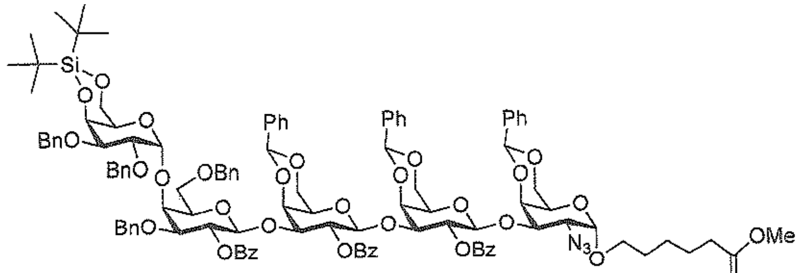

31

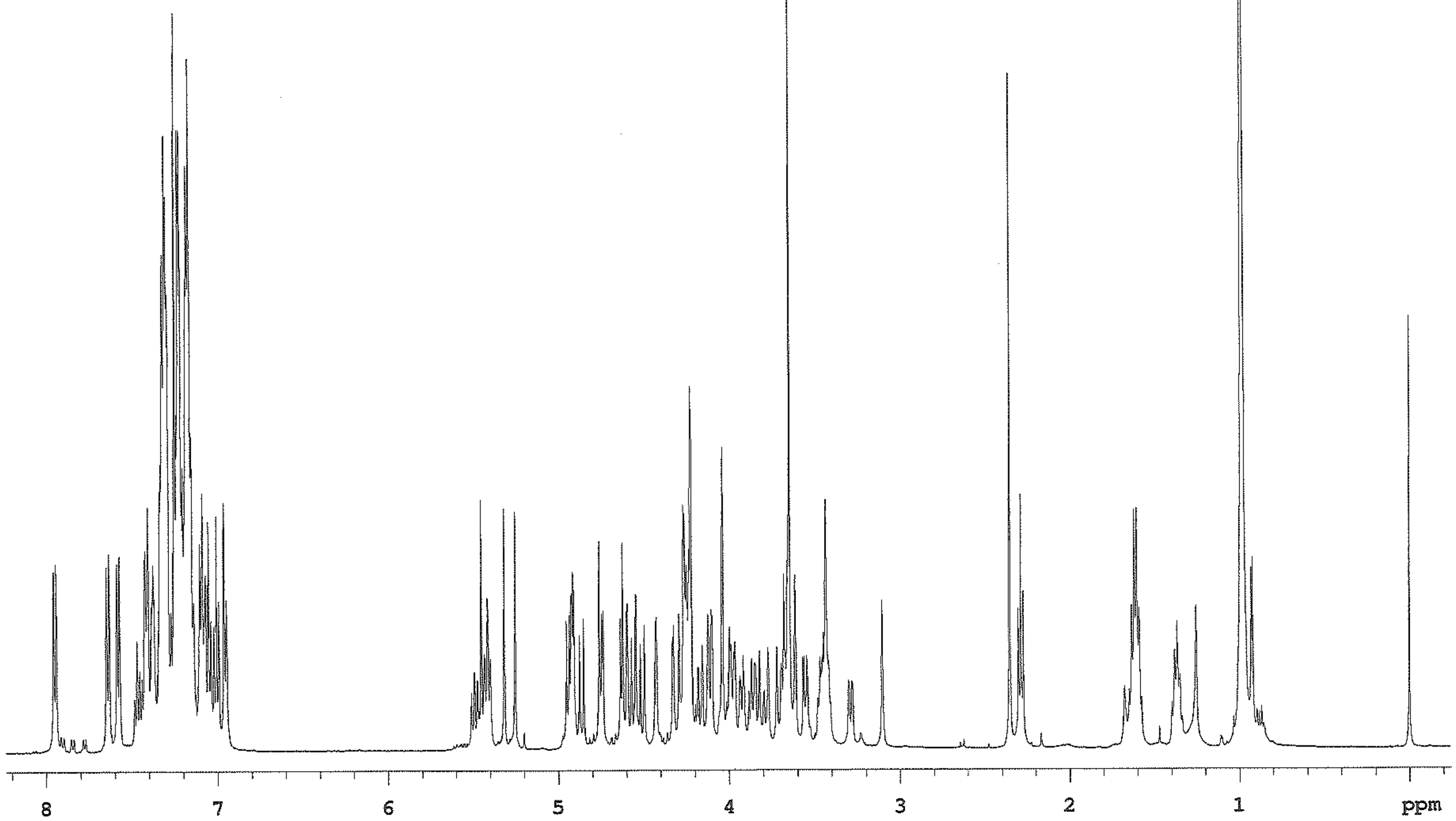

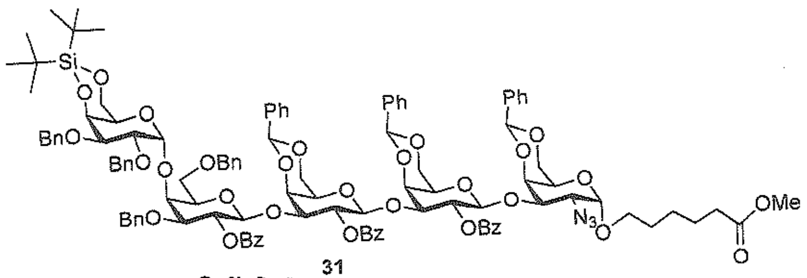
Si
BnO
BnO
OBn
BnO
OBz
Ph
OBz
Ph
OBz
Ph
N3
OMe

31

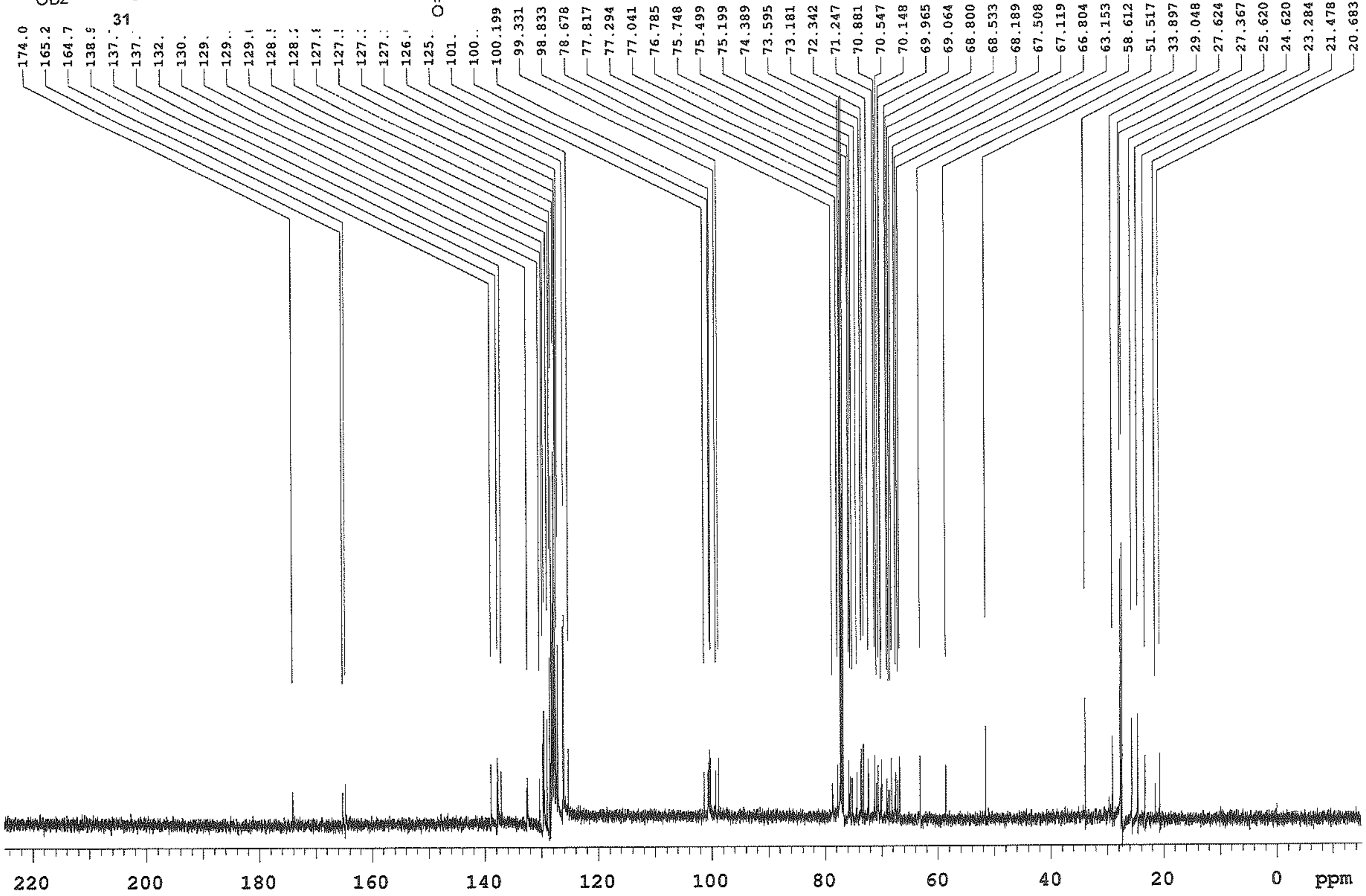
174.0
165.2
164.7
138.[illegible]
137.[illegible]
137.[illegible]
132.[illegible]
130.[illegible]
129.[illegible]
129.[illegible]
129.[illegible]
128.[illegible]
128.[illegible]
127.[illegible]
127.[illegible]
127.[illegible]
127.[illegible]
126.[illegible]
125.[illegible]
101.[illegible]
100.[illegible]
100.199
99.331
98.833
78.678
77.817
77.294
77.041
76.785
75.748
75.499
75.199
74.389
73.595
73.181
72.342
71.247
70.881
70.547
70.148
69.965
69.064
68.800
68.533
68.189
67.508
67.119
66.804
63.153
58.612
51.517
33.897
29.048
27.624
27.367
25.620
24.620
23.284
21.478
20.683
220
200
180
160
140
120
100
80
60
40
20
0
ppm

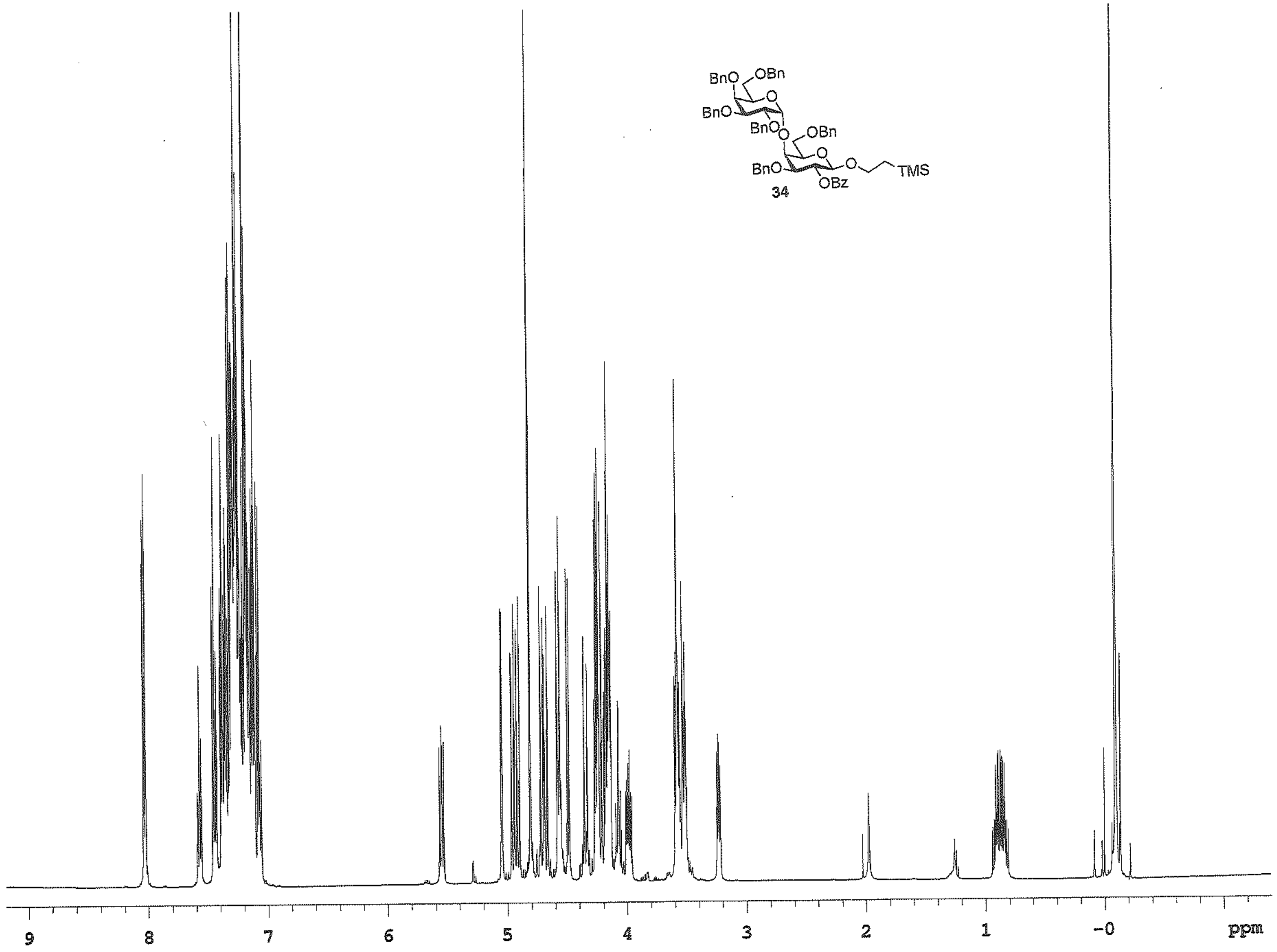
BnO
OBn
BnO
BnO
OBn
BnO
OBz
TMS
34
9
8
7
6
5
4
3
2
1
-0
ppm

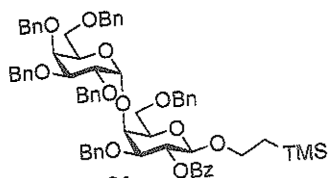

34

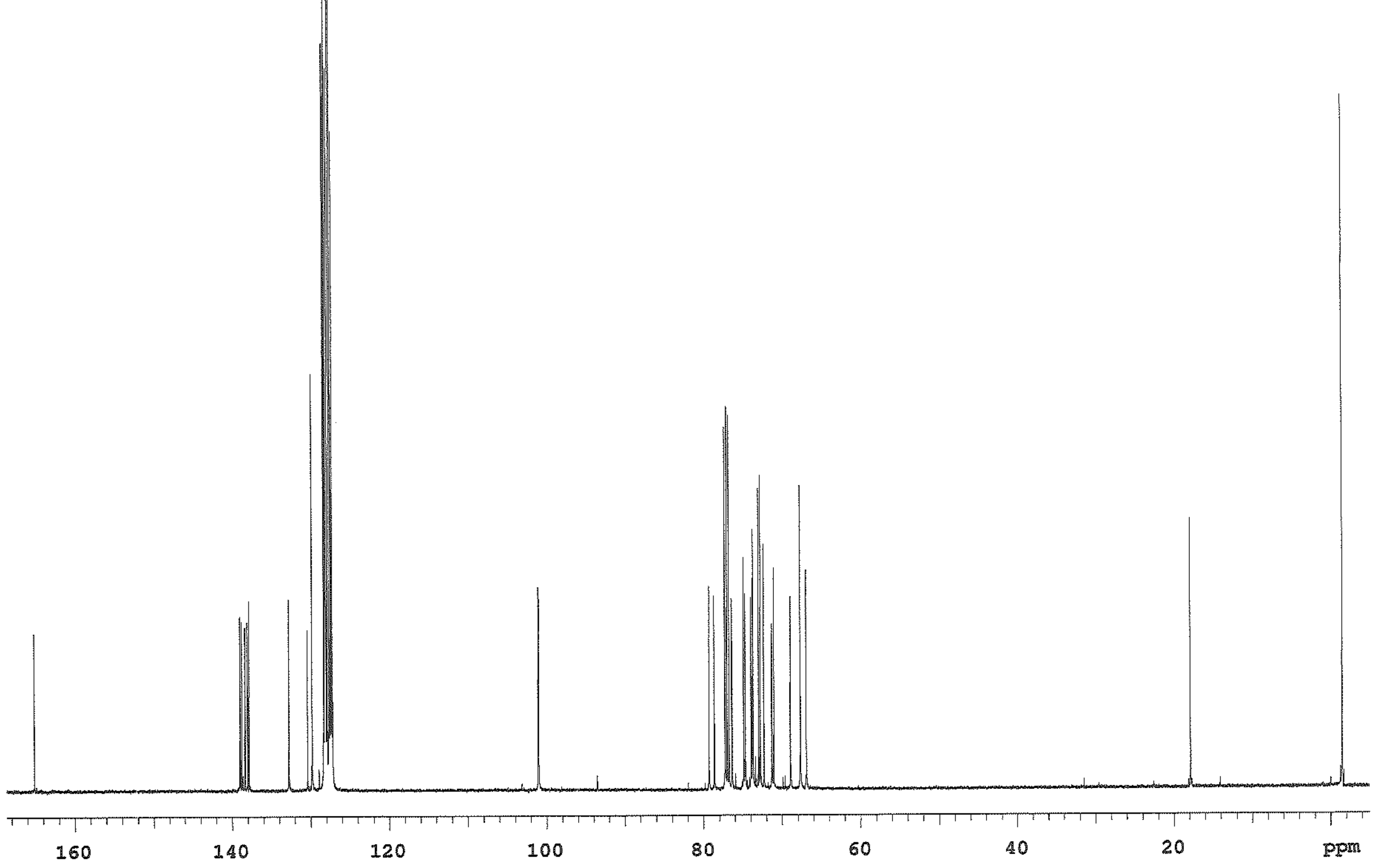

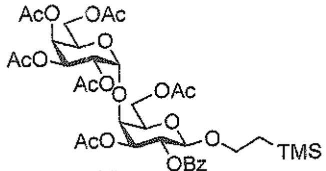

35

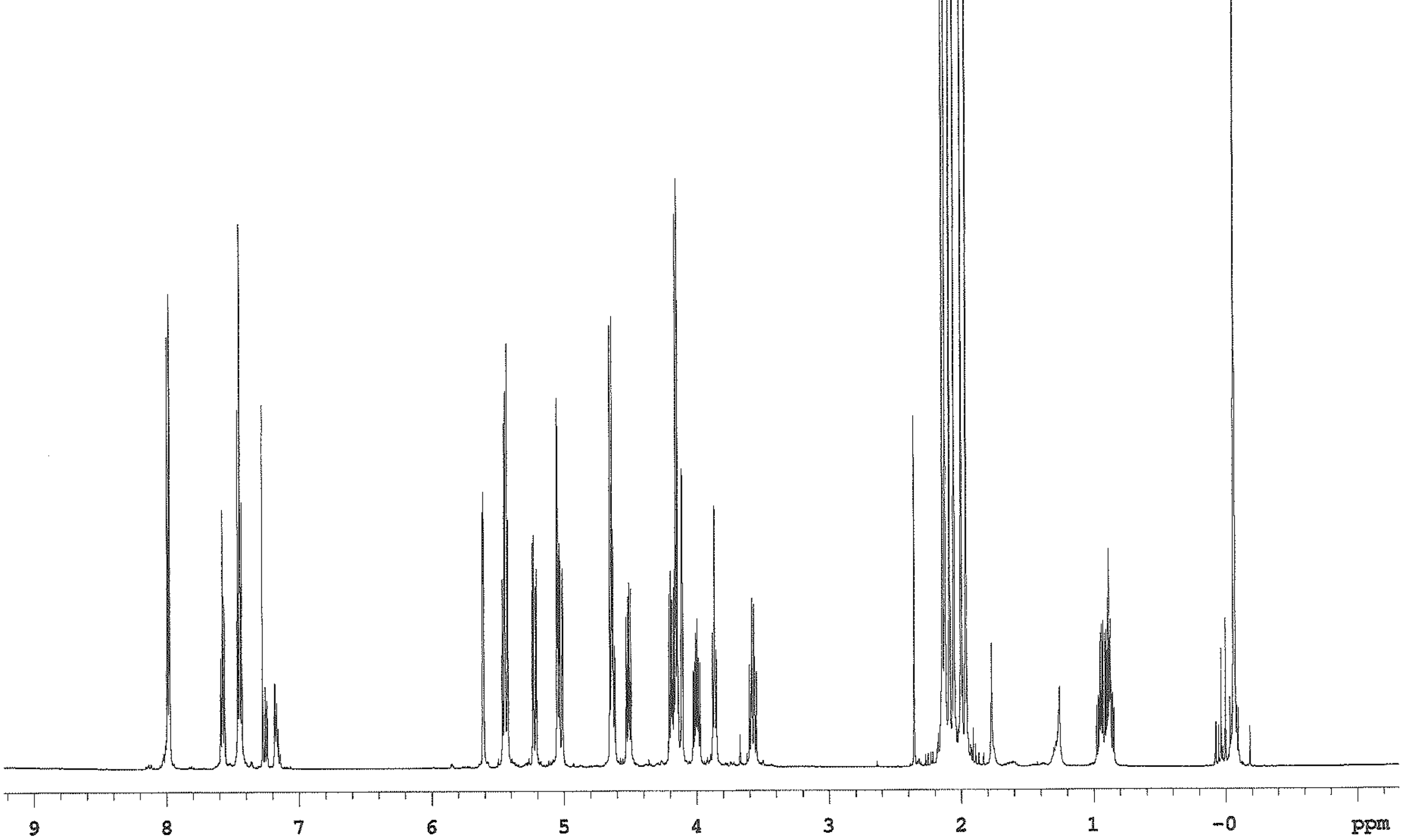

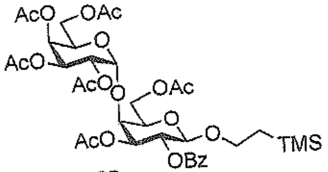

35

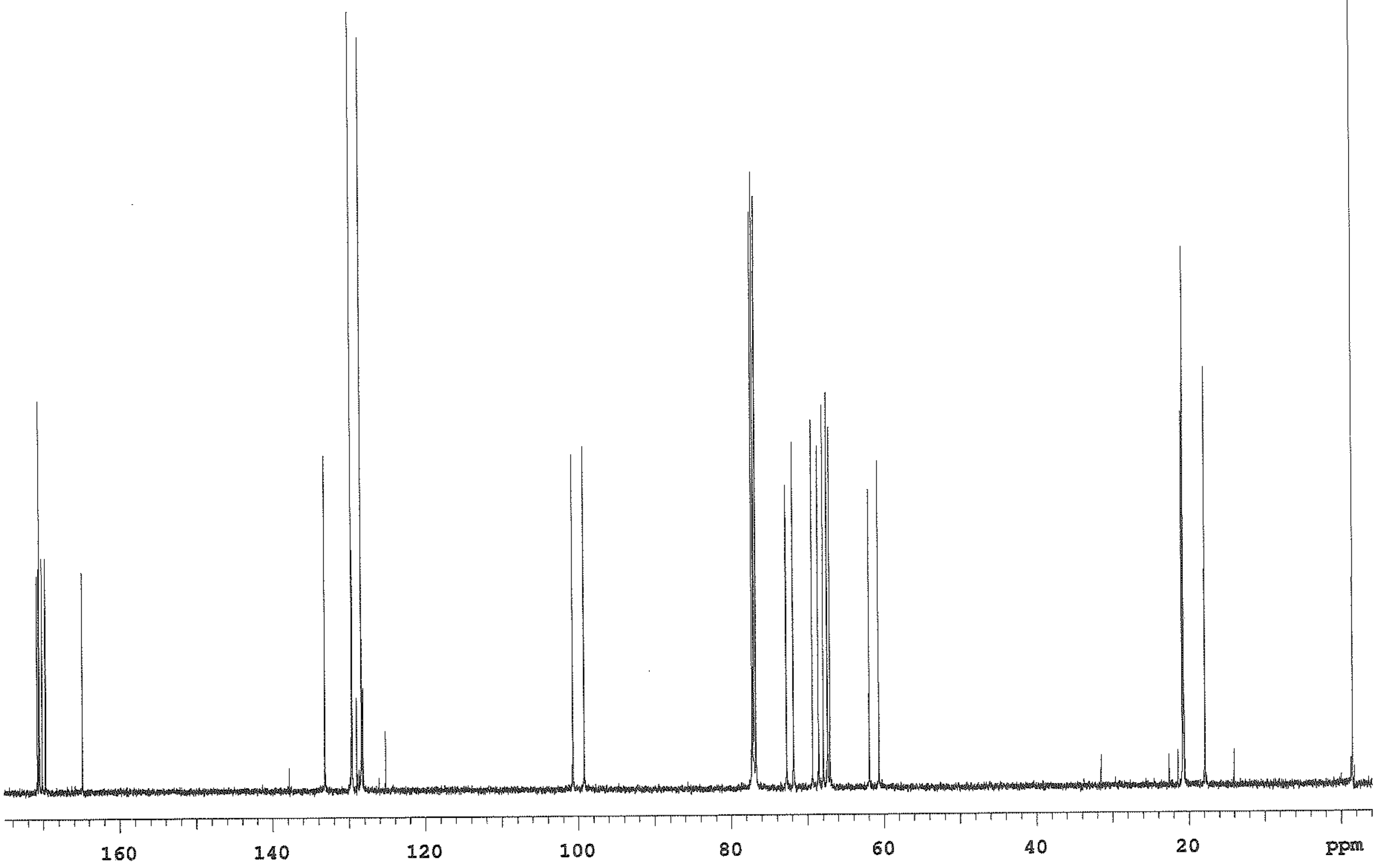

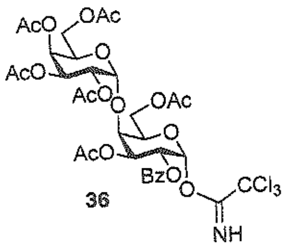

36

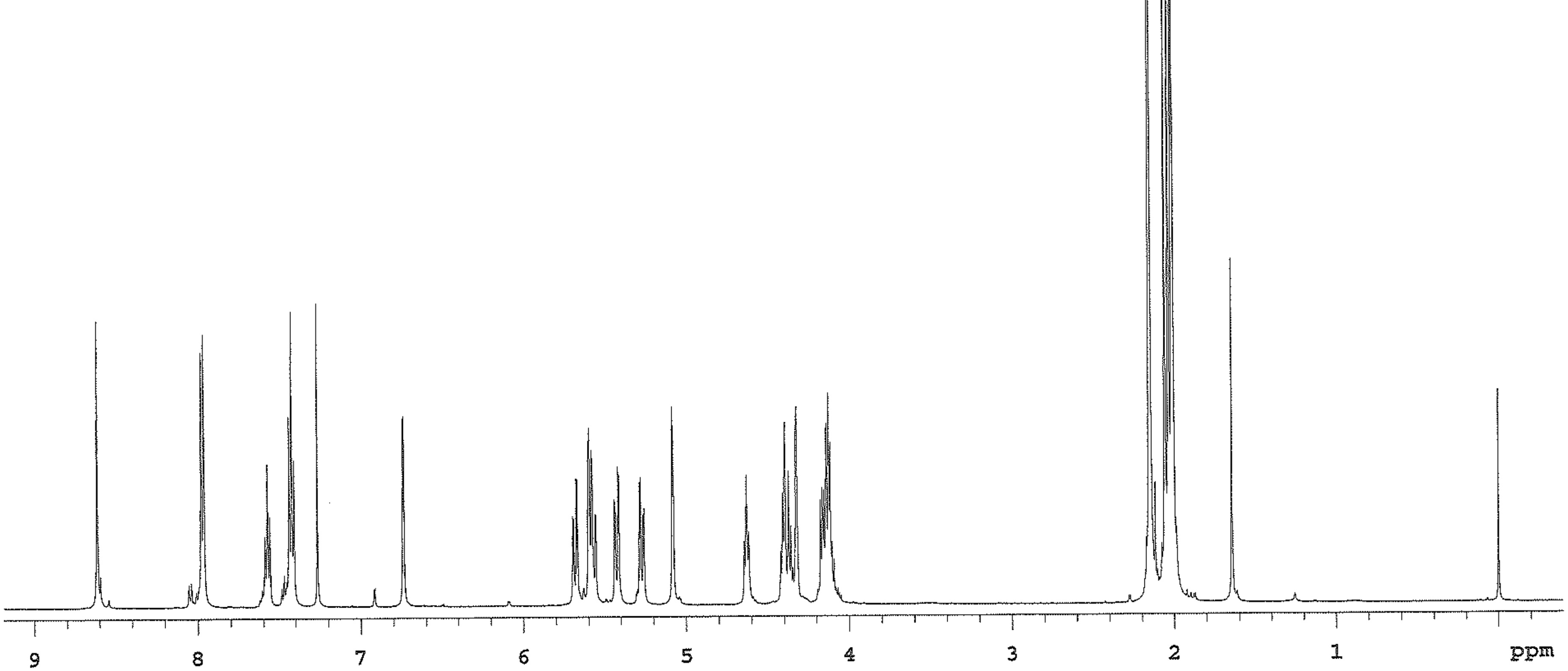

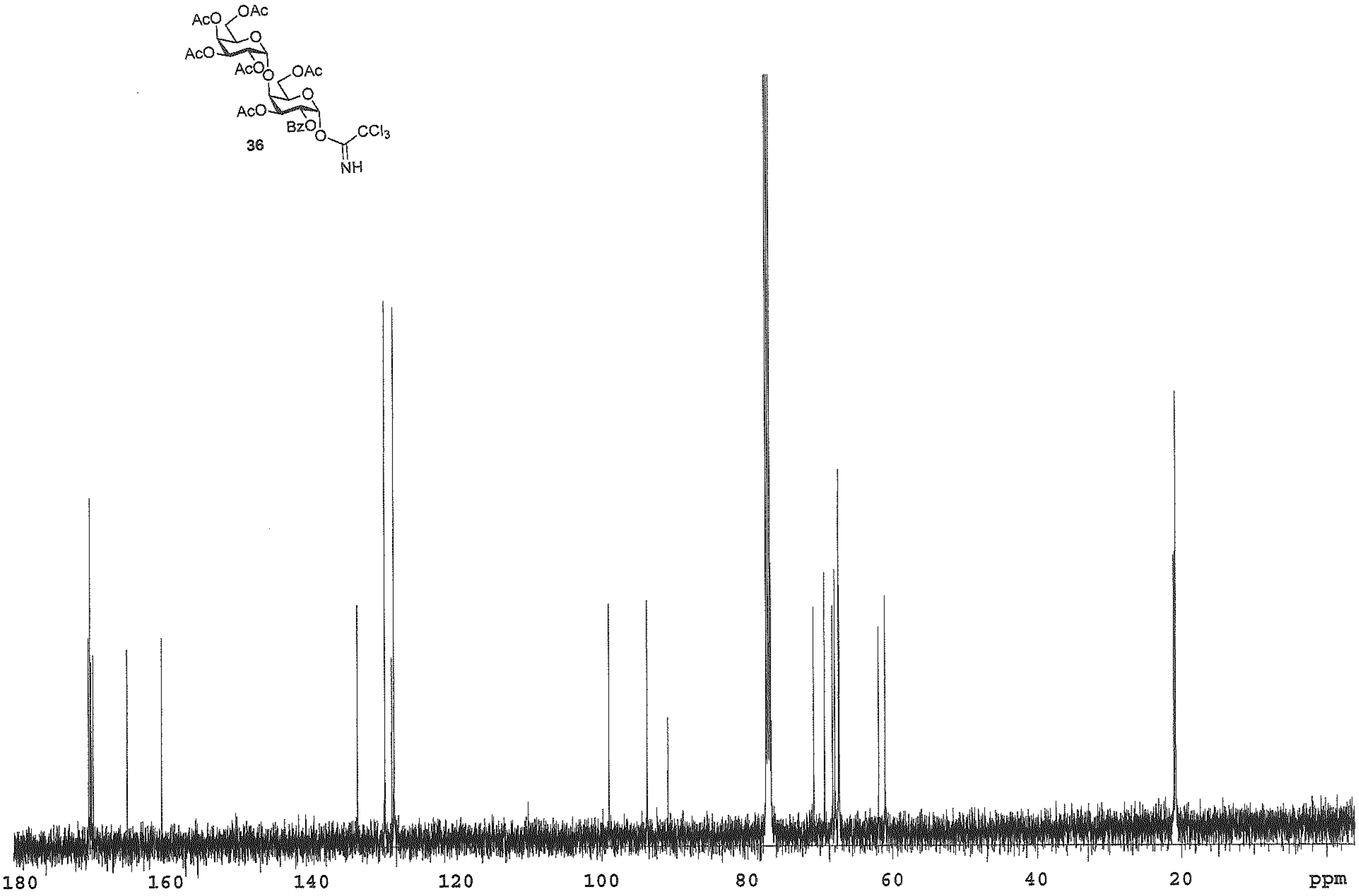
AcO
OAc
AcO
AcO
OAc
AcO
BzO
CCl3
NH
36
180
160
140
120
100
80
60
40
20
ppm

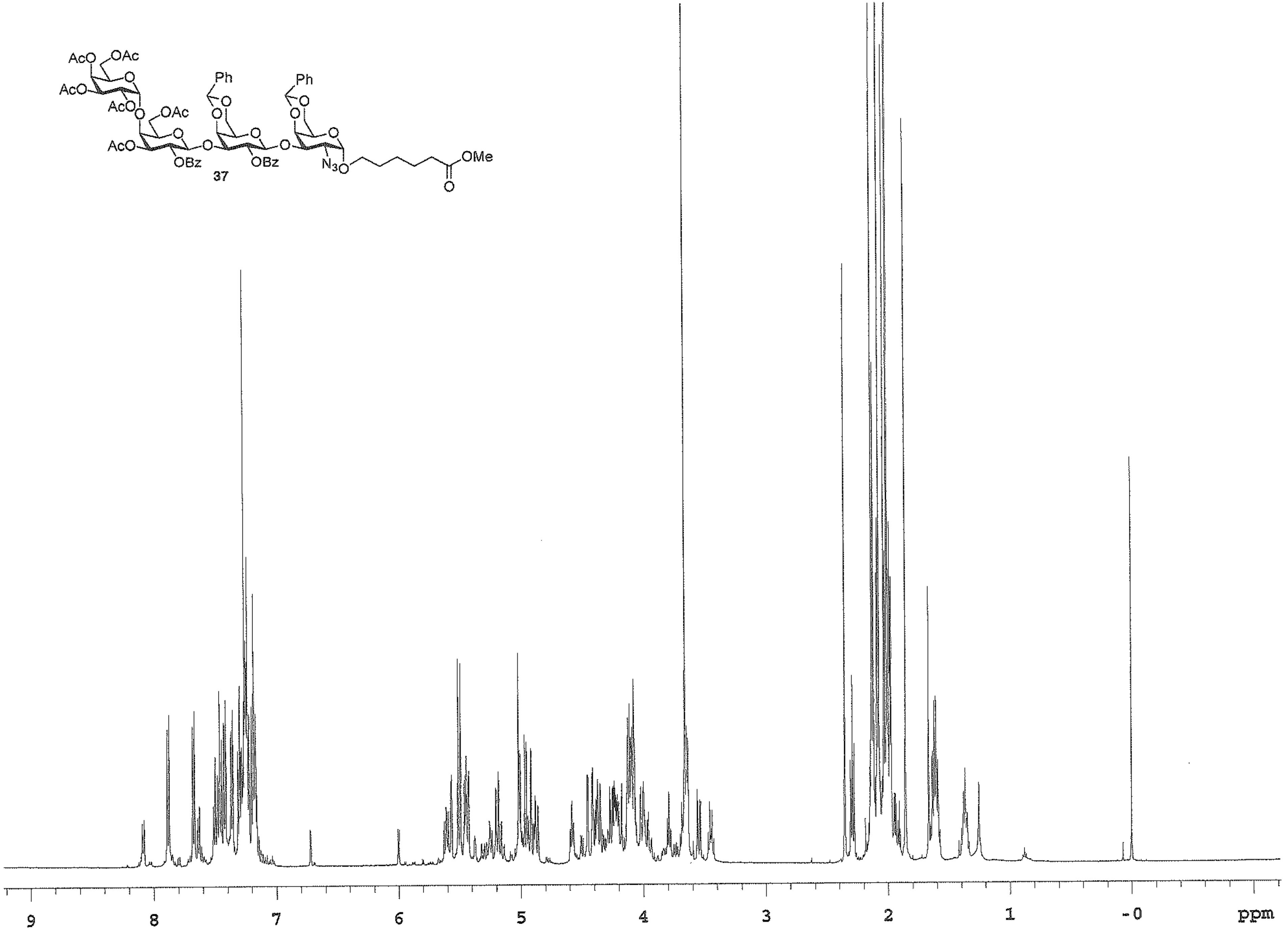

37

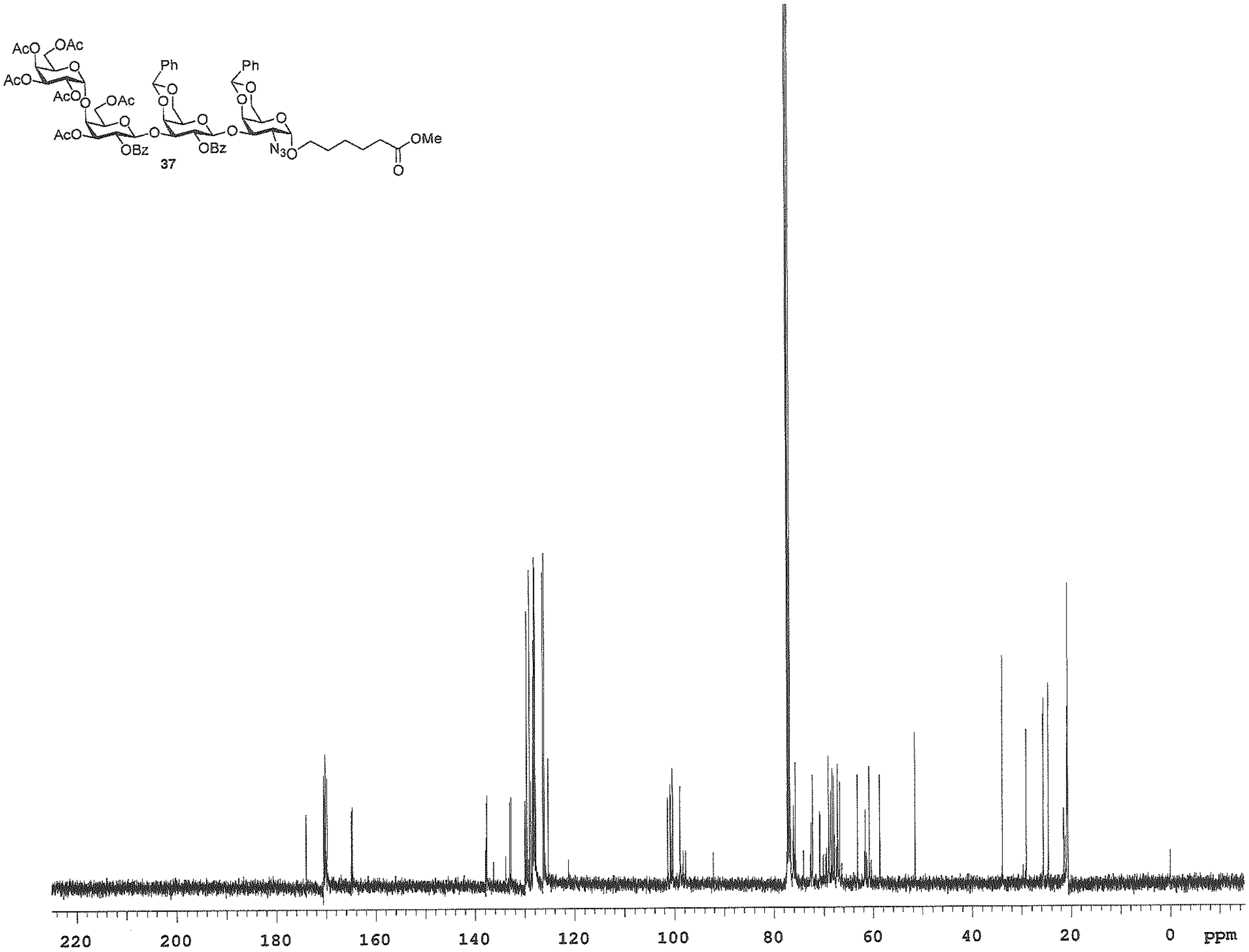

37

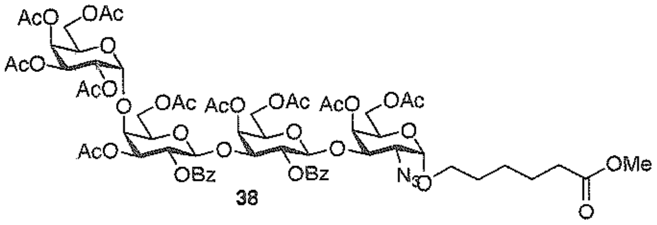
AcO
OAc
AcO
AcO
OAc
AcO
OAc
AcO
OAc
AcO
OBz
OBz
N3
O
OMe
O

38

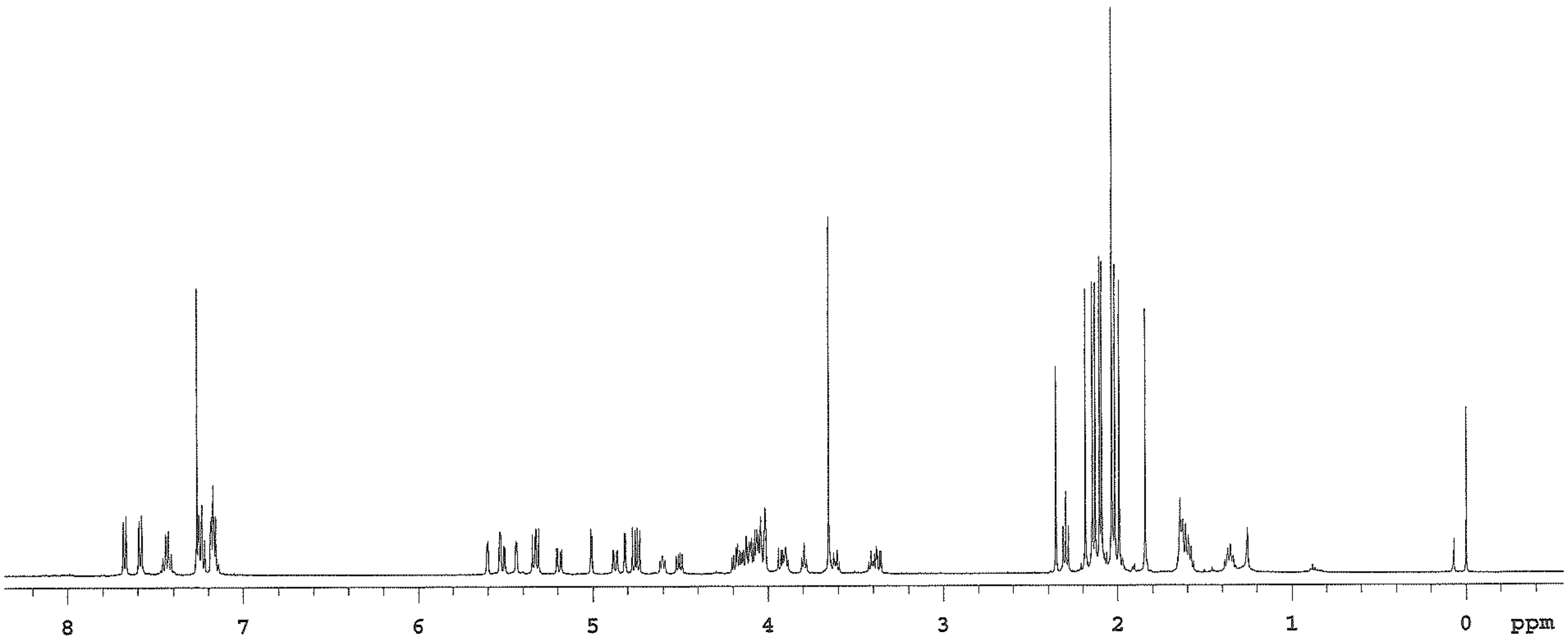
8
7
6
5
4
3
2
1
0
ppm

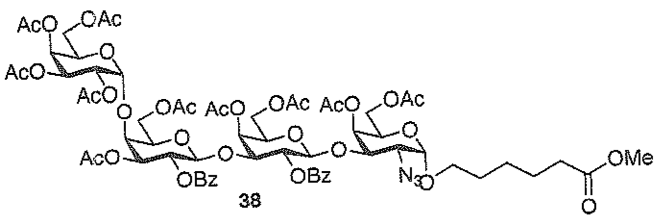

38

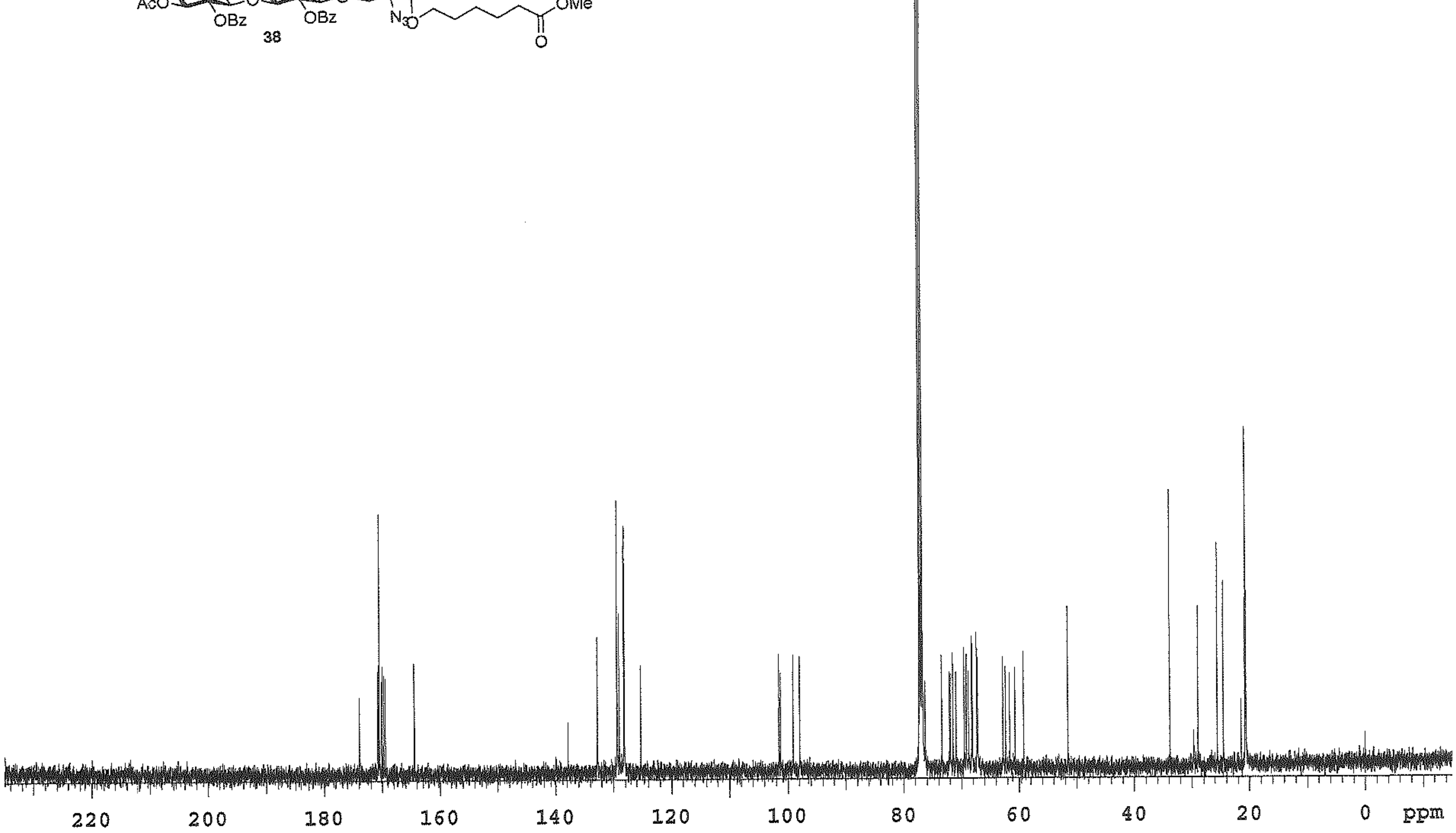

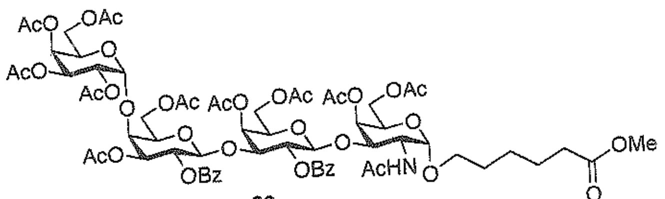

39

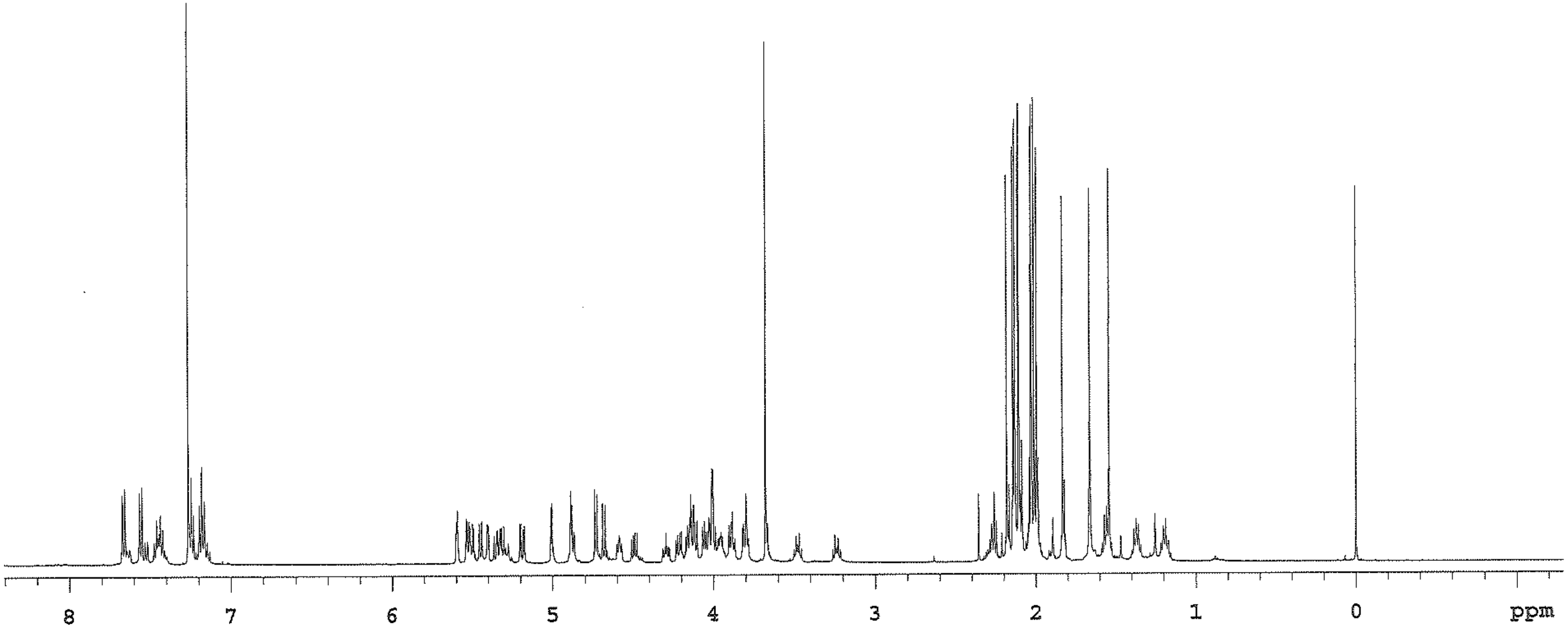

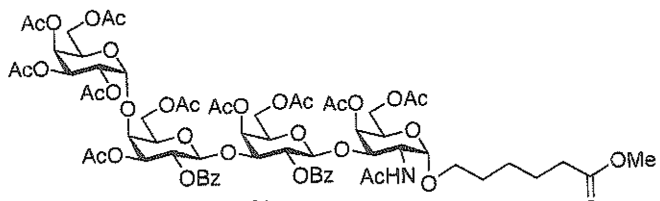
39

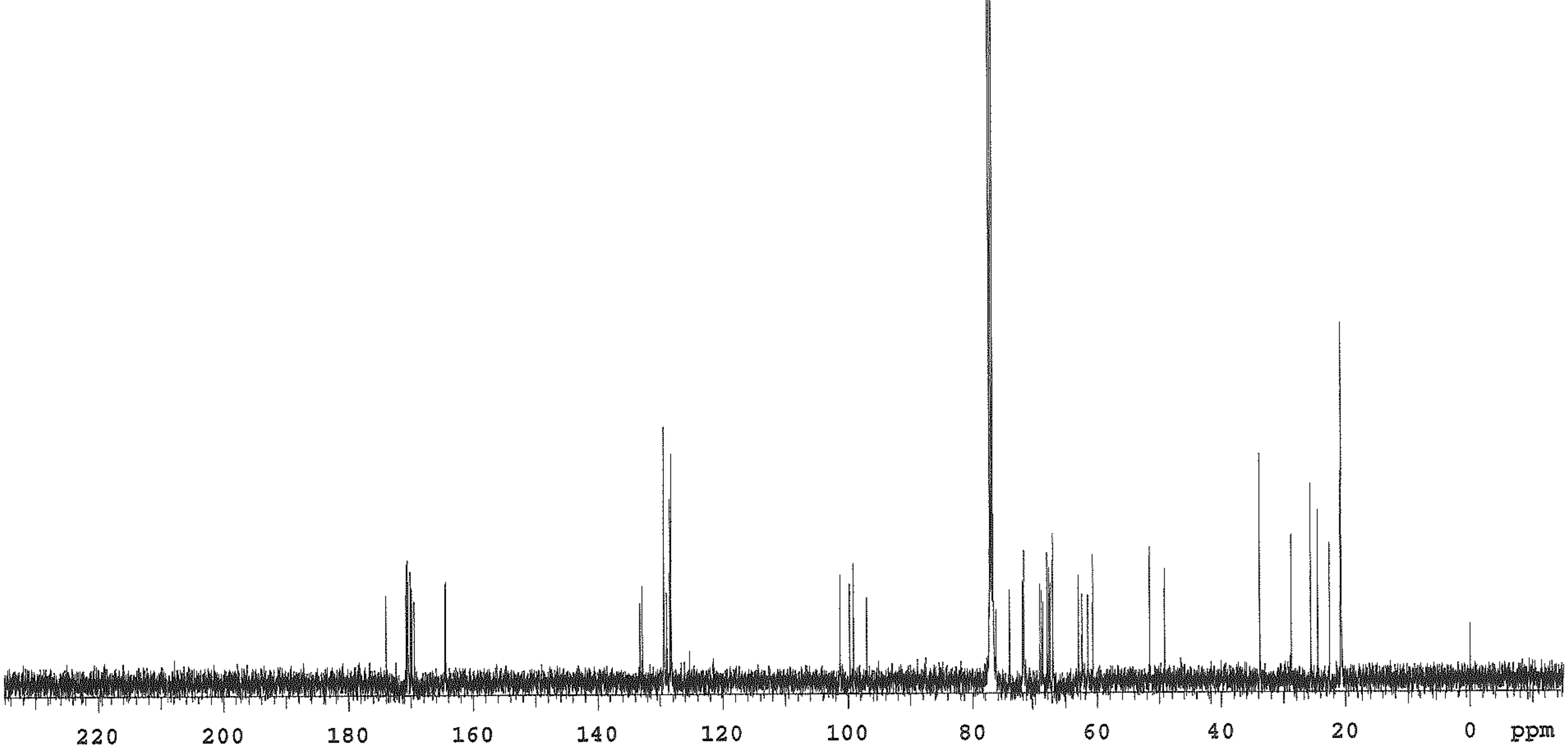

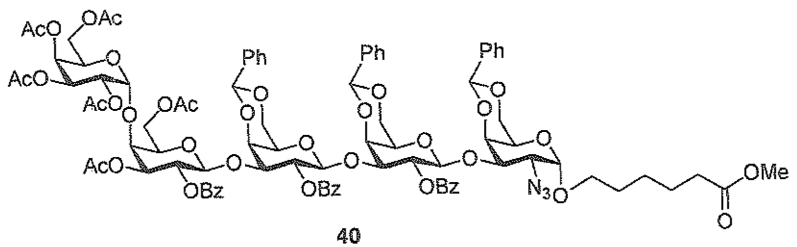
AcO
OAc
AcO
AcO
OAc
AcO
OBz
Ph
OBz
Ph
OBz
Ph
N3
OMe

40

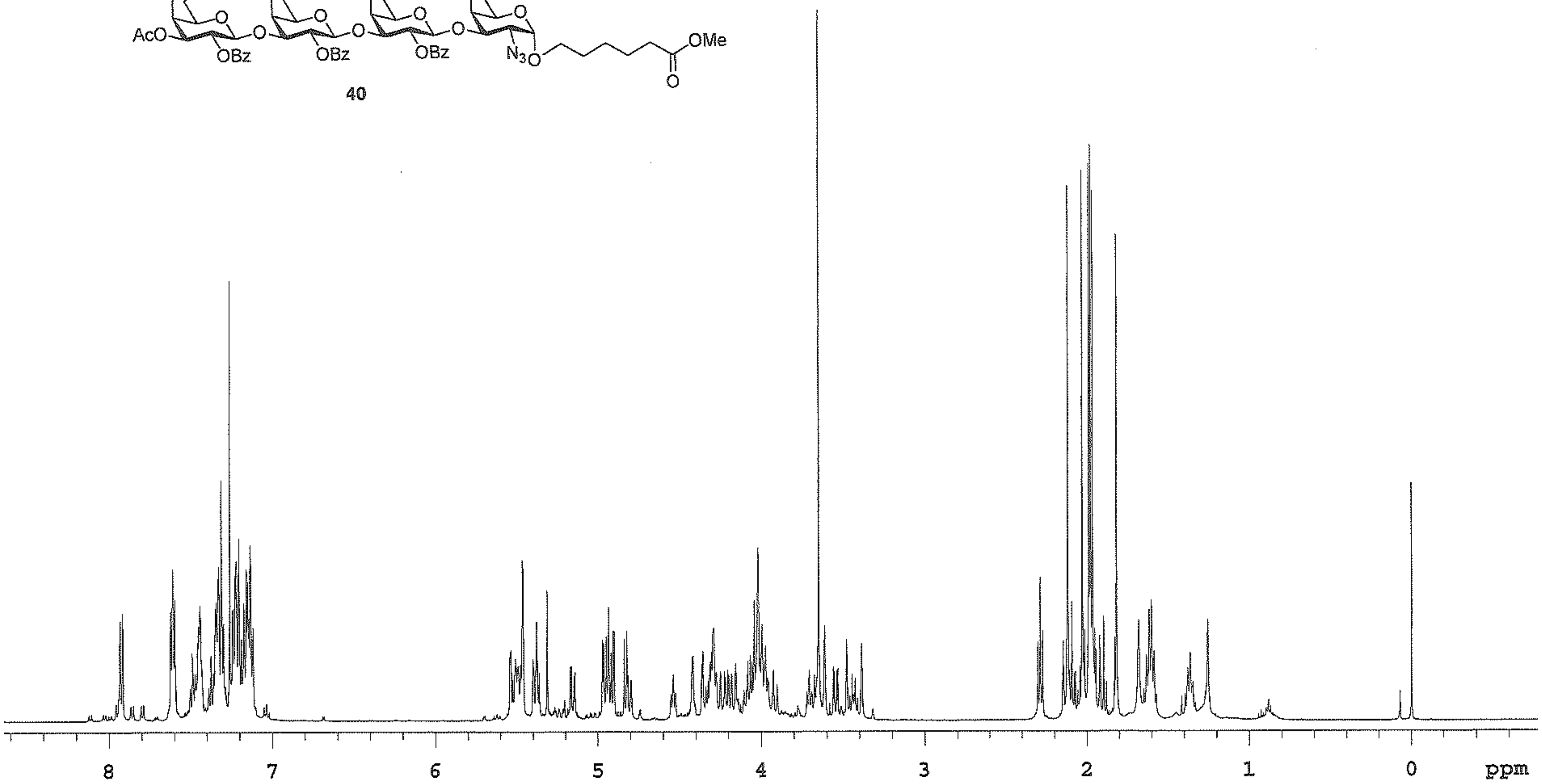
8
7
6
5
4
3
2
1
0
ppm

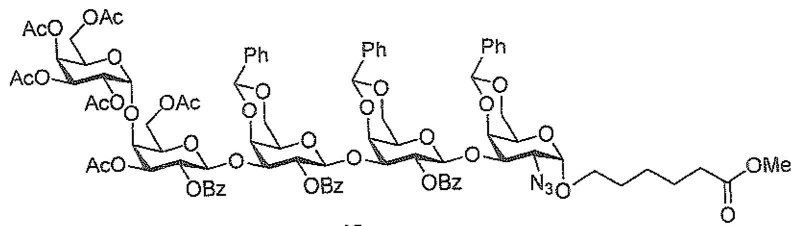
AcO
OAc
AcO
AcO
OAc
AcO
OBz
Ph
OBz
Ph
OBz
Ph
N3
OMe
O

40

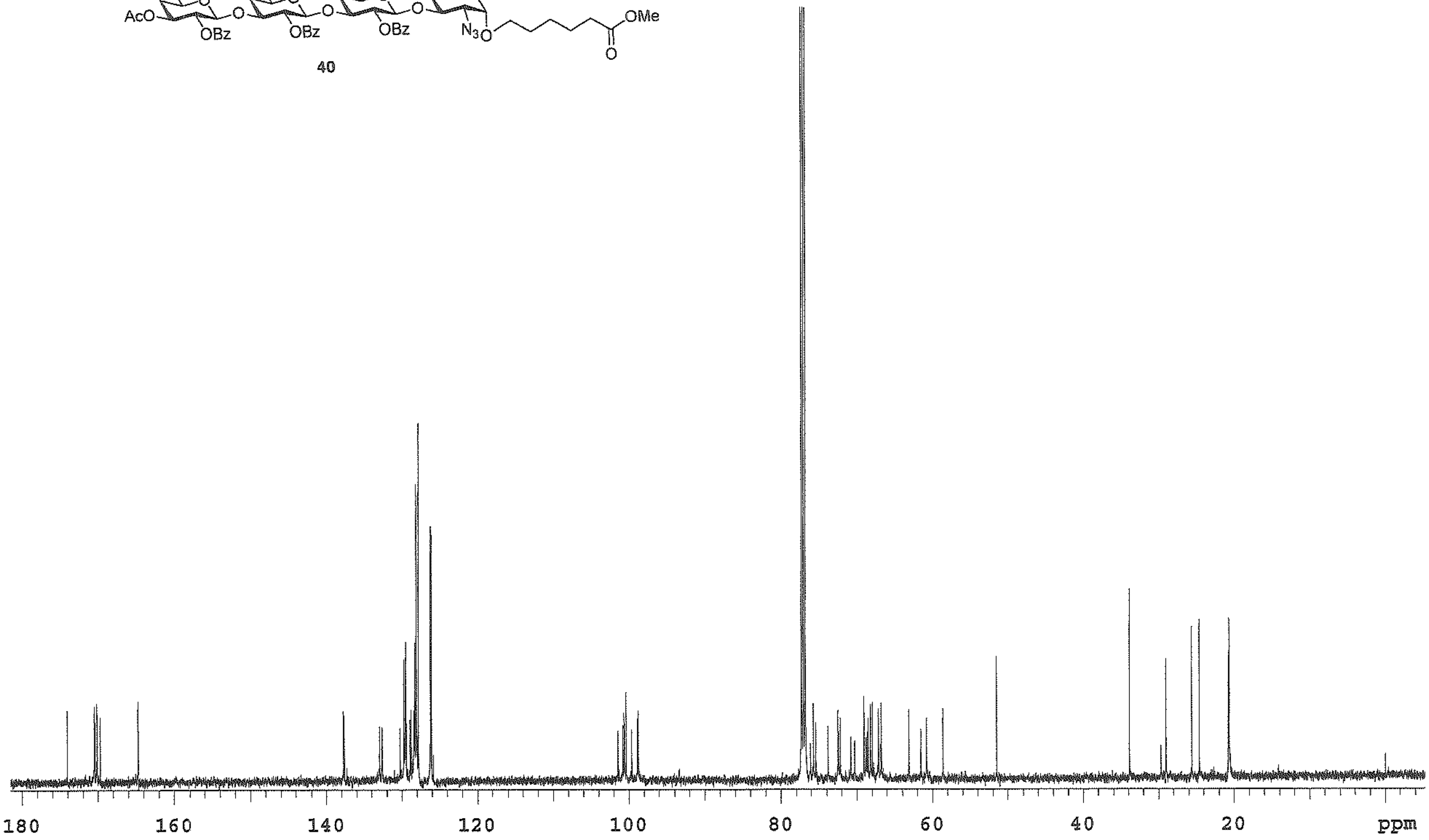
180
160
140
120
100
80
60
40
20
ppm

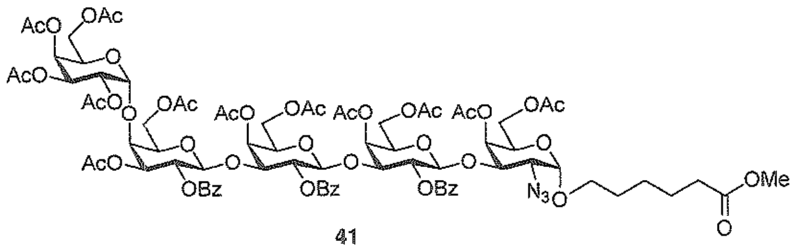
AcO
OAc
AcO
AcO
OAc
AcO
OAc
AcO
OAc
AcO
OAc
AcO
OBz
OBz
OBz
N3
OMe

41

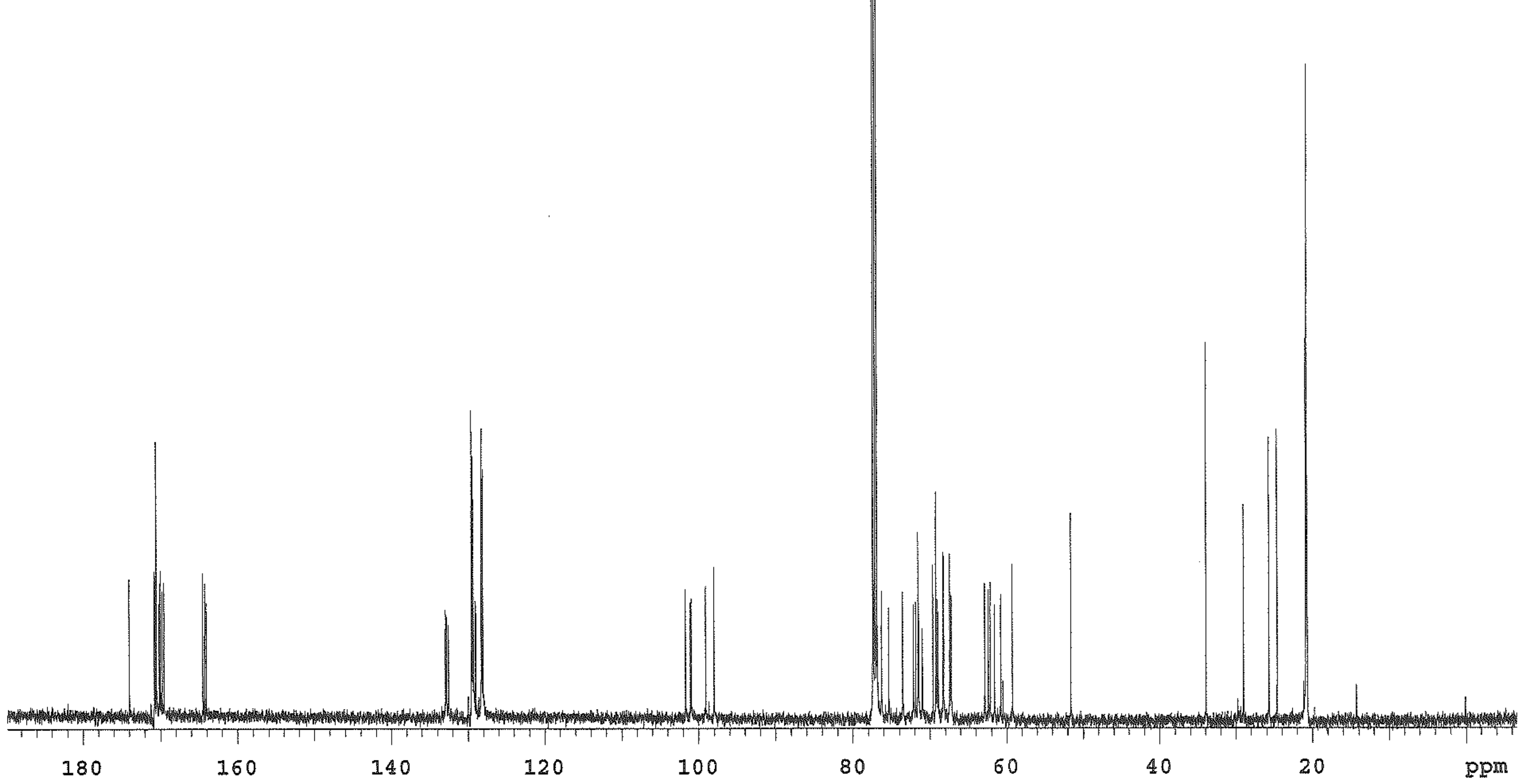
180
160
140
120
100
80
60
40
20
ppm

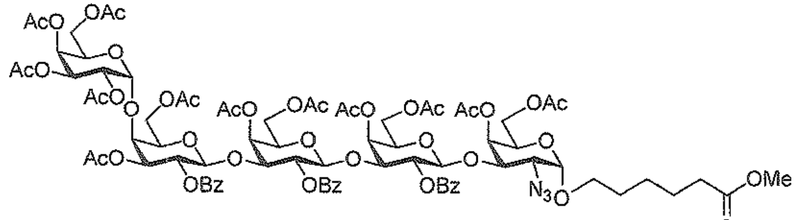
AcO
OAc
AcO
AcO
OAc
AcO
OAc
AcO
OAc
AcO
OAc
AcO
OBz
OBz
OBz
N3O
OMe
O

41

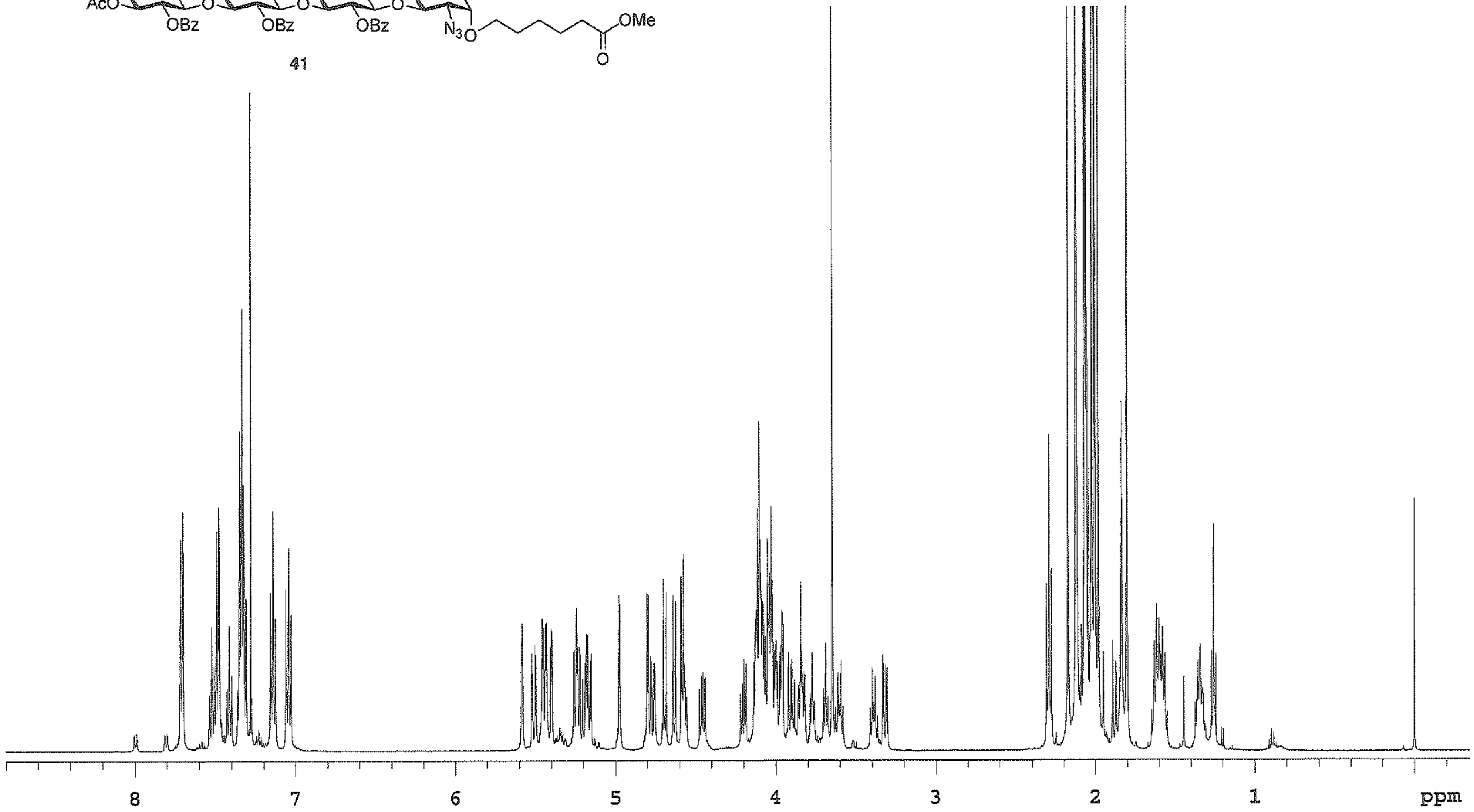
8
7
6
5
4
3
2
1
ppm

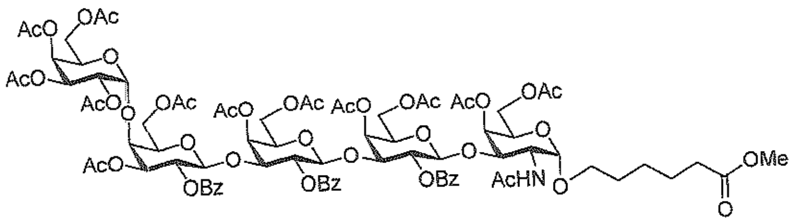
AcO
OAc
AcO
AcO
OAc
AcO
OAc
AcO
OAc
AcO
OAc
AcO
OBz
OBz
OBz
AcHN
OMe
O

42

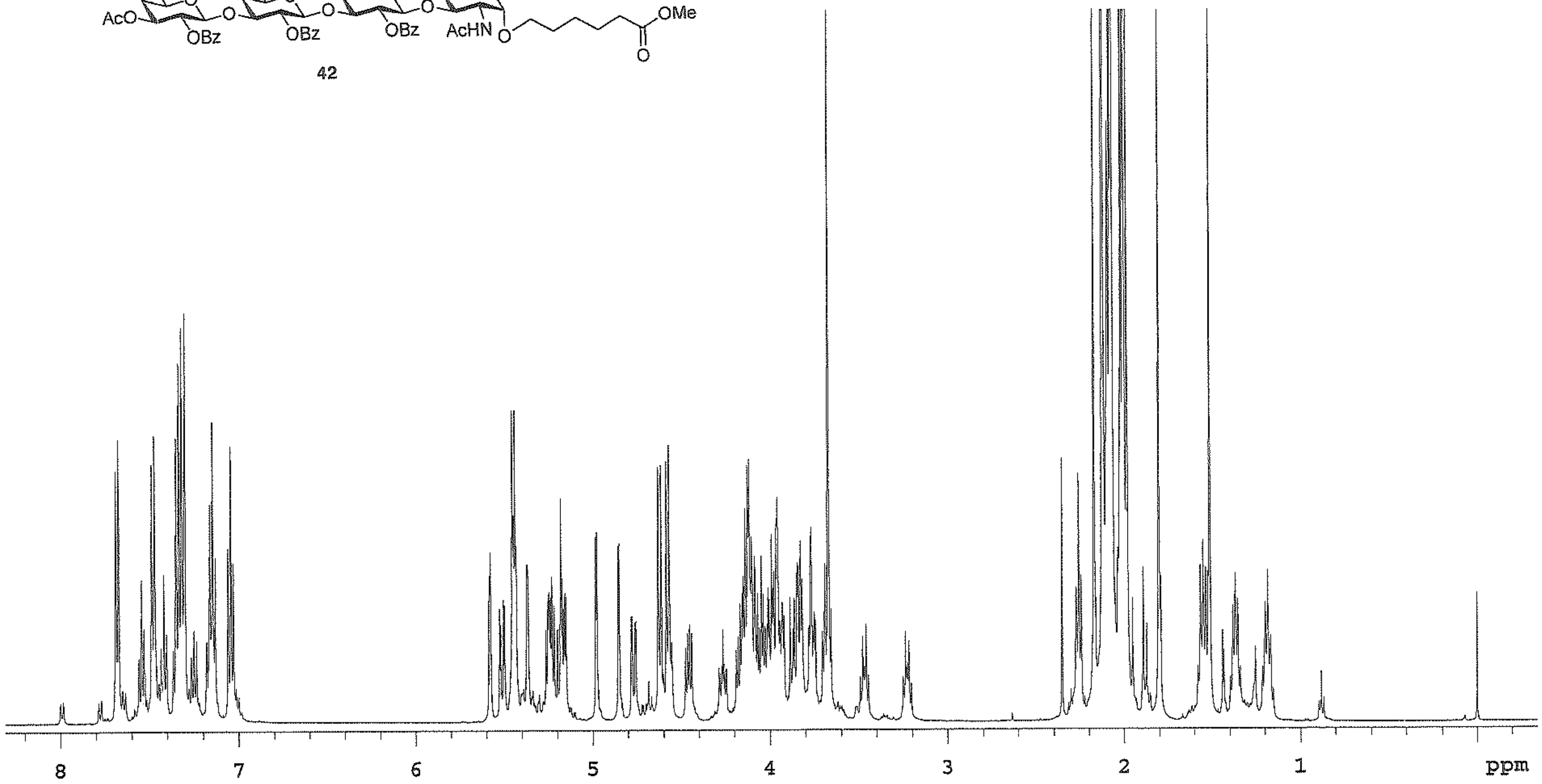
8
7
6
5
4
3
2
1
ppm

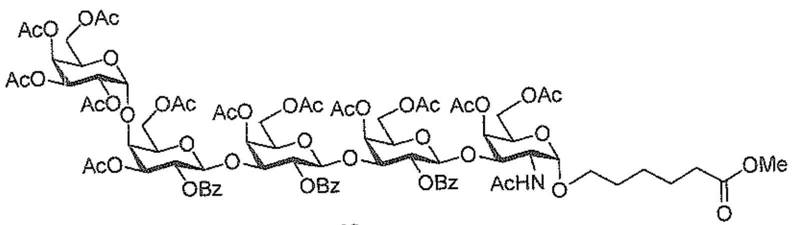
AcO
OAc
AcO
AcO
OAc
AcO
OAc
AcO
OAc
AcO
OAc
AcO
OBz
OBz
OBz
AcHN
OMe
O

42

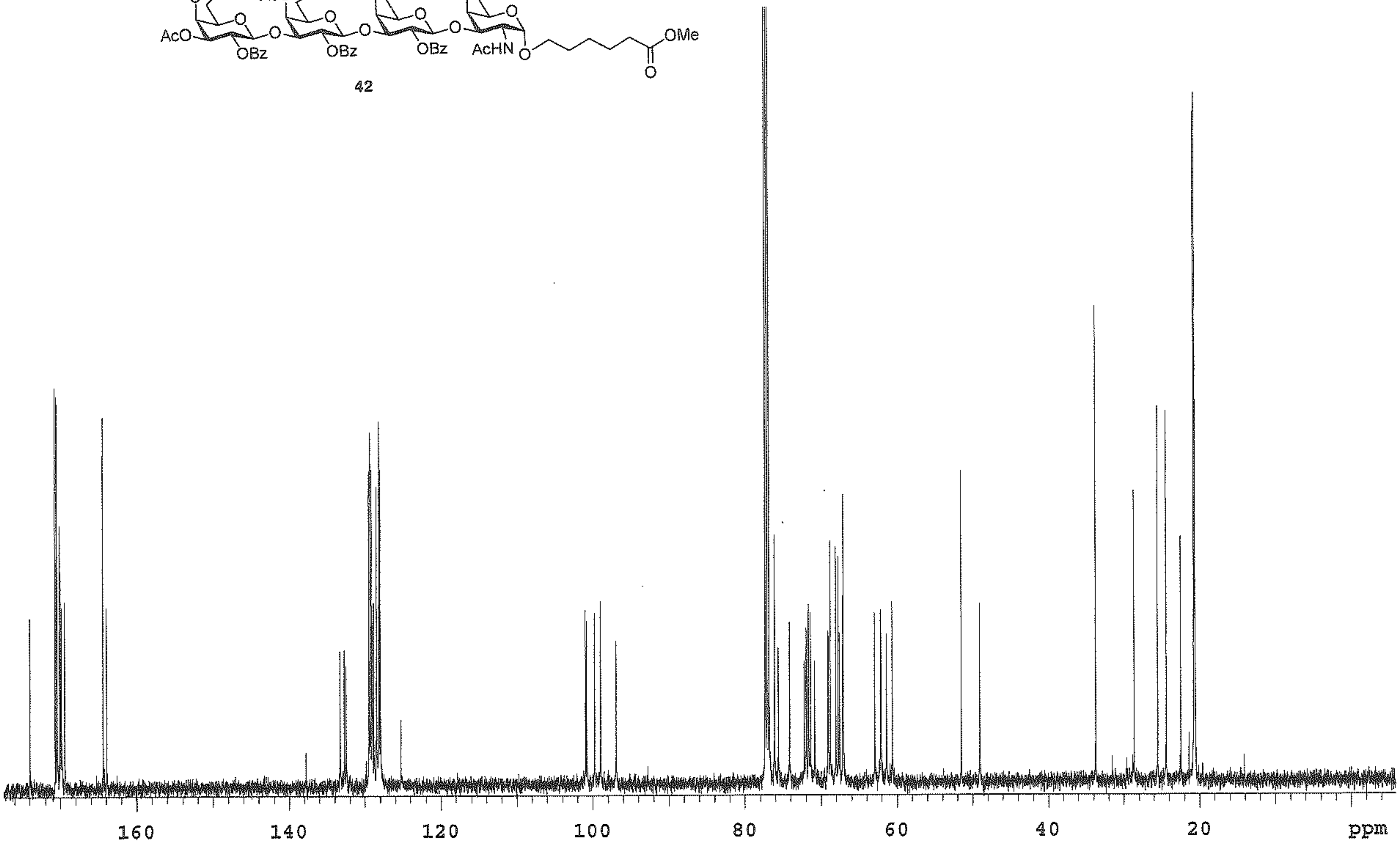
160
140
120
100
80
60
40
20
ppm

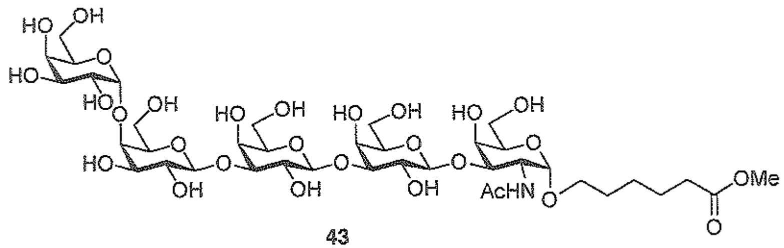

43

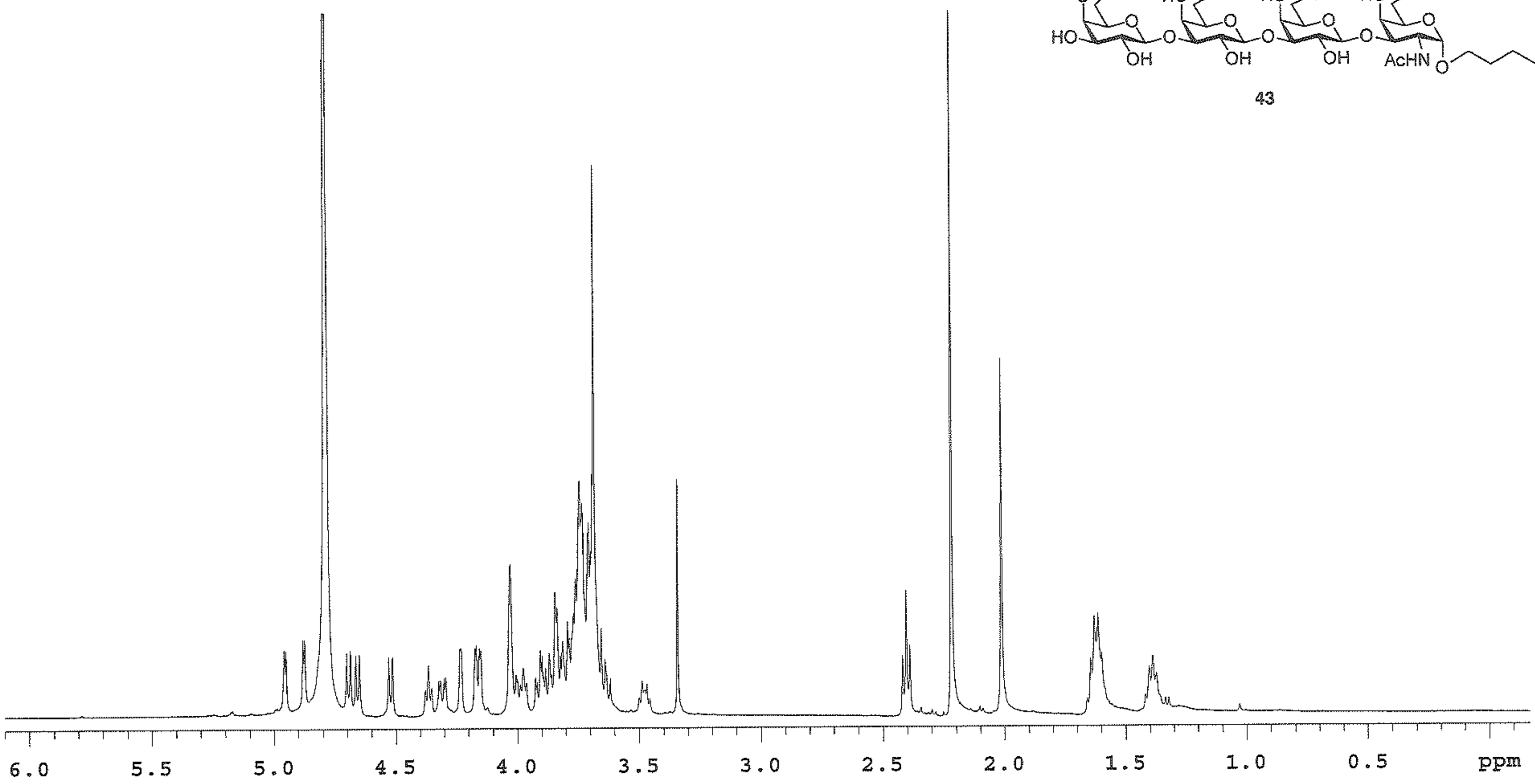

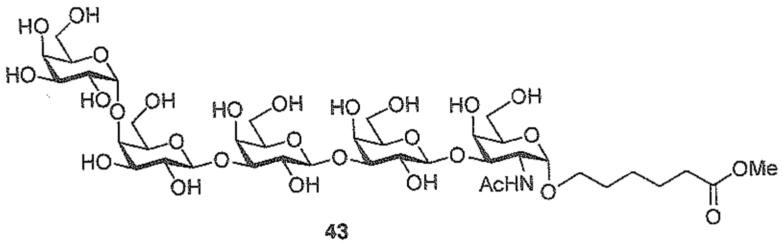

43

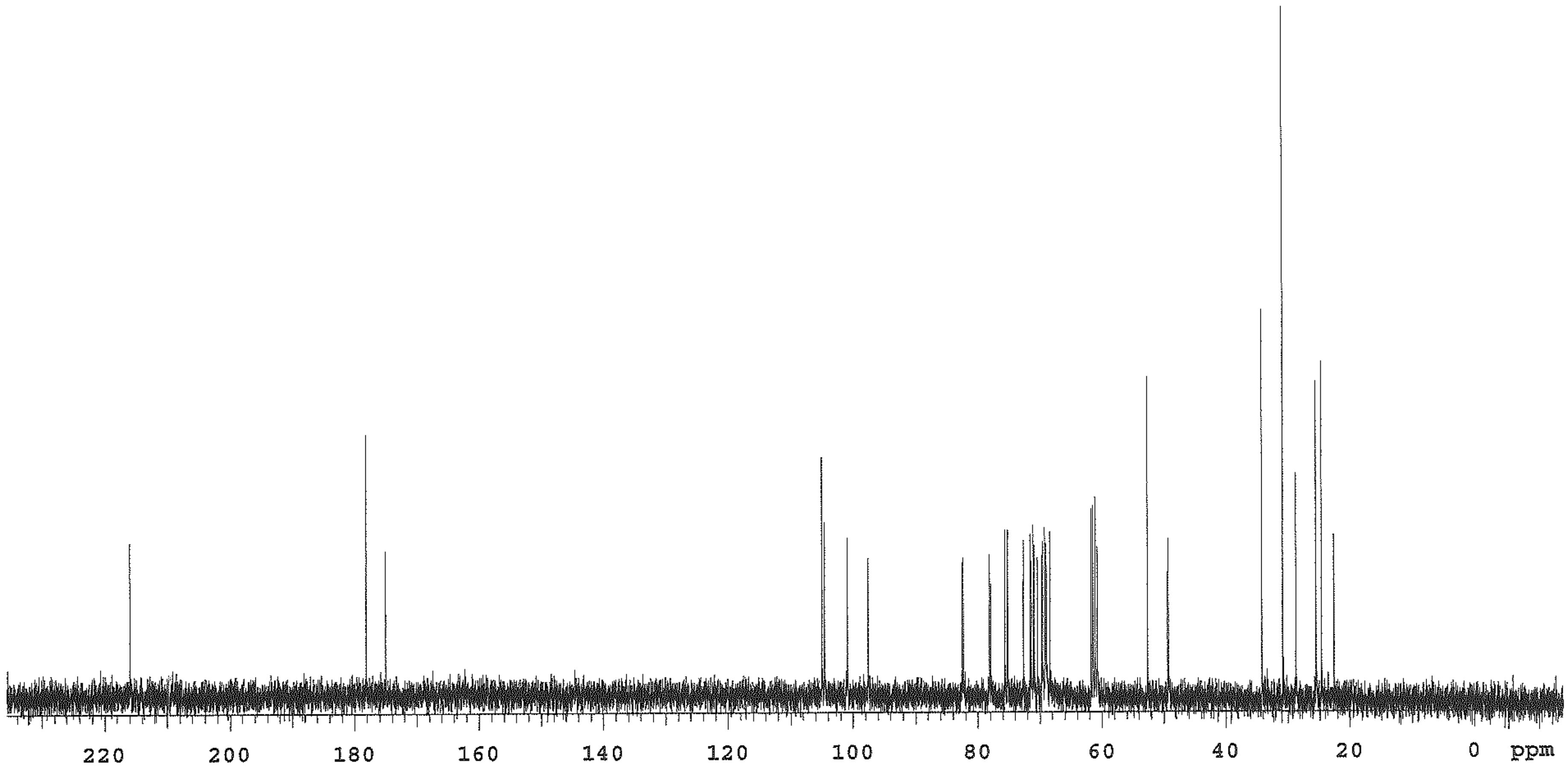

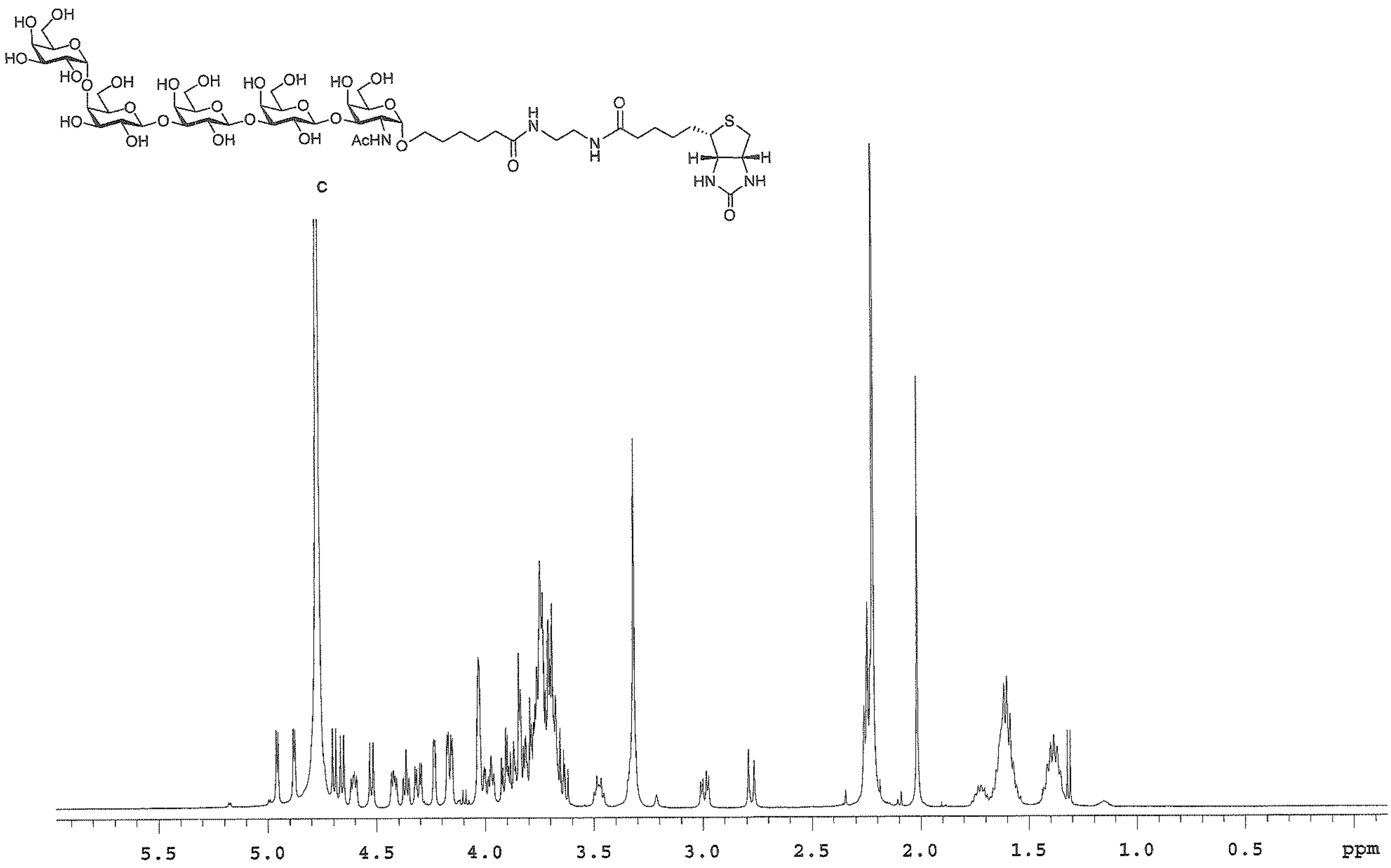

C
5.5
5.0
4.5
4.0
3.5
3.0
2.5
2.0
1.5
1.0
0.5
ppm

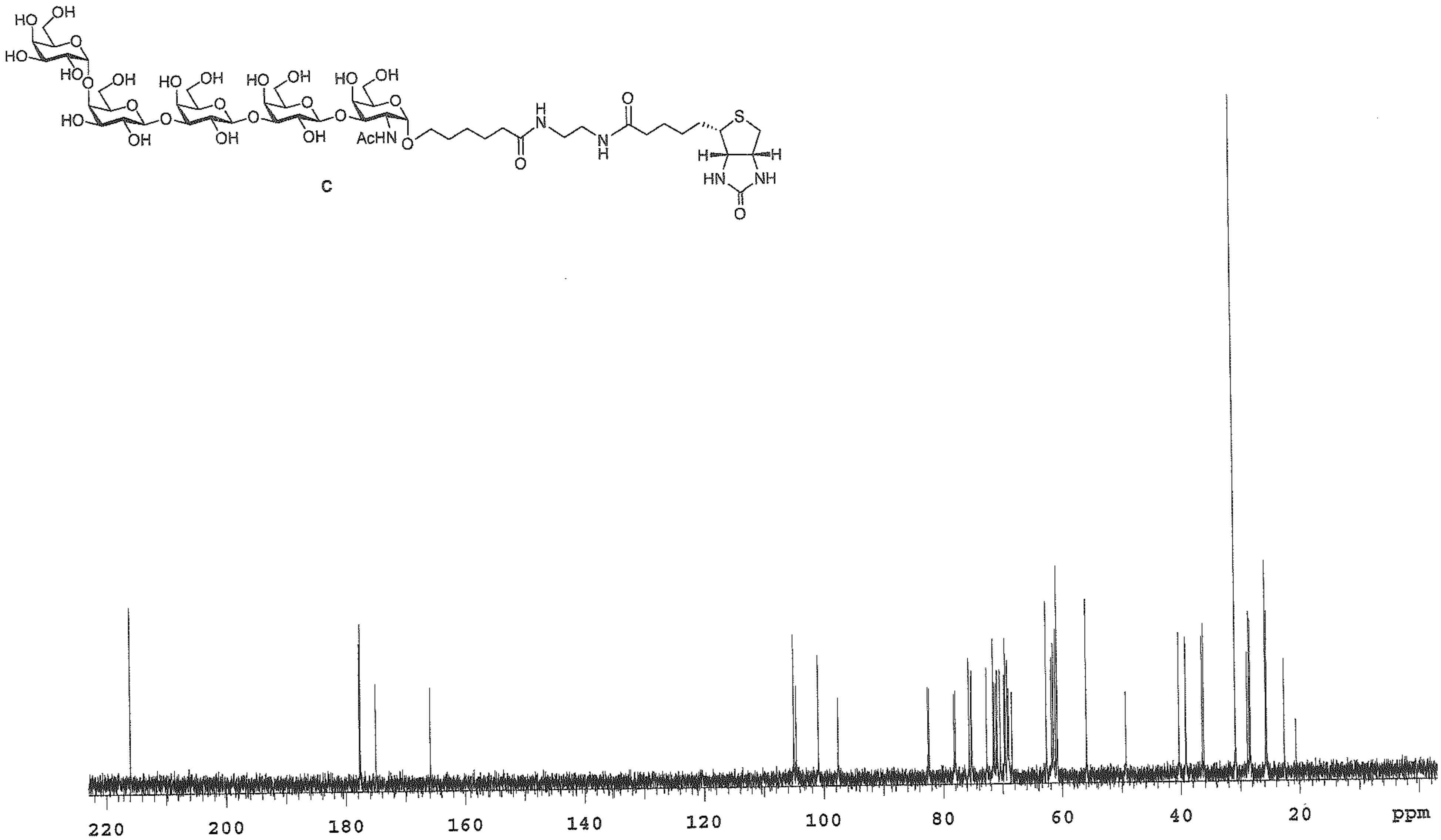
HO
OH
O
HO
HO
HO
OH
HO
OH
O
HO
HO
OH
O
HO
OH
O
HO
OH
O
HO
OH
O
AcHN
O
H
N
N
H
O
O
S
H
H
HN
NH
O
C
220
200
180
160
140
120
100
80
60
40
20
ppm

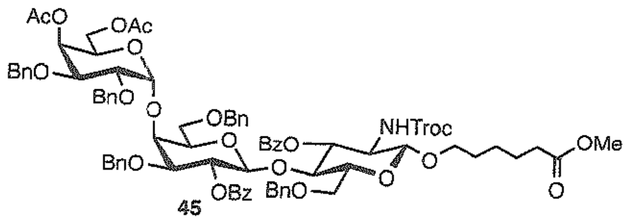
AcO
OAc
BnO
BnO
OBn
BzO
NHTroc
OMe
BnO
OBz
BnO
O

45

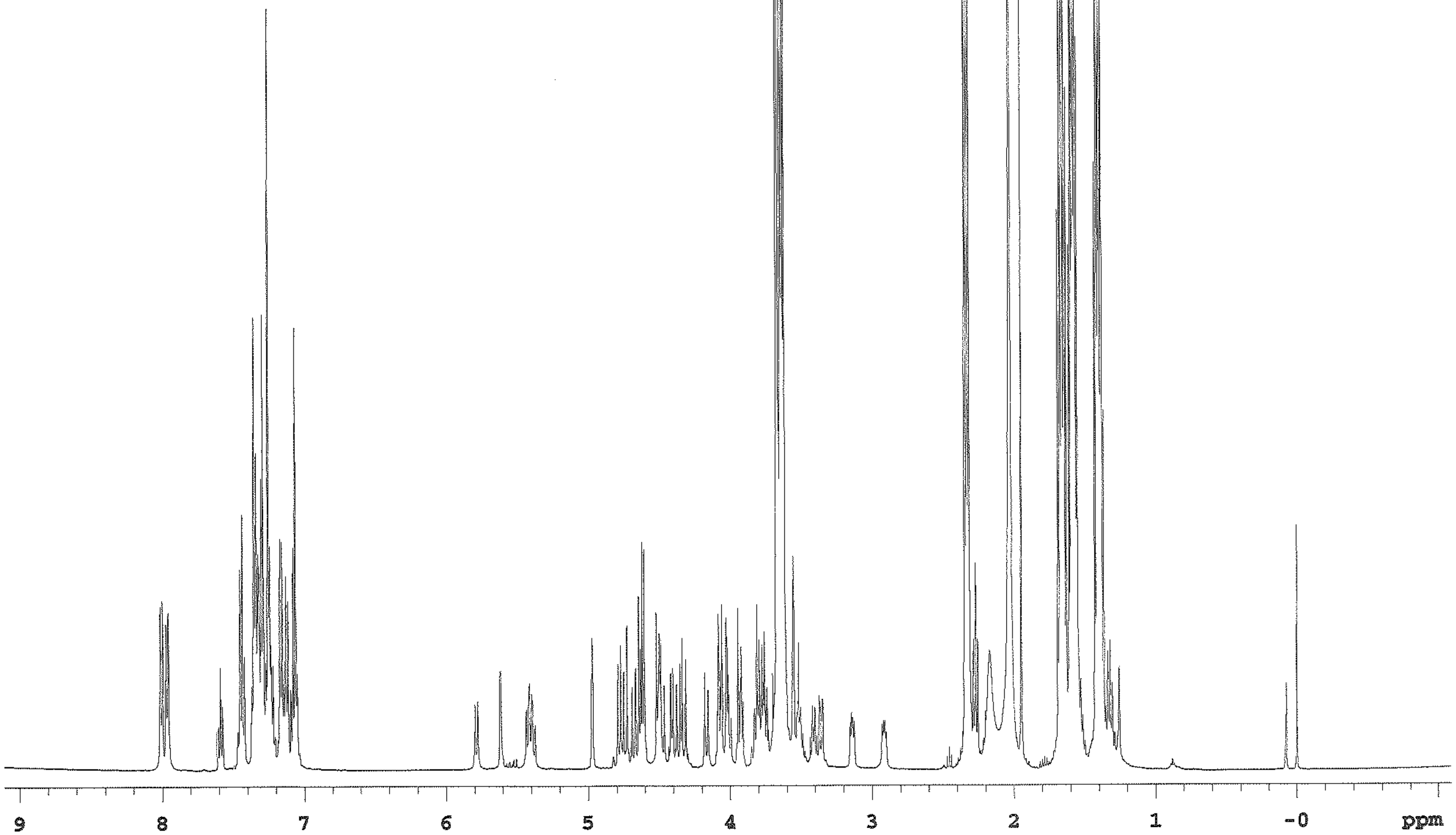
9
8
7
6
5
4
3
2
1
-0
ppm

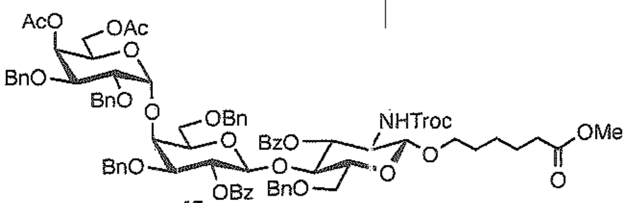

45

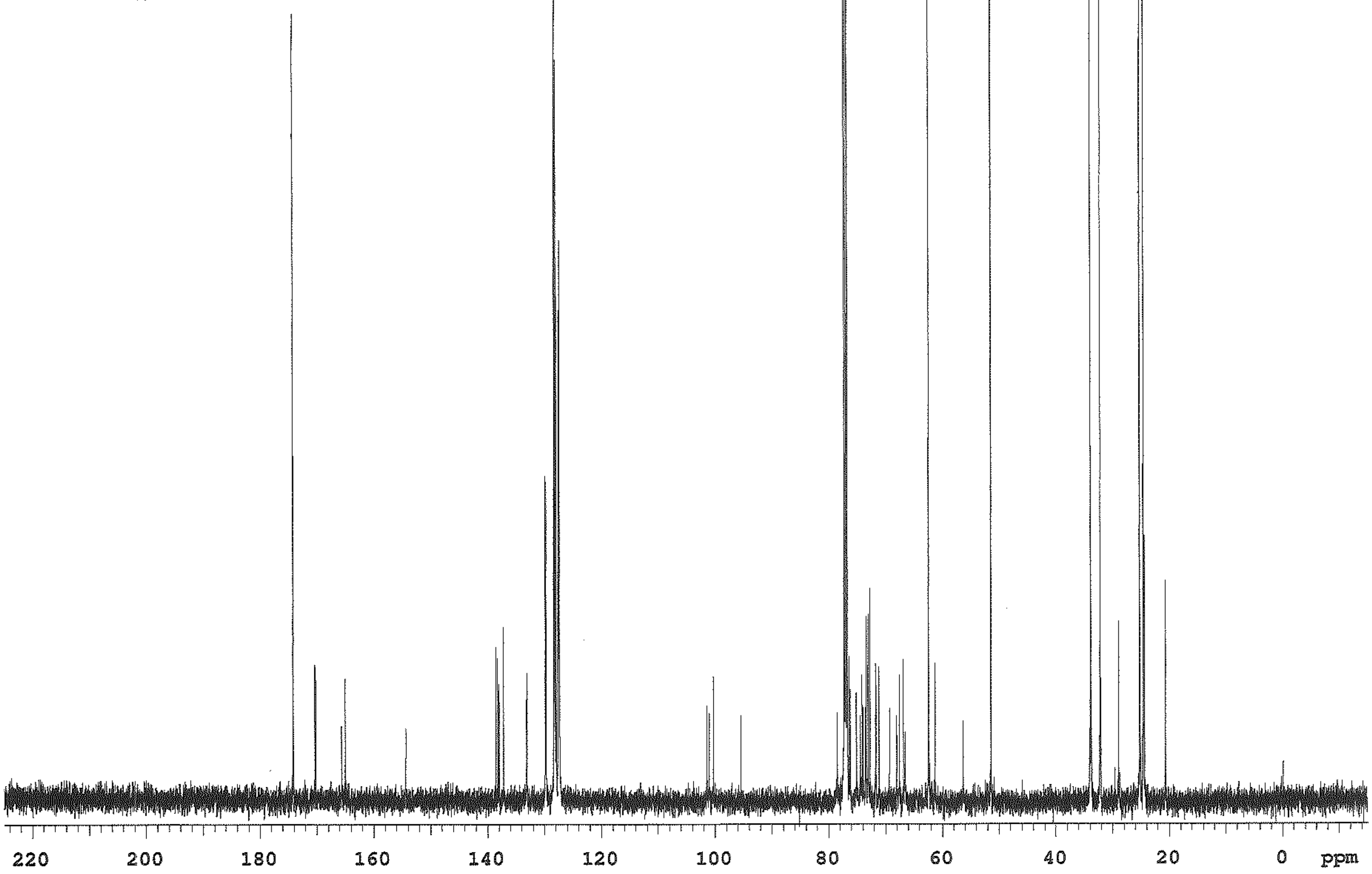

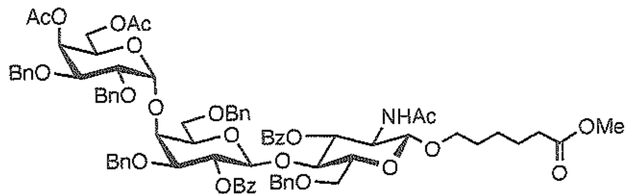

46

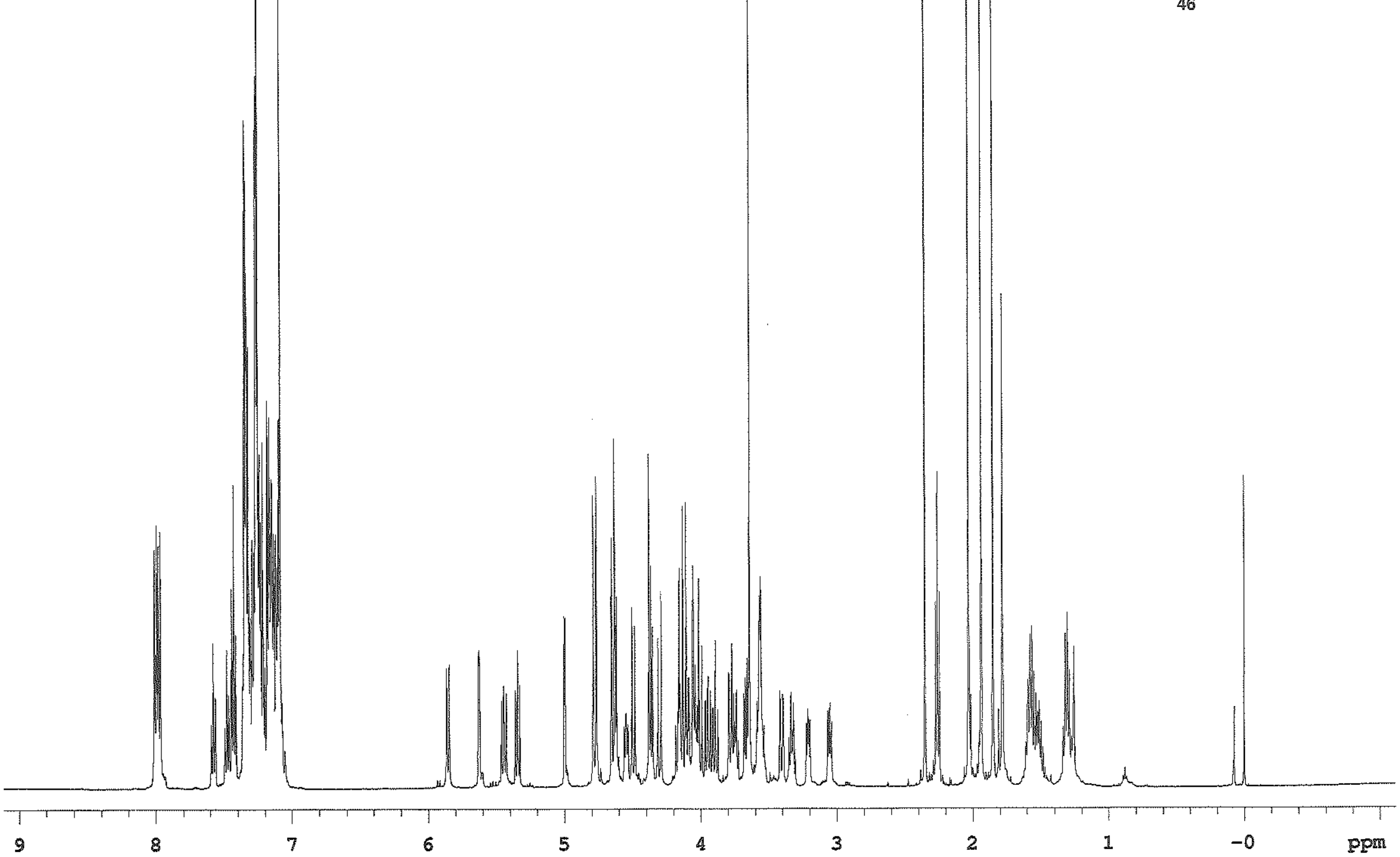

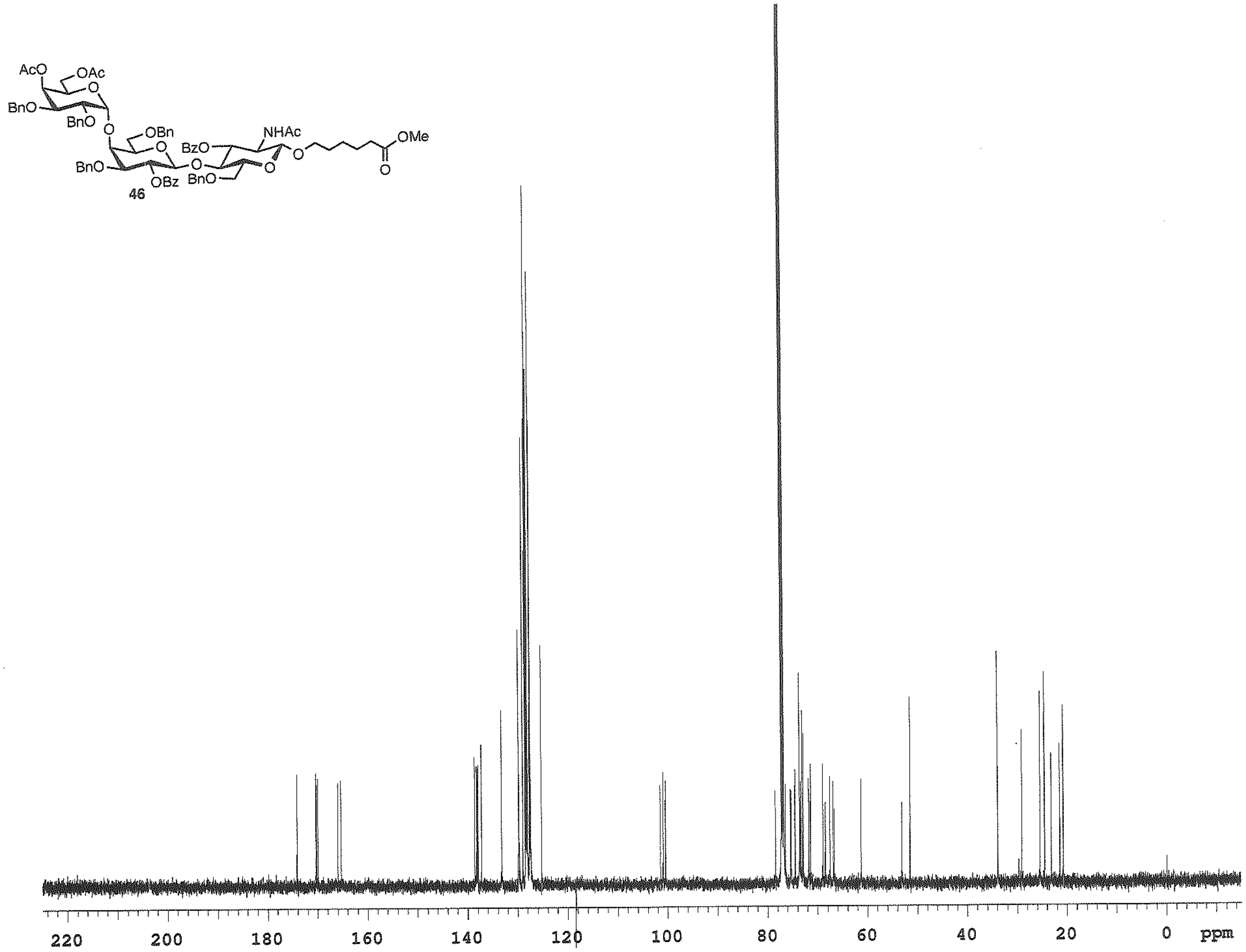

AcO
OAc
BnO
BnO
OBn
BzO
NHAc
OMe
BnO
OBz
BnO

46

220
200
180
160
140
120
100
80
60
40
20
0
ppm

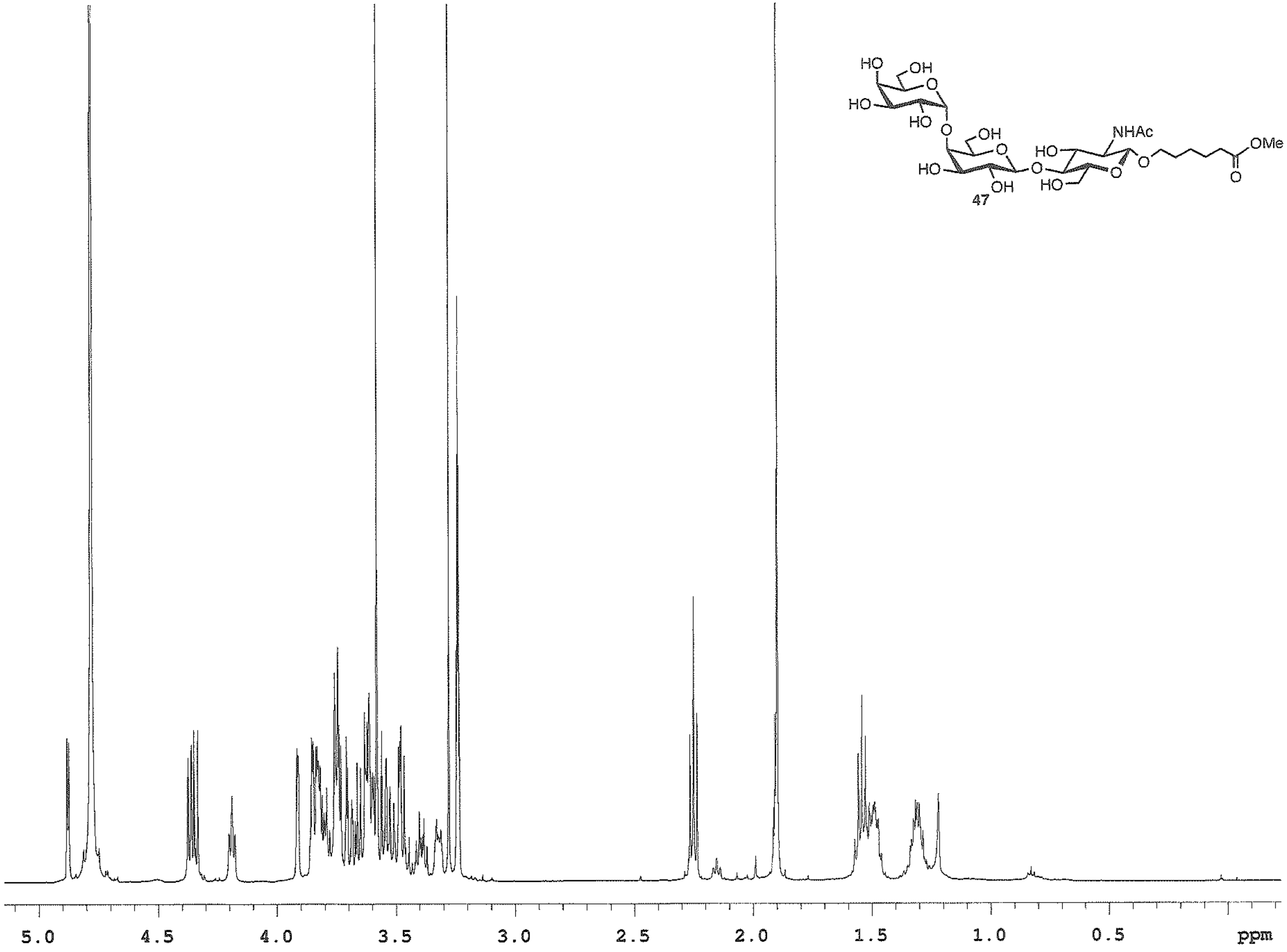

47
5.0
4.5
4.0
3.5
3.0
2.5
2.0
1.5
1.0
0.5
ppm

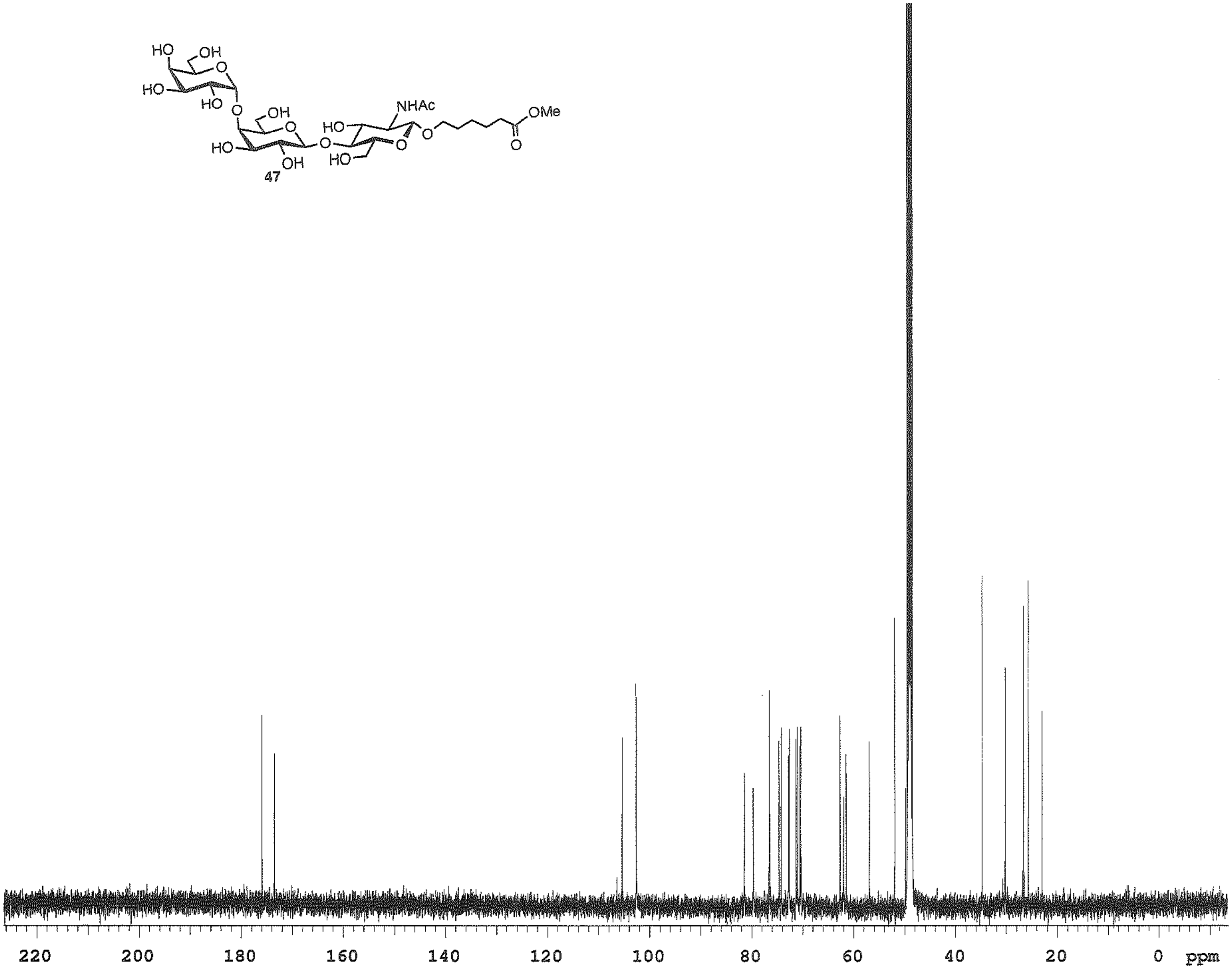

HO
OH
HO
HO
OH
NHAc
OMe
HO
HO
HO
O
OH
47
220
200
180
160
140
120
100
80
60
40
20
0
ppm

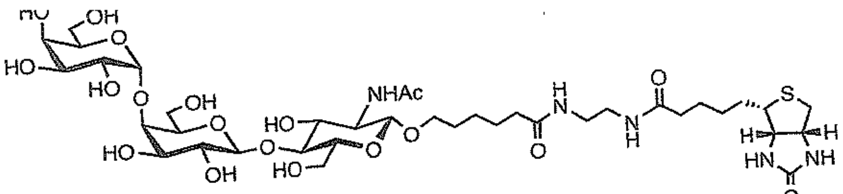

D

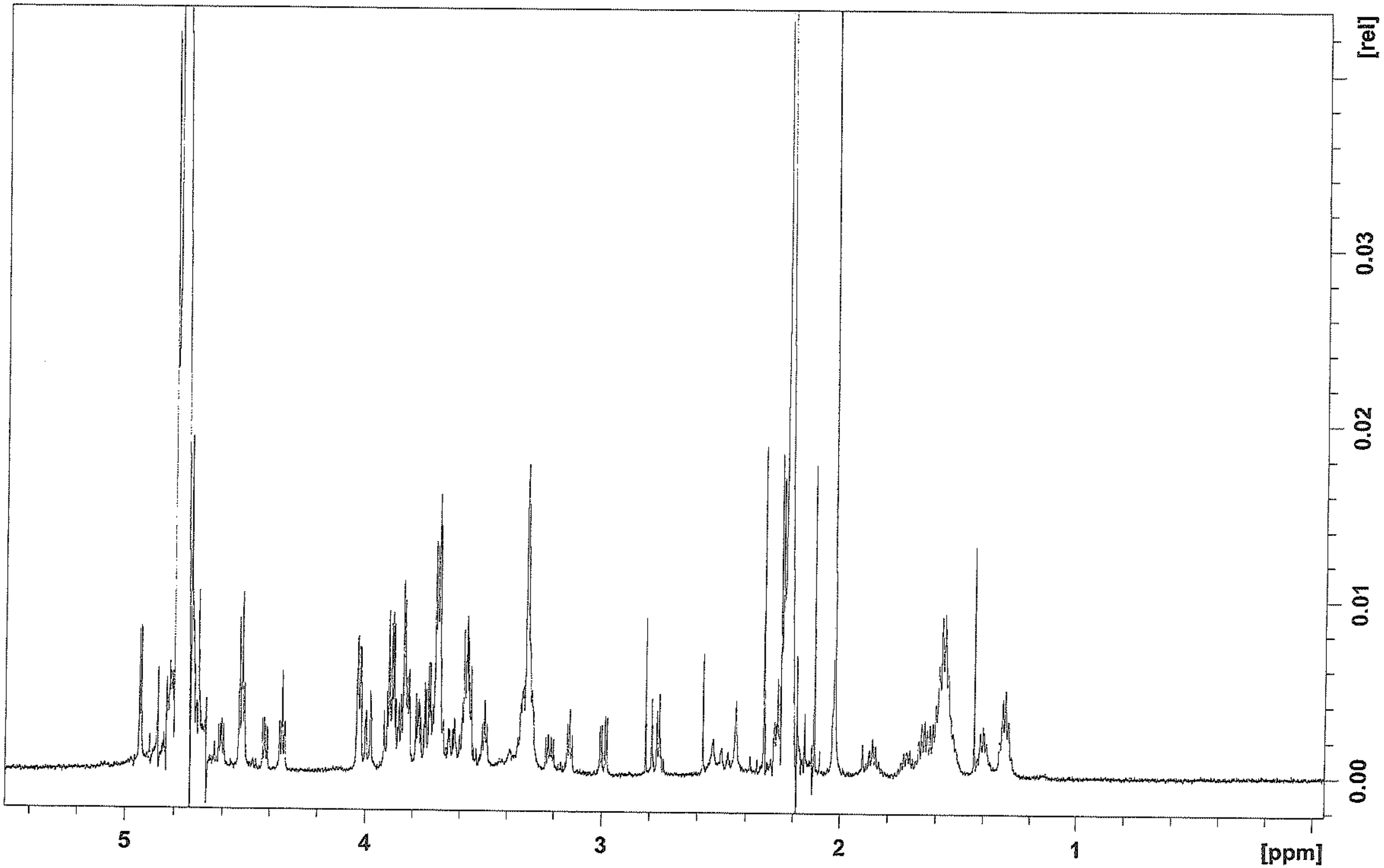

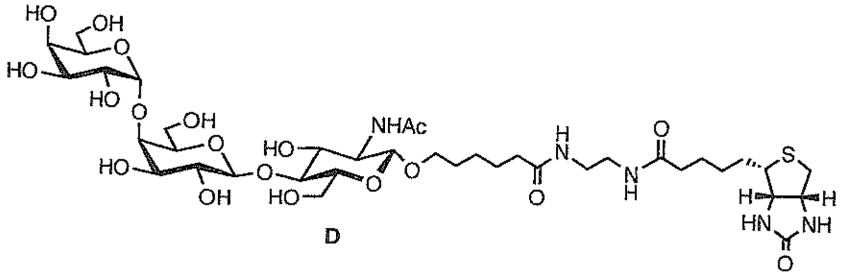

D

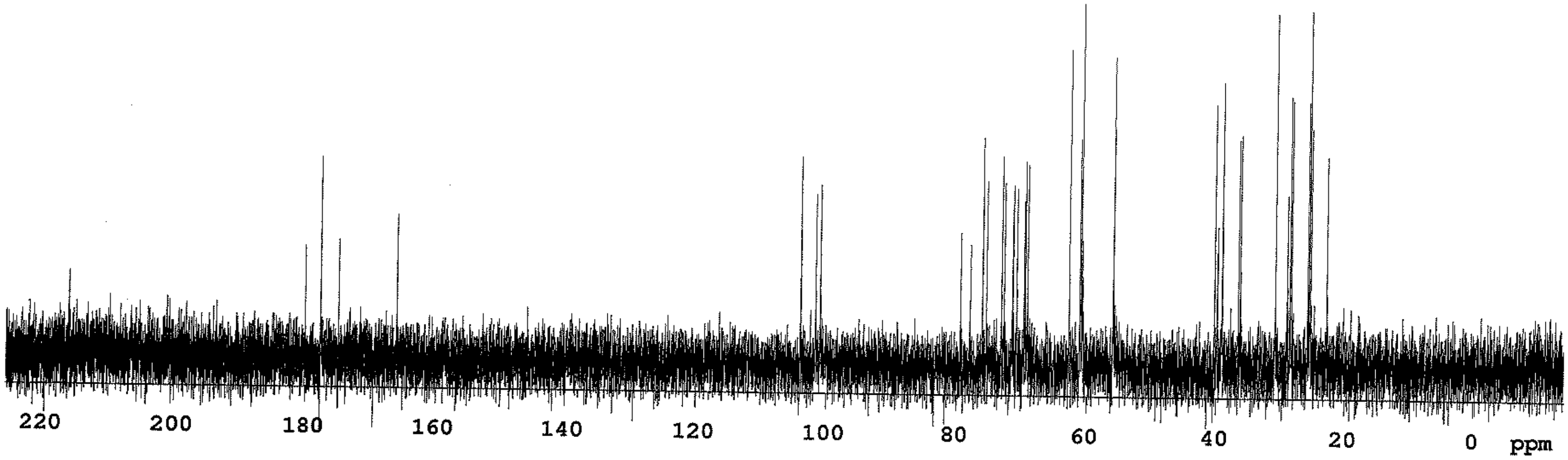

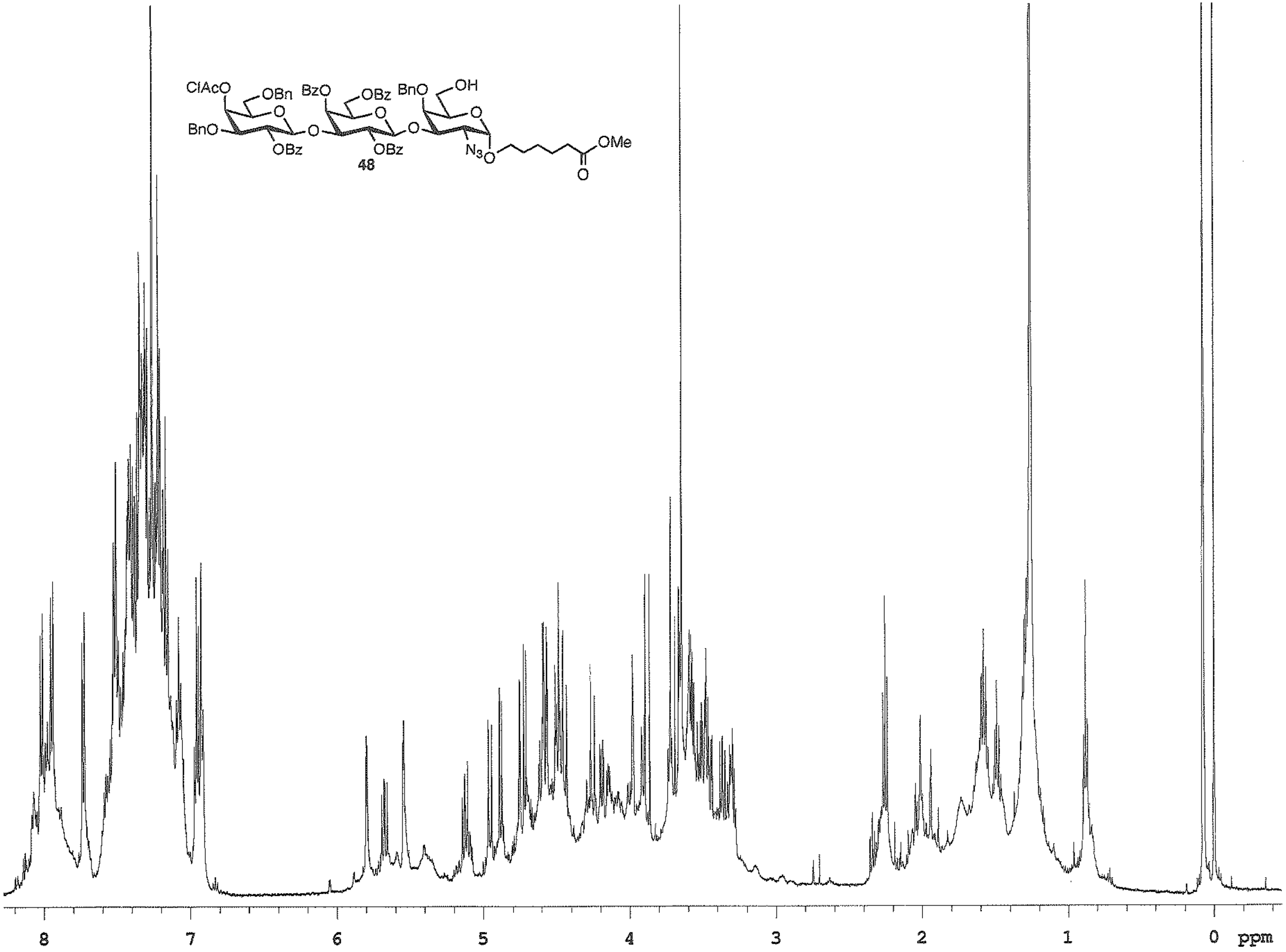

48

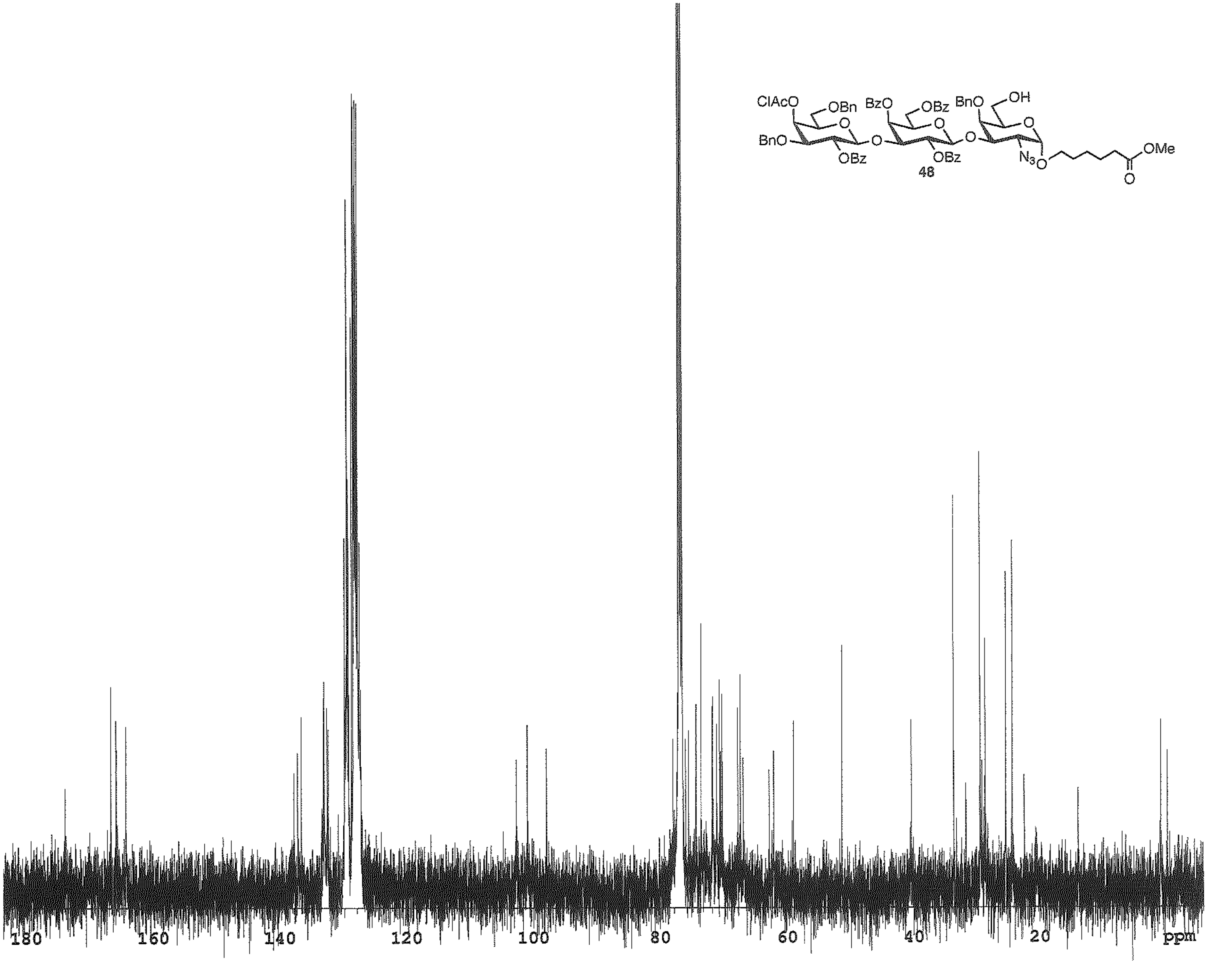
ClAcO
OBn
BnO
OBz
BzO
OBz
OBz
48
BnO
OH
N3
OMe
180
160
140
120
100
80
60
40
20
ppm

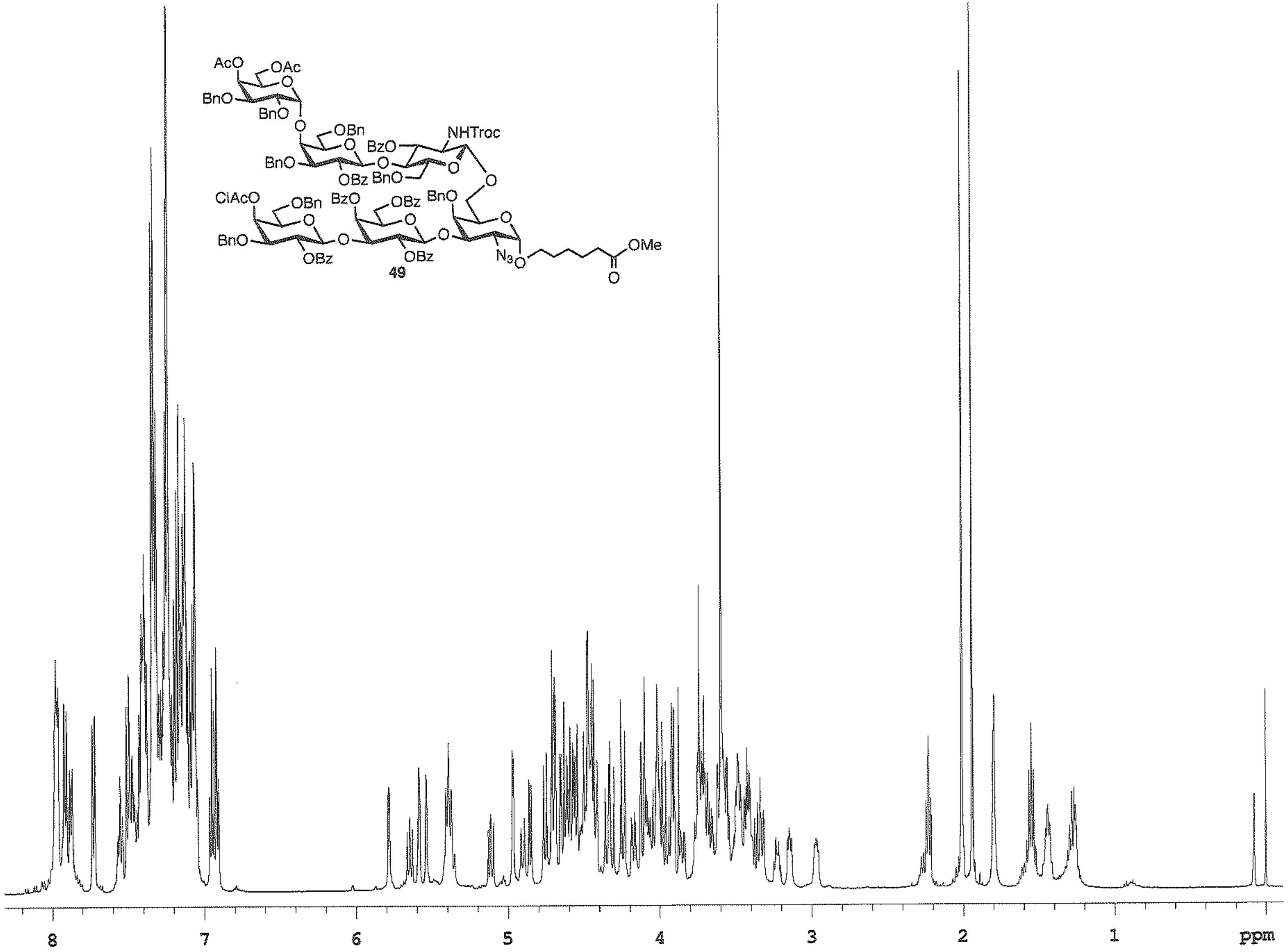
AcO
OAc
BnO
BnO
OBn
BzO
NHTroc
BnO
OBz
BnO
ClAcO
OBn
BzO
OBz
BnO
BnO
OBz
OBz
N3
OMe
O
49
8
7
6
5
4
3
2
1
ppm

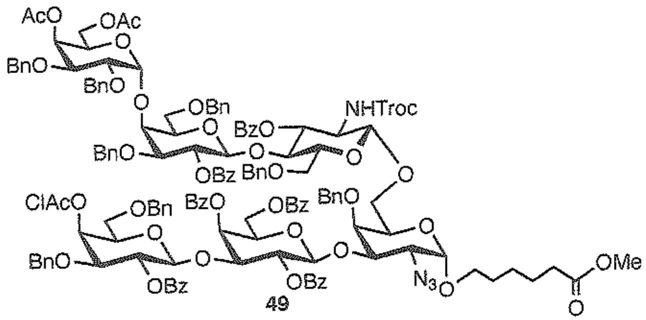

49

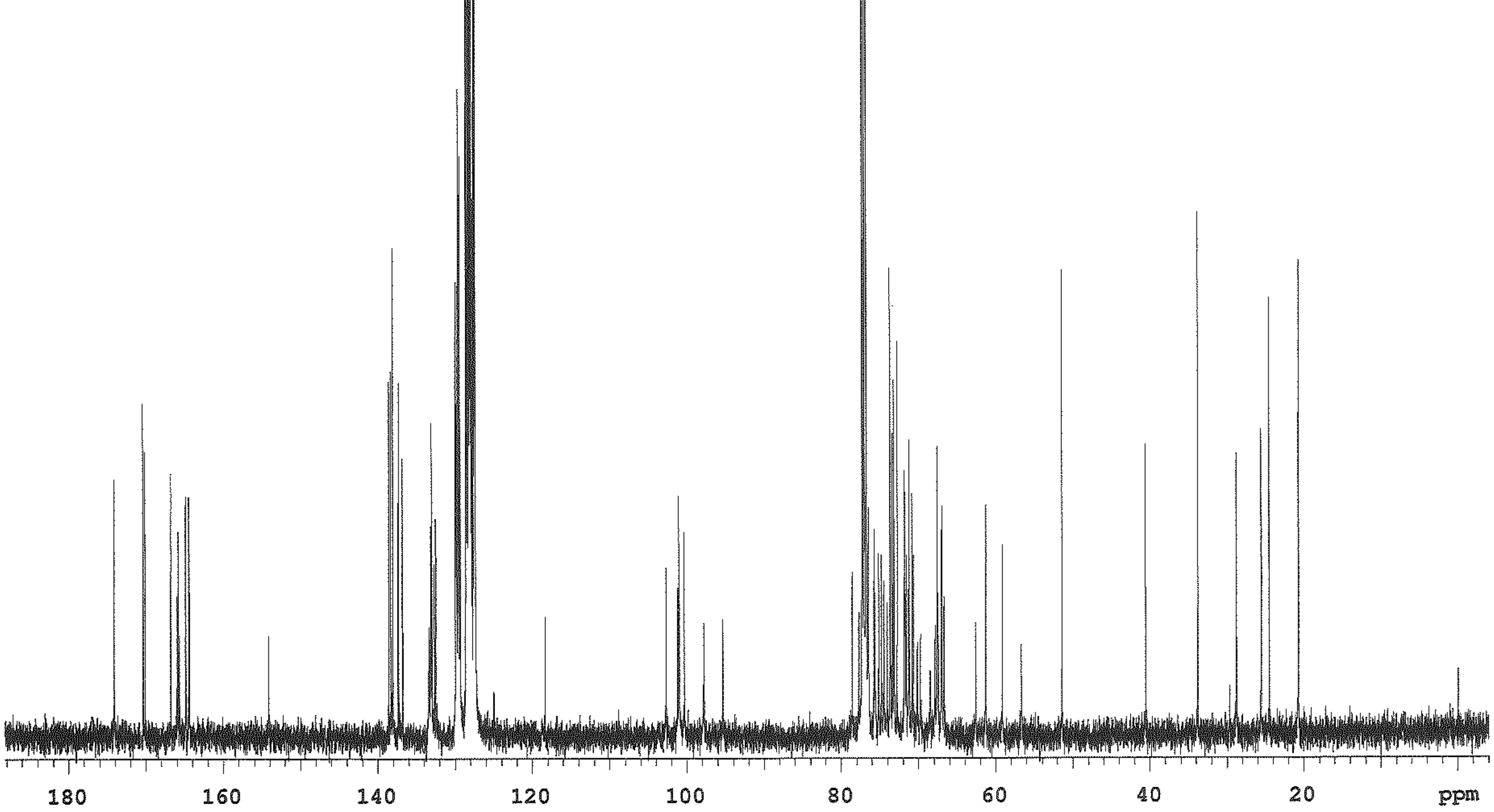

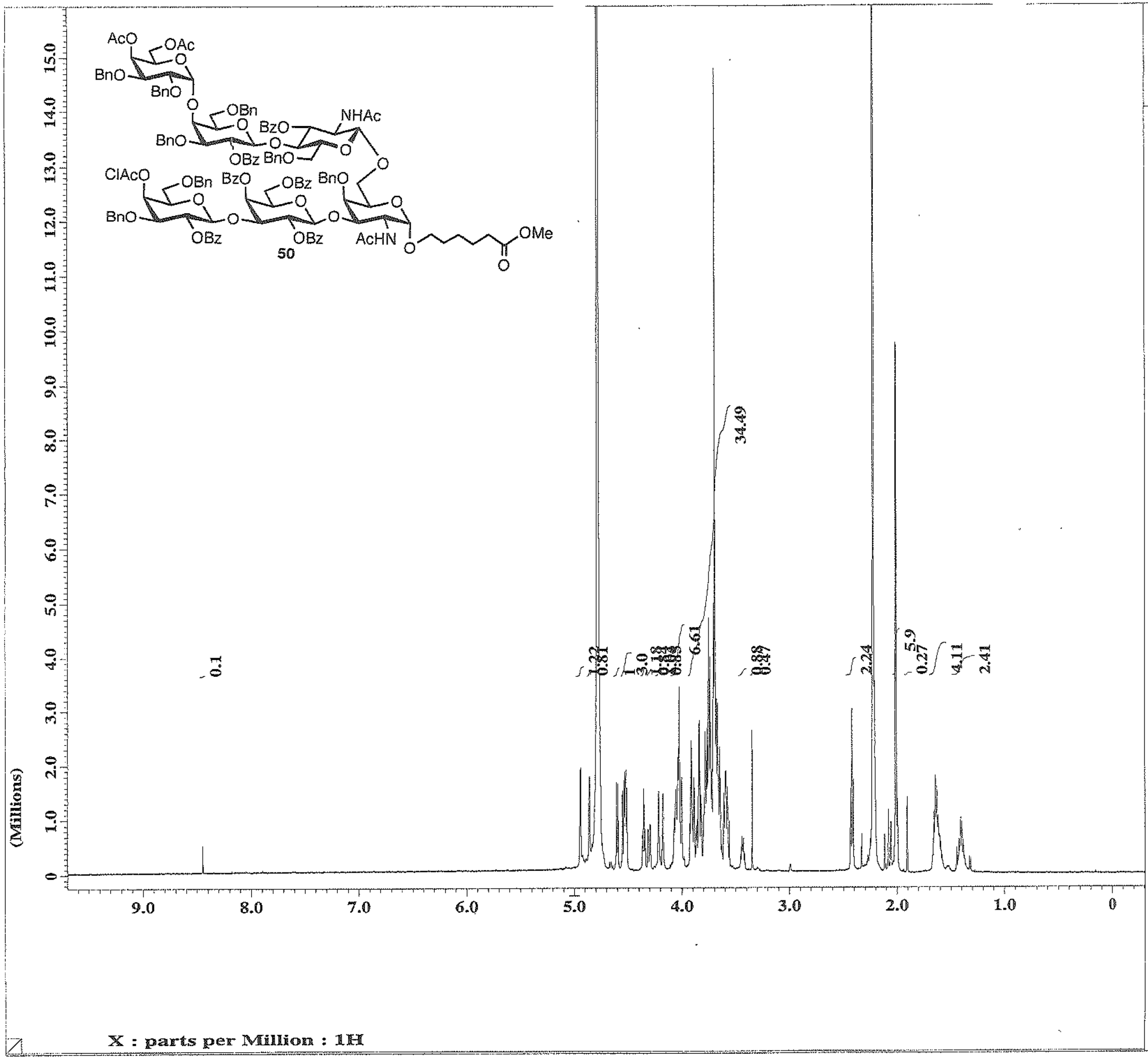

JEOL

```
---- ACQUISITION PARAMETERS ----
File Name             = JY-6FREE-H.3
Author                = JEOL LTD.
Sample ID             = aaa
Content               = 6-free
Creation Date         =  9-OCT-2013 06:27:48

Revision Date         = 10-OCT-2013 12:29:36
Spec Site             = ECP600

Spec Type             = DELTA_NMR
Data Format           = 1D COMPLEX
Dimensions            = X
Dim Title             = 1H
Dim Size              = 32768
Dim Units             = [ppm]
Actual_start_time     =  9-OCT-2013 06:25:56
Delay_of_start        = 1[s]
Digital_filter        = FALSE
End_time              =  9-OCT-2013 06:27:49
Experiment            = single_pulse.exp
Field_strength        = 14.09636928[T]
Filter_mode           = BUTTERWORTH
Filter_width          = 4.50129228[kHz]
Irr_code              = 210
Irr_noise             = WALTZ
Irr_pwidth            = 50[us]
Iterations            = 0
Local_time            =  9-OCT-2013 06:27:47
Obs_noise             = WAUGH
Obs_pwidth            = 1[us]
Probe_id              = 2692
Recvr_gain            = 19
Relaxation_delay      = 3[s]
Scans                 = 16
Solvent               = D2O
Spin_get              = 15[Hz]
Spin_lock_90          = 34[us]
Spin_lock_attn        = 10.4[dB]
Temp_get              = 24.4[dC]
X90                   = 11.2[us]
X_acq_duration        = 3.637248[s]
X_angle               = 45[deg]
X_domain              = 1H
X_freq                = 600.17530548[MHz]
X_offset              = 5[ppm]
X_points              = 32768
X_prescans            = 0
X_pulse               = 5.6[us]
X_resolution          = 0.27494153[Hz]
X_sweep               = 9.00900901[kHz]
Tri90                 = 10[us]
Tri_noise             = WALTZ
Tri_pwidth            = 1[us]
Qua90                 = 10[us]
Qua_noise             = WAUGH
Qua_pwidth            = 1[us]
```

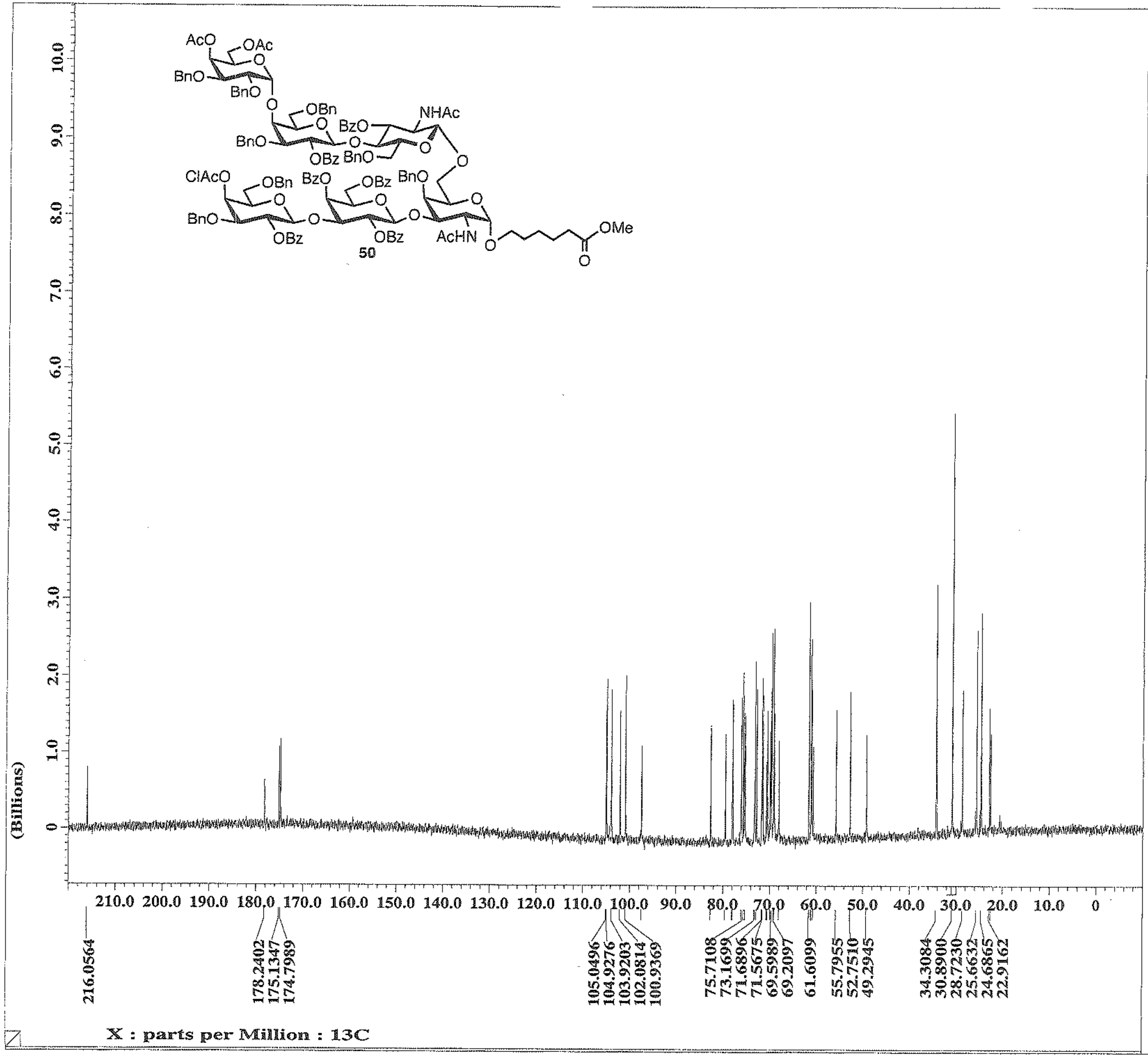

JEOL

---- ACQUISITION PARAMETERS ----

File Name	= JY-6FREE-C.4
Author	= JEOL LTD.
Sample ID	= aaa
Content	= 6-free
Creation Date	= 10-OCT-2013 08:56:33
Revision Date	= 11-OCT-2013 12:39:07
Spec Site	= ECP600
Spec Type	= DELTA_NMR
Data Format	= 1D COMPLEX
Dimensions	= X
Dim Title	= 13C
Dim Size	= 32768
Dim Units	= [ppm]
Actual_start_time	= 10-OCT-2013 08:56:33
Delay_of_start	= 1[s]
Digital_filter	= FALSE
End_time	= 10-OCT-2013 08:56:40
Experiment	= single_pulse_dec
Field_strength	= 14.09636928[T]
Filter_mode	= BUTTERWORTH
Filter_width	= 18.8641788[kHz]
Irr_code	= 210
Irr_domain	= 1H
Irr_freq	= 600.17530548[MHz]
Irr_noise	= WALTZ
Irr_offset	= 5.0[ppm]
Irr_pwidth	= 50[us]
Iterations	= 0
Local_time	= 10-OCT-2013 08:56:33
Obs_noise	= WAUGH
Obs_pwidth	= 1[us]
Probe_id	= 2692
Recvr_gain	= 30
Relaxation_delay	= 1.5[s]
Scans	= 40000
Solvent	= D2O
Spin_get	= 15[Hz]
Spin_lock_90	= 34[us]
Spin_lock_attn	= 10.4[dB]
Temp_get	= 25.8[dC]
X90	= 12.3[us]
X_acq_duration	= 0.868352[s]
X_angle	= 90[deg]
X_domain	= 13C
X_freq	= 150.92852325[MHz]
X_offset	= 100[ppm]
X_points	= 32768
X_prescans	= 2
X_pulse	= 12.3[us]
X_resolution	= 1.15164187[Hz]
X_sweep	= 37.73584906[kHz]
Tri90	= 10[us]
Tri_noise	= WALTZ
Tri_pwidth	= 1[us]
Qua90	= 10[us]
Qua_noise	= WAUGH
Qua_pwidth	= 1[us]

51

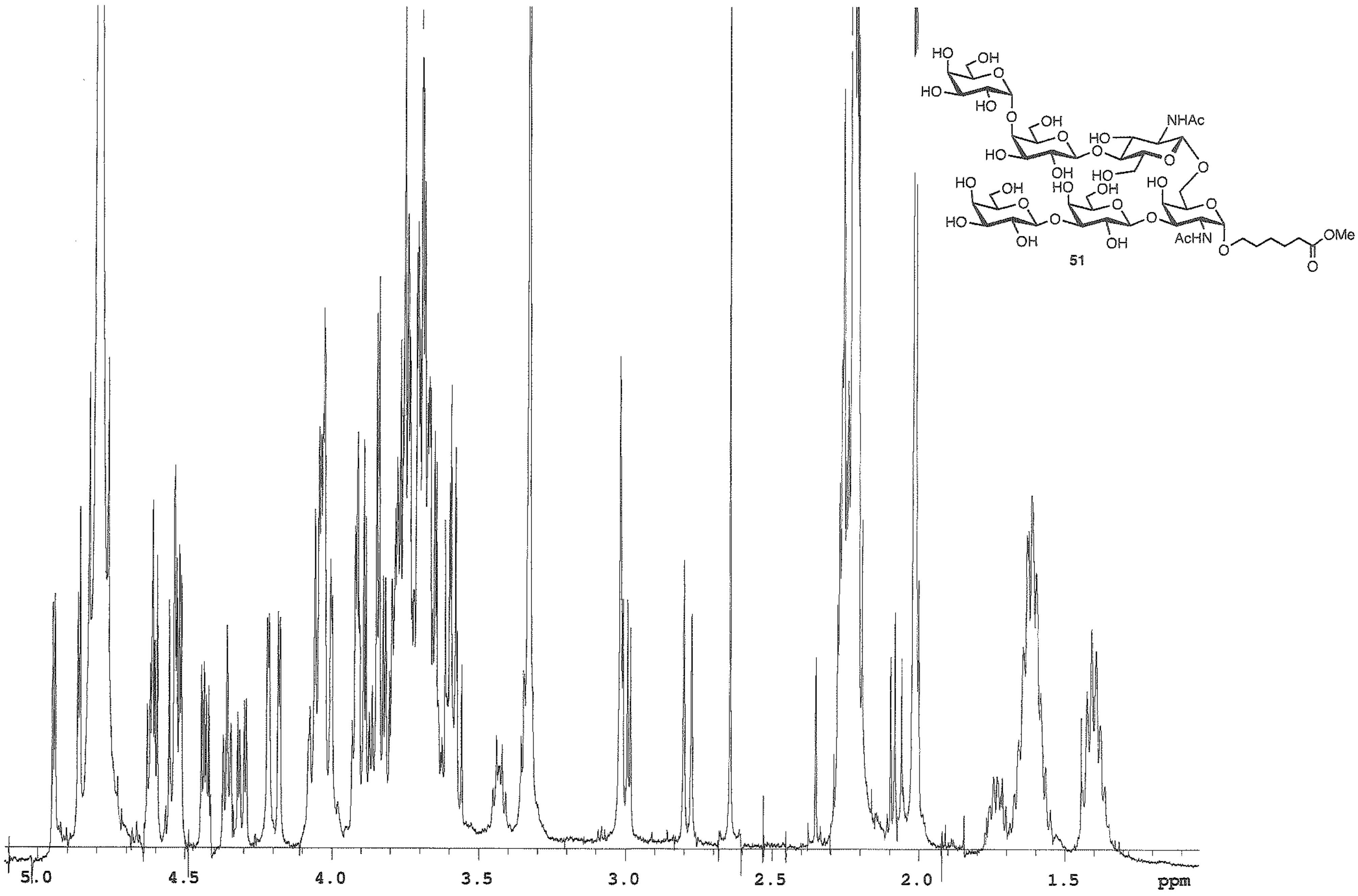

51

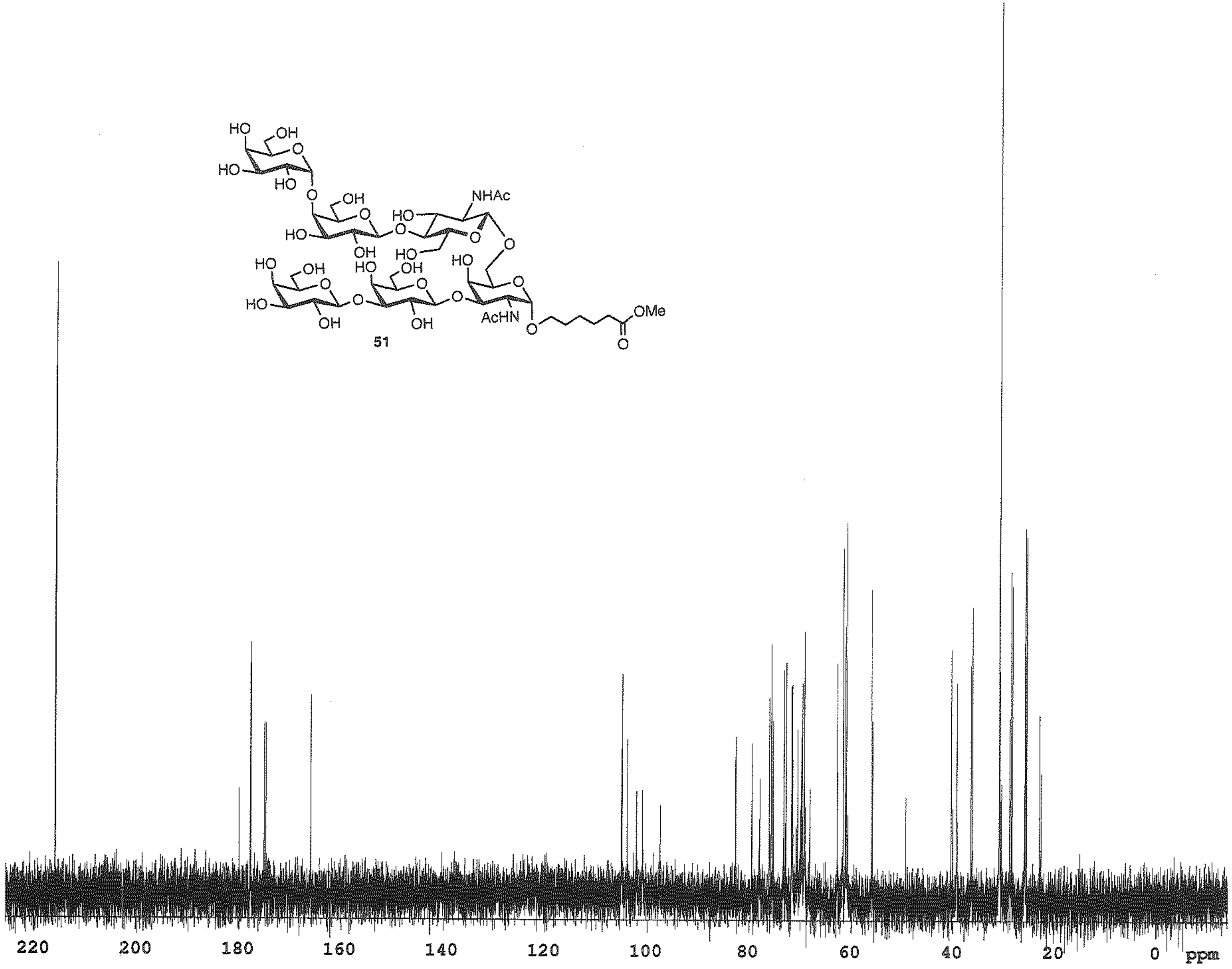
HO
OH
O
HO
HO
NHAc
HO
OMe
AcHN

51

220
200
180
160
140
120
100
80
60
40
20
0
ppm

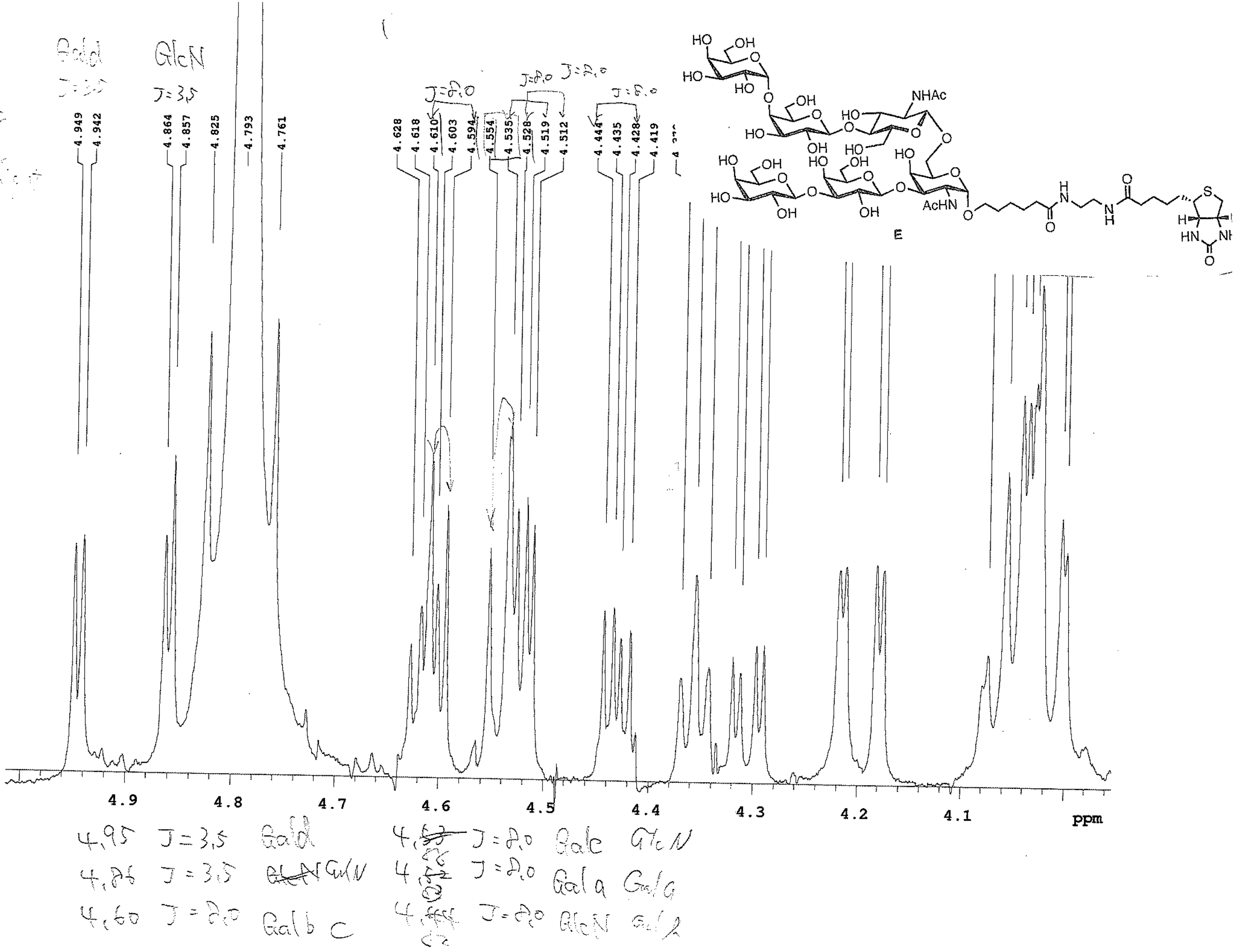

E
Galα J=3.5
GlcN J=3.5
4.949
4.942
4.864
4.857
4.825
4.793
4.761
4.628
4.618
4.610
4.603
4.594
4.554
4.535
4.528
4.519
4.512
4.444
4.435
4.428
4.419
J=8.0
NHAc
AcHN
HO
OH
E
ppm
4.9
4.8
4.7
4.6
4.5
4.4
4.3
4.2
4.1
4.95 J=3.5 Galα
4.86 J=3.5 GalN
4.60 J=8.0 Galb c
4.[illegible] J=8.0 Galc GlcN
4.[illegible] J=8.0 Gala Gala
4.[illegible] J=8.0 GlcN

INDEX	FREQUENCY	PPM	HEIGHT	INDEX	FREQUENCY	PPM	HEIGHT	INDEX	FREQUENCY	PPM	HEIGHT	INDEX	FREQUENCY	PPM	HEIGHT
1	2475.7	4.949	46.5	42	1960.9	3.920	62.8	83	1819.9	3.638	25.9	124	1105.5	2.210	188.3
2	2471.8	4.942	48.4	43	1957.8	3.914	75.8	84	1815.2	3.629	15.1	125	1104.7	2.208	183.2
3	2433.0	4.864	48.6	44	1956.7	3.912	82.5	85	1812.7	3.624	18.2	126	1103.9	2.207	142.6
4	2429.3	4.857	66.7	45	1954.5	3.907	46.9	86	1807.9	3.614	64.1	127	1103.1	2.205	166.5
5	2413.3	4.825	94.8	46	1953.3	3.905	41.9	87	1805.7	3.610	27.4	128	1102.5	2.204	161.6
6	2397.2	4.793	16632.8	47	1945.9	3.890	80.9	88	1803.7	3.606	26.4	129	1096.9	2.193	64.4
7	2381.2	4.761	98.0	48	1942.7	3.884	64.8	89	1800.3	3.599	71.9	130	1048.5	2.096	35.0
8	2314.8	4.628	24.9	49	1936.9	3.872	23.4	90	1798.0	3.594	92.4	131	1047.9	2.095	35.4
9	2309.9	4.618	33.7	50	1931.8	3.862	29.1	91	1790.2	3.579	79.3	132	1040.9	2.081	44.7
10	2305.7	4.610	68.1	51	1924.3	3.847	107.5	92	1787.7	3.574	40.7	133	1029.7	2.059	35.0
11	2302.5	4.603	38.6	52	1920.3	3.839	115.3	93	1779.7	3.558	33.5	134	1009.0	2.017	409.3
12	2298.1	4.594	56.5	53	1913.6	3.826	52.3	94	1720.5	3.440	18.6	135	1006.0	2.011	417.3
13	2277.7	4.554	47.1	54	1909.7	3.818	51.9	95	1710.7	3.420	16.7	136	1000.7	2.001	51.5
14	2268.2	4.535	75.4	55	1902.5	3.804	32.2	96	1678.1	3.355	18.6	137	829.5	1.658	17.9
15	2264.7	4.528	56.0	56	1898.6	3.796	51.6	97	1674.5	3.348	32.4	138	822.4	1.644	37.6
16	2260.3	4.519	58.7	57	1895.4	3.789	46.1	98	1672.7	3.344	31.6	139	820.4	1.640	36.4
17	2256.9	4.512	52.4	58	1892.9	3.784	66.5	99	1664.6	3.328	433.9	140	815.4	1.630	61.1
18	2222.8	4.444	33.3	59	1889.5	3.777	77.2	100	1659.0	3.317	29.0	141	812.6	1.625	62.0
19	2218.3	4.435	34.0	60	1887.7	3.774	67.8	101	1509.8	3.018	98.6	142	807.8	1.615	69.5
20	2214.9	4.428	27.0	61	1883.7	3.766	101.9	102	1505.1	3.009	47.4	143	799.7	1.599	53.1
21	2210.3	4.419	28.9	62	1880.6	3.760	87.5	103	1497.0	2.993	47.3	144	791.9	1.583	27.7
22	2185.8	4.370	18.5	63	1876.1	3.751	181.1	104	1492.0	2.983	41.5	145	722.5	1.444	22.4
23	2179.2	4.357	41.8	64	1871.6	3.742	128.5	105	1400.8	2.801	55.8	146	712.5	1.424	28.1
24	2172.5	4.343	21.0	65	1870.5	3.739	116.9	106	1387.7	2.774	44.4	147	705.1	1.410	41.5
25	2160.9	4.320	23.3	66	1868.1	3.735	102.5	107	1322.2	2.643	452.1	148	697.1	1.394	36.7
26	2157.1	4.313	19.7	67	1864.4	3.727	47.5	108	1176.3	2.352	31.5	149	690.0	1.379	21.0
27	2150.0	4.298	25.8	68	1862.1	3.723	49.4	109	1175.6	2.350	35.4				
28	2146.2	4.291	26.1	69	1860.8	3.720	49.4	110	1145.0	2.289	14.9				
29	2110.3	4.219	43.4	70	1856.6	3.712	120.3	111	1138.9	2.277	46.4				
30	2107.3	4.213	44.3	71	1854.4	3.707	126.9	112	1135.3	2.270	72.1				
31	2092.2	4.183	44.8	72	1850.9	3.700	104.2	113	1131.3	2.262	98.0				
32	2088.6	4.176	43.5	73	1847.6	3.694	171.6	114	1129.0	2.257	148.0				
33	2038.1	4.075	24.5	74	1846.2	3.691	163.3	115	1128.0	2.255	155.0				
34	2029.2	4.057	66.3	75	1842.8	3.684	135.4	116	1124.0	2.247	76.6				
35	2022.4	4.043	83.6	76	1837.6	3.674	88.6	117	1120.7	2.241	93.4				
36	2019.2	4.037	82.1	77	1836.1	3.671	91.9	118	1112.9	2.225	15056.6				
37	2016.0	4.030	86.4	78	1834.8	3.668	94.2	119	1109.8	2.219	279.2				
38	2014.0	4.026	108.5	79	1833.0	3.664	91.8	120	1108.3	2.216	171.6				
39	2002.9	4.004	55.8	80	1829.4	3.657	45.3	121	1107.6	2.214	208.7				
40	2000.4	3.999	48.2	81	1826.1	3.651	82.9	122	1106.9	2.213	195.0				
41	1966.0	3.930	21.7	82	1822.9	3.644	76.3	123	1106.1	2.211	162.0				

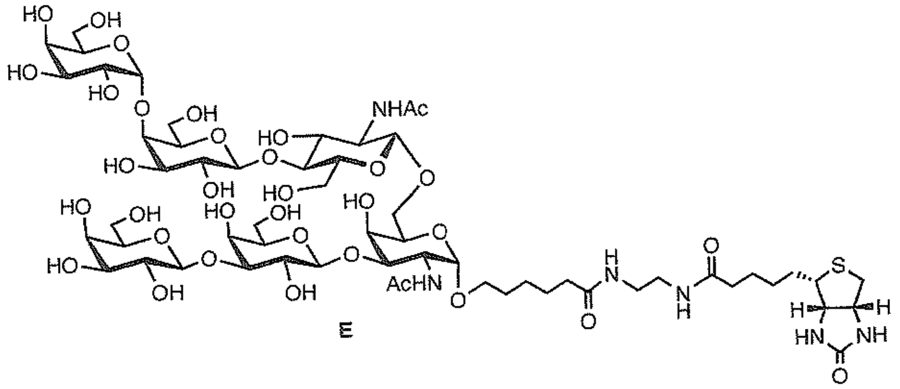

E

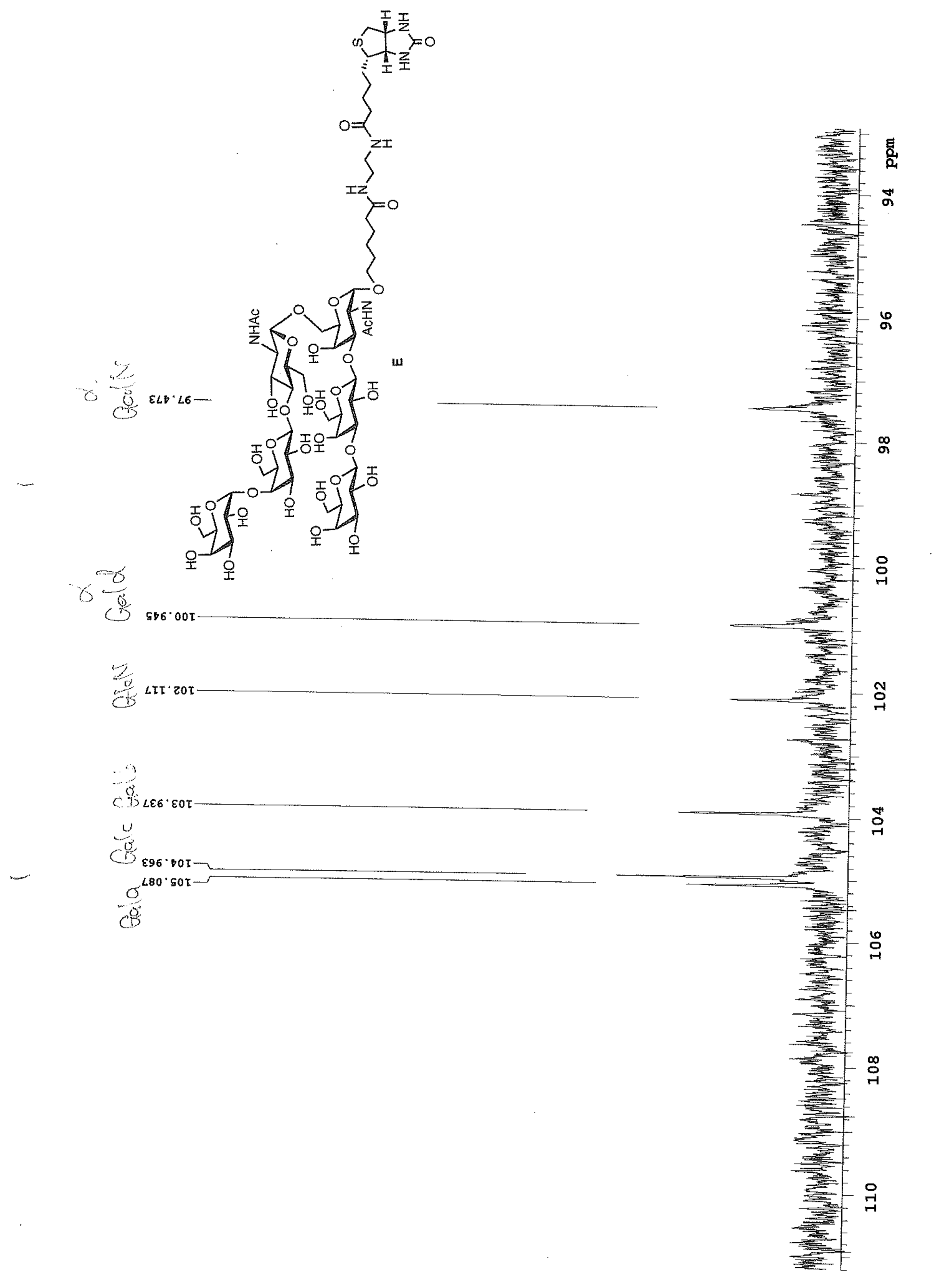
E
97.473
100.945
102.117
103.937
104.963
105.087
94
96
98
100
102
104
106
108
110
ppm

Sample ID: s_20131022_01
File: s_20131022_01/data/Carbon_01.fid

Pulse Sequence: s2pul

Solvent: d2o
Ambient temperature
Operator: walkup
File: Carbon_01
INOVA-500 "INOVA500"

Relax. delay 0.700 sec
Pulse 45.0 degrees
Acq. time 1.300 sec
Width 30188.7 Hz
23000 repetitions
OBSERVE C13, 125.7748017 MHz
DECOUPLE H1, 500.2005346 MHz
Power 41 dB
continuously on
WALTZ-16 modulated
DATA PROCESSING
Line broadening 1.0 Hz
FT size 131072
Total time 12 hr, 50 min, 3 sec

INDEX	FREQUENCY	PPM	HEIGHT
1	27176.2	~~216.070~~	130.1
2	22649.9	180.083	21.6
3	22371.2	177.867	47.4
4	22349.6	177.695	52.0
5	22026.2	175.124	35.2
6	21982.0	174.773	35.2
7	20878.8	166.001	41.0
8	13217.3	105.087	30.1
9	13201.7	104.963	45.5
10	13072.7	103.937	32.1
11	12843.8	102.117	21.4
12	12696.4	100.945	21.6
13	12259.7	97.473	18.4
14	10397.3	82.666	32.6
15	9999.8	79.505	31.4
16	9802.6	77.938	24.0
17	9571.4	76.099	40.8
18	9524.4	75.726	51.9
19	9492.6	75.473	33.1
20	9468.6	75.282	36.0
21	9219.4	73.301	29.1
22	9202.8	73.169	46.4
23	9157.2	72.807	48.0
24	9016.7	71.690	43.0
25	9001.1	71.565	43.3
26	8987.7	71.459	28.1
27	8867.0	70.499	34.3
28	8799.3	69.961	17.7
29	8779.1	69.800	32.7
30	8752.8	69.591	43.7
31	8705.8	69.217	54.4
32	8564.4	68.093	22.1
33	7891.9	62.746	47.8
34	7749.5	61.614	71.3
35	7691.5	61.153	55.5
36	7673.5	61.010	34.8
37	7662.9	60.926	76.8
38	7044.7	56.011	63.0
39	7020.3	55.817	35.9
40	6199.9	49.294	20.3
41	5081.5	40.401	50.7

INDEX	FREQUENCY	PPM	HEIGHT
42	5036.8	40.046	17.9
43	4939.6	39.273	43.9
44	4591.3	36.505	47.4
45	4556.8	36.230	59.3
46	3885.2	~~30.890~~	500.8
47	3874.1	30.802	46.3
48	3835.0	30.491	22.9
49	3630.9	28.868	36.5
50	3593.6	28.572	66.7
51	3567.3	28.363	63.7
52	3253.6	25.869	52.3
53	3246.3	25.810	75.5
54	3239.4	25.755	49.7
55	3212.2	25.539	73.7
56	2887.9	22.961	37.2
57	2852.9	22.682	25.3

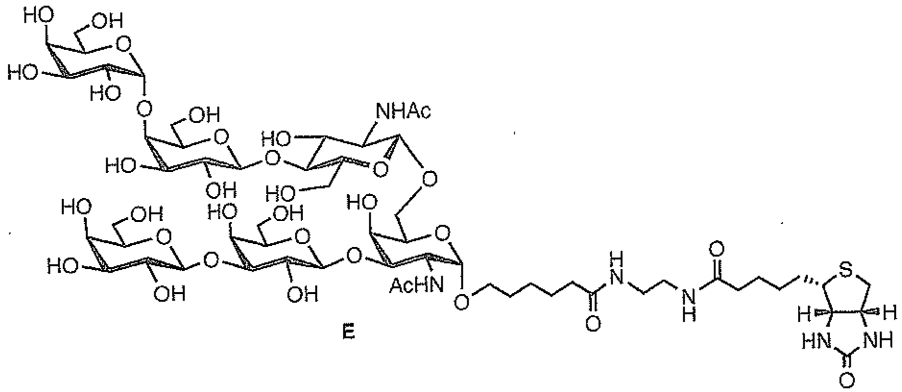

E
